# A novel SRSF3 inhibitor, SFI003, exerts anticancer activity against colorectal cancer by modulating the SRSF3/DHCR24/ROS axis

**DOI:** 10.1038/s41420-022-01039-9

**Published:** 2022-05-02

**Authors:** Yawen Zhang, Mengmeng Wang, Fanyi Meng, Man Yang, Yinshuang Chen, Xuqin Guo, Weiwei Wang, Yifan Zhu, Yundi Guo, Chunlai Feng, Shen Tian, Hongjian Zhang, Huanqiu Li, Jing Sun, Weipeng Wang

**Affiliations:** 1 Center for Drug Metabolism and Pharmacokinetics, College of Pharmaceutical Sciences, Soochow University, Suzhou, 215123 China; 2 Institute of Medical Technology, Suzhou Vocational Health College, Suzhou, 215009 China; 3 School of Pharmacy, Jiangsu University, Zhenjiang, 212013 China

**Keywords:** Target validation, Colorectal cancer, Apoptosis

## Abstract

As the modulation of serine/arginine-rich splicing factor 3 (SRSF3) may be therapeutically beneficial to colorectal cancer (CRC) treatment, the identification of novel SRSF3 inhibitors is highly anticipated. However, pharmaceutical agents targeting SRSF3 have not yet been discovered. Here, we propose a functional SRSF3 inhibitor for CRC therapy and elucidate its antitumor mechanisms. We found high expression of SRSF3 in 70.6% CRC tissues. Silencing SRSF3 markedly inhibits the proliferation and migration of CRC cells through suppression of its target gene 24-dehydrocholesterol reductase (DHCR24). This is evidenced by the links between SRSF3 and DHCR24 in CRC tissues. The novel SRSF3 inhibitor SFI003 exhibits potent antitumor efficacy in vitro and in vivo, which drives apoptosis of CRC cells via the SRSF3/DHCR24/reactive oxygen species (ROS) axis. Moreover, SFI003 is druggable with suitable pharmacokinetic properties, bioavailability, and tumor distribution. Thus, SRSF3 is a novel potential therapeutic target for CRC. Its inhibitor SFI003 may be developed as an anticancer therapeutic.

## Introduction

CRC incidence and mortality are increasing, especially among young people [1, 2]. However, successful therapy remains elusive for many patients, and only approximately 50% of patients with middle- or advanced-stage CRC can survive over 5 years [1]. Hence, the discovery of novel therapeutic targets and/or agents is urgently required for CRC treatment. Recent studies have identified that alternative splicing (AS) of messenger RNA, as a hallmark of cancer, is involved in multiple oncological processes in many types of cancers, such as colon [3], leukemia [4], breast [5], and lung [6] cancers. AS occurs in several aspects of cell biology, including cell proliferation, apoptosis, migration, invasion, and angiogenesis [7, 8]. The serine/arginine-rich (SR) RNA-binding protein family is a major class of splicing factors that play critical roles in AS. Moreover, several SR proteins have been extensively reported to be highly expressed in CRC and promote CRC progression [9, 10].

SRSF3 is an attractive member of the SR protein family. It plays critical roles in the regulation of RNA splicing and many other cellular functions [11]. Previous studies consider SRSF3 to be a proto-oncogene that is frequently overexpressed in many tumor tissues, such as colon [12], breast [13], stomach [14], and skin [15]. It has also been reported that SRSF3 regulates cell cycle arrest and apoptosis and promotes CRC cell growth by regulating the expression of ArhGAP30 [12], PKM2 [16], MAP4K4 [17], HIPK2 [18], etc. Moreover, our previous study showed that SRSF3 was involved in the splicing of B7-H3 and enhances tumor immune evasion in CRC cells [10]. This evidence suggests that targeting SRSF3 may be a promising therapeutic approach for CRC. However, a candidate pharmaceutical agent to target SRSF3 has not been discovered.

DHCR24, a pivotal enzyme of the cholesterol biosynthetic pathway, has been characterized as a reactive oxygen species (ROS) scavenger in types of cancers. DHCR24 exerts antiapoptotic effects in melanoma metastases through an oxidative stress-specific mechanism [19]. However, the role of DHCR24 in colorectal cancer is still unknown.

Here, we synthesized dozens of new small molecule compounds targeting SRSF3 and successfully identified a compound, SFI003, which exhibited significant anti-CRC activity by inducing ROS-mediated cell apoptosis. SFI003, for the first time, represents a novel SRSF3 inhibitor for potential CRC therapy. Furthermore, we demonstrated that splicing of DHCR24 pre-mRNA was SRSF3-dependent. DHCR24 was first identified as an oncogene and a potential therapeutic target for CRC treatment.

## Materials and Methods

### Cell lines and reagents

Human CRC cell lines were purchased from the American Type Culture Collection (VA, USA). HCT-116 cells were cultured in DMEM (HyClone, Utah, USA) containing 10% fetal bovine serum (FBS; Gibco, NY, USA). SW480 cells were cultured in RPMI 1640 medium (HyClone, Utah, USA) containing 10% FBS. All cells were cultured in an incubator (Thermo, CA, USA) at a constant temperature of 37 °C and 5% CO_2_. N-Acetylcysteine (NAC), Trolox, MLN4924, 5-Fu, and capecitabine were purchased from MedChemExpress LLC (Shanghai, China). MG132 was purchased from Santa Cruz Biotech (CA, USA). Chloroquine (CHQ) was purchased from Sigma (CA, USA). Rapamycin was purchased from Beyotime (Shanghai, China). Primary antibodies against SRSF3 and E-Cadherin were purchased from Abcam (Cambridge, UK). Primary antibodies against N-Cadherin, Caspase 3, DHCR24, CAT, Akt and phospho-Akt (Ser473) were purchased from Cell Signaling Technology (MA, USA). Primary antibodies against β-actin, mTOR, and phospho-mTOR (Ser2481) were purchased from Beyotime (Shanghai, China). Primary antibody against Vimentin was purchased from Proteintech (Wuhan, China). Primary antibody against Bcl-2 was purchased from Santa Cruz Biotech (CA, USA). Primary antibody against Flag was purchased from MBL Beijing Biotech (Beijing, China).

### Tissue samples

Colorectal tumors and adjacent tissues were collected from the First Affiliated Hospital of Soochow University. Human CRC tissue chips (No. HcolH180su12) were provided by Outdo Biotech (Shanghai, China). All tissues were stained with hematoxylin-eosin and confirmed by pathologists. None of the tissue-derived patients received chemotherapy or radiotherapy before surgery. This study was approved by the ethics committee of Soochow University, and all patients signed informed consent forms. All procedures performed in studies involving human participants were in accordance with the ethical standards of the ethics committee of Soochow University (No. IRB-29-20160319H)

### Homology modeling and molecular docking

The well-prepared and minimized SRSF3 homology model was constructed by using Discover Studio. The three-dimensional structures of SRSF3 were evaluated according to the probability density function value or DOPE score in view of atomic statistical potential energy. Based on the optimal three-dimensional structure of SRSF3, we then applied Glide molecular docking software to screen small molecule compounds from the databases (ChemDiv, ChemBridge, Specs, etc.) using different precision scoring functions (HTVS, SP, and XP).

### Transfection of siRNA and plasmid

SiRNAs targeting SRSF3 and DHCR24 were purchased from GenePharma (Shanghai, China). The Flag-SRSF3 overexpression vector was synthesized by GENEWIZ (Suzhou, China). HCT-116 and SW480 cells were transfected with siRNA or plasmid using Lipofectamine 2000 (Invitrogen, CA, USA) in OptiMEM (HyClone, Utah, USA) according to the manufacturer’s instructions.

### RNA sequencing

HCT-116 cells were transfected with 100 nM SRSF3 siRNA for 48 h. Then, total RNA was extracted, sequenced, and analyzed by Oebiotech (Shanghai, China).

### Reverse Transcription PCR (RT–PCR) assay

HCT-116 and SW480 cells were transfected with 100 nM siRNA for 48 h or treated with SFI003 for 72 h. Total RNA was isolated by using TRIzol (Invitrogen, CA, USA) according to the manufacturer’s instructions and then was reversely transcribed into cDNA with random primers (TaKaRa, Kusatsu, Japan) and RT-Kit (Thermo, CA, USA). The cDNA was amplified by PCR using the primers listed in Supplementary Table 1.

### Western blot (WB) analysis

After cell lysates were prepared, protein concentrations were determined by a BCA assay (TaKaRa, Kusatsu, Japan). Equal amounts of protein were separated by SDS–PAGE and transferred to polyvinylidene difluoride membranes. After normalization, the blots were probed with appropriate antibodies, as described previously [20].

### Immunohischemistry (IHC)

IHC staining was performed as described previously [10]. Briefly, the tissue chips were deparaffinized in xylene, hydrated in ethyl alcohol and washed in tap water. The adjacent sections were stained with DHCR24 antibody (CST, MA, USA) or SRSF3 antibody (Abcam, Cambridge, UK) and diaminobenzidine in an Envision System (Dako, Shanghai, China). Slides were viewed and imaged on a microscope system (Olympus, Tokyo, Japan). Two pathologists performed independent reviews of the IHC results. The stain strength was scored at 0-3, and the stain prevalence was scored at 0 (negative), 1 (1–25%), 2 (26–50%), 3 (51–75%), or 4 (76–100%). The sample with a product of stain strength and stain prevalence >6 was classified as high-expression and ≤ 6 as low-expression.

### RNA binding protein immunoprecipitation (RIP) assay

The RIP assay was performed as described previously [10]. In brief, SW480 cells were collected and washed twice with ice-cold PBS and lysed in RIP lysis buffer on ice for 30 min. The cell lysis supernatant was collected and incubated with SRSF3 (Abcam, Cambridge, UK) or IgG (Beyotime, Shanghai, China) antibodies against the RNA-binding protein of interest at 4 °C overnight. Then, protein-A/G beads were added and incubated for 4 h. The protein-A/G beads were then washed with RIP buffer five times to discard unbound material. RNA was purified for RT–PCR, and protein was isolated for WB assays.

### MTT assay

CRC cells were transfected with 100 nM siRNA or treated with various doses of SFI003 for 24 h, 48 h, or 72 h before being subjected to MTT assay as described previously [20].

### Colon formation assay

HCT-116 and SW480 cells were transfected with plasmids or siRNAs for 24 h, seeded into six-well plates (300 cells per well) and incubated at 37 °C for 2 weeks. Colonies were stained with 0.1% crystal violet. Three independent measurements of the number of colonies were performed.

### Wound-healing assay

HCT-116 and SW480 cells were seeded into six-well plates in confluent monolayers. Then, the cells were transfected with 100 nM siRNA or treated with 10 μM SFI003. Scratch wounds were created using a sterilized tip in 0.5% serum medium. Images were captured at 0 h, 24 h, and 72 h after wounding. Three independent measurements of wound width were conducted.

### Transwell migration assay

HCT-116 and SW480 cells (1 × 10^5^) were resuspended in 200 µl medium containing 0.1% serum and then added to the upper compartment of migration chambers (Costar, Corning, NY, USA). The bottom chamber was filled with 600 µl cell culture medium with 20% FBS as an attractant. After coculture for 24 h or 48 h, the cells were fixed in 4% paraformaldehyde and stained with 0.1% crystal violet. The stained cells in 8 random fields were counted at ×100 magnification. Three independent measurements were conducted.

### Apoptosis analysis

For flow cytometric analysis, the cells were treated with SFI003 (10, 20, 50 μM) for 24 h, 48 h, or 72 h or were transfected with 100 nM SRSF3 siRNA or DHCR24 siRNA for 72 h. Then, the cells were collected for Annexin V-FITC/PI staining according to the instructions from the manufacturer (MultiSciences Biotech, Hangzhou, China) and subjected to analysis on a flow cytometer (BD Biosciences, CA, USA). Three independent measurements were performed.

For Hoechst staining, after treatment with SFI003 (10, 20, 50 μM) for 72 h, the cells were fixed, washed three times with PBS, and stained with Hoechst 33258 staining solution according to the manufacturer’s instructions (Beyotime, Shanghai, China). Hoechst 33258-stained nuclei of cells were imaged by inverted fluorescence microscopy (Olympus, Tokyo, Japan). The nuclei in 8 random fields were analyzed at ×400 magnification.

### Detection of ROS

Following treatment with SFI003 or siRNA, the cells were washed with PBS and incubated with DCFH-DA for 20 min at 37 °C. After washing three times, the cells were collected and measured at 488 nm excitation and 525 nm emission by a microplate reader (Tecan, Switzerland) according to the manufacturer’s instructions (Beyotime, Shanghai, China). The protein concentrations were determined by using a BCA assay (TaKaRa, Kusatsu, Japan).

### Minigene reporter assay

A minigene reporter assay was performed as described previously [10]. In brief, minigene recombinant vectors were constructed by cloning a genomic DNA fragment containing DHCR24 exons 3-4 and a 100-bp flanking sequence into the pcDNA3.1 (+) plasmid (GeneChem, Shanghai, China). SW480 cells were co-transfected with the minigene recombinant plasmid and flag-SRSF3 plasmid. RNA was isolated, and the transcripts were determined by RT-PCR with the primers listed in Supplementary Table 1.

### CRC xenografts in SCID mice

Animal protocols were approved by the Institutional Animal Care and Use Committee at Soochow University. All animal experiments complied with the ARRIVE guidelines and were carried out in accordance with the National Institutes of Health Guide for the Care and Use of Laboratory Animals (NIH Publications No. 8023, revised 1978). Athymic male SCID mice at approximately 4-to-6 weeks old were purchased from SLAC Int. (Shanghai, China). To explore the effect of SRSF3 on the growth of CRC, approximately 3 × 10^6^ HCT-116-control or HCT-116-shSRSF3 cells were collected and mixed with Matrigel (Corning, NY, USA) at a 1:1 ratio by volume and then injected into the lower back region of mice (*n* = 5). The tumor sizes and mouse body weights were monitored every other day. At the end of the experiment, tumors were collected for further studies.

To explore the effect of SFI003 on the growth of CRC, approximately 3 × 10^6^ HCT-116 cells or 8 × 10^6^ SW480 cells were collected and mixed with Matrigel (Corning, NY, USA) at a 1:1 ratio by volume and then injected into the lower back region of mice. When tumors were palpable, mice were randomly divided into four groups. One group received vehicle as a control, while the other three groups were orally administered SFI003 at a dosage of 100 or 200 mg/kg or capecitabine at a dosage of 100 mg/kg for two continuous weeks. The tumor sizes and mouse body weights were monitored every other day. At the end of the experiment, tumors and blood samples were collected for further studies. The tumor size was evaluated according to Eq.: tumor size (mm^3^) = (length × width^2^) × 0.5.

### Statistical analysis

All data represent as mean ± SD, unless otherwise stated. Comparisons between two groups were performed using Student’s *t*-test. Comparisons among three or more groups were performed using one-way analysis of variance (ANOVA). Correlations between SRSF3/DHCR24 expression and clinical parameters were determined using Pearson’s χ^2^ method (with continuity correction when the number of patients was <5); *P* < 0.05 was considered to be statistically significant. The software Calcusyn was employed to analyze the synergetic effect of SFI003 and rapamycin. A combination index (CI) value greater than 1 indicates antagonism, whereas a CI value lower than 1 indicated synergism.

## Results

### SRSF3 promotes the proliferation and metastasis of CRC cells

To explore the oncogenic roles of SRSR3 in CRC, we detected the expression of SRSF3 in 364 CRC tissues using IHC staining analysis. Consistent with published data [10], SRSF3 was highly expressed in 257 (70.6%) CRC tissues, especially in adenocarcinoma tissues (OR = 2.30, *P* = 0.040; Supplementary Table 2), and was positively correlated with Ki67 expression, a proliferation marker (*P* = 0.040; Supplementary Table 2). Moreover, the Clinical Proteomic Tumor Analysis Consortium (CPTAC) data showed that the protein level of SRSF3 was significantly increased in CRC (Supplementary Fig. 1A).

**Fig. 1:**
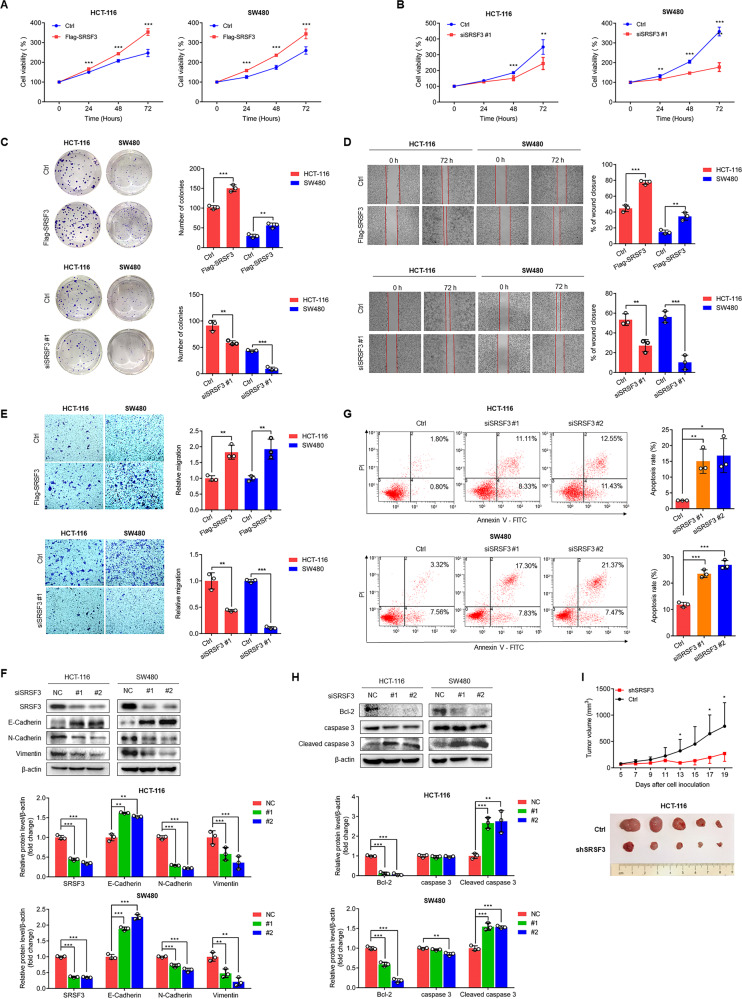
SRSF3 promoted the proliferation and migration of CRC cells. **A**, **B** MTT assays to investigate the effects of SRSF3 overexpression (**A**) or silencing (**B**) on the proliferation of CRC cells. The cells were transfected with Flag-SRSF3 or SRSF3 siRNA for 0, 24, 48 and 72 h. **C** Colony formation assays to investigate the effects of SRSF3 overexpression or silencing on the proliferation of CRC cells. The cells were respectively transfected with Flag-SRSF3 or SRSF3 siRNA for 24 h and then incubated in six-well plates for 2 weeks. Wound healing assays (**D**) and Transwell assays (**E**) to investigate the effects of SRSF3 overexpression or silencing on the migration abilities of CRC cells. The cells were respectively transfected with Flag-SRSF3 and SRSF3 siRNA for 72 or 48 h. **F** The effects of SRSF3 knockdown on the expression of EMT markers E-Cadherin, N-Cadherin, and Vimentin. **G** Annexin V-FITC/PI staining and flow cytometric analysis to investigate the effects of SRSF3 silencing on cell apoptosis. **H** The effects of SRSF3 knockdown on the expression of the apoptosis markers Bcl-2, caspase 3, and cleaved caspase 3. In Fig. **F**–**H**, the cells were transfected with siRNA control or SRSF3 siRNA for 48 h. The experiments were performed in HCT-116 and SW480 cells in Fig. A-H. (**I**) Growth curve and photos of HCT-116-shSRSF3 tumors in nude mice (*n* = 5). Data represent mean ± SD. Significance was assessed by two-sided *t*-test. ***P < 0.001; **P < 0.01; *P < 0.05.

Since SRSF3 expression is positively correlated with Ki67, we performed MTT assays and colony formation experiments to investigate the effects of SRSF3 on the proliferation ability of CRC cells. We reinforced and silenced SRSF3 in HCT-116 and SW480 cells and found that both the proliferation capacity and colony formation ability of HCT-116 and SW480 cells were evidently enhanced by reinforced SRSF3 (Fig. 1A, C, and Supplementary Fig. 2A) but were weakened by SRSF3 silencing (Fig. 1B, C, and Supplementary Fig. 2B). Due to the prevalent high expression of SRSF3 in patients with metastatic lymph nodes (OR = 1.66; Supplementary Table 2) or distant metastases (OR = 1.61; Supplementary Table 2), we performed wound-healing assays and Transwell migration assays to investigate the effects of SRSF3 on the migration ability of CRC cells. We found that the motility properties of CRC cells were enhanced by reinforced SRSF3 but weakened by the loss of SRSF3 in HCT-116 and SW480 cells (Fig. 1D, E). Furthermore, several endothelial mesenchymal transformation (EMT)-associated markers were detected by WB. We found that the mesenchymal markers N-Cadherin and Vimentin were downregulated and the epithelial marker E-Cadherin was upregulated after SRSF3 silencing in HCT-116 and SW480 cells, suggesting that the loss of SRSF3 inhibited CRC cell migration (Fig. 1F). To test whether SRSF3 had an effect on cell apoptosis, we performed Annexin V-FITC/PI dual-staining assays followed by flow cytometry analysis. We found that SRSF3 knockdown apparently induced the apoptosis of CRC cells (Fig. 1G). Additionally, we found that SRSF3 silencing attenuated Bcl-2 and elevated cleaved caspase 3, the markers of cell apoptosis, in both HCT-116 and SW480 cells. (Fig. 1H). Collectively, these findings demonstrate that loss of SRSF3 induces CRC cell apoptosis.

To further confirm the oncogenic roles of SRSF3 in CRC, we constructed HCT-116-shSRSF3 cells (Supplementary Fig. 2C) and then subcutaneously injected them into mice. We monitored the tumor volumes and weights of tumor-bearing mice every other day. We found that the growth of HCT-116-shSRSF3 xenografts was significantly suppressed, while no significant difference in body weights was observed (Fig. 1I, and Supplementary Fig. 2D, E). Notably, in contrast to the occurrence of liver damage in the four controls, none of the HCT-116-shSRSF3 mice suffered liver damage (Supplementary Fig. 2F). Considering that the liver is the most common metastatic site of CRC, the mechanism needs to be further investigated.

### SRSF3 silencing induces apoptosis of CRC cells through splicing of DHCR24 and consequent release of ROS

**Fig. 2:**
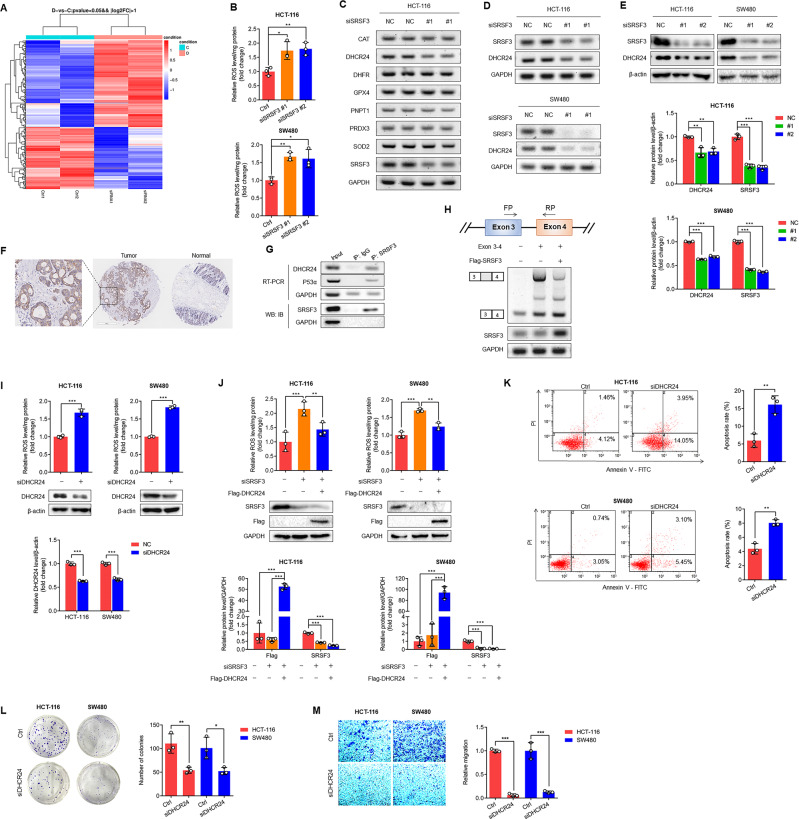
DHCR24 was responsible for SRSF3-induced ROS production. **A** RNA-seq data showed the effects of SRSF3 silencing on gene expression in HCT-116 cells, which were transfected with siRNA control or SRSF3 siRNA for 48 h. **B** The effects of SRSF3 silencing on ROS production. **C** RT–PCR results showed the effects of SRSF3 silencing on the mRNA expression of CAT, DHCR24, DHFR, GPX4, PNPT1, PRDX3 and SOD2. **D** RT–PCR results showed the effects of SRSF3 silencing on the expression of DHCR24 mRNA. **E** WB results showed the effects of SRSF3 silencing on the expression of DHCR24 protein. HCT-116 and SW480 cells were transfected with siRNA control or SRSF3 siRNA for 48 h, as shown in Fig. B-E. **F** IHC staining of DHCR24 in CRC and adjacent tissues. **G** RIP results showed the interaction between SRSF3 protein and DHCR24 mRNA. **H** Minigene assays showed the effects of SRSF3 overexpression on DHCR24 splicing. The minigene contains exons 3-4 of DHCR24. HCT-116 cells were transfected with minigene expression vector and Flag-SRSF3 for 48 h. **I** The effects of DHCR24 silencing on ROS production in HCT-116 and SW480 cells that were transfected with vehicle control or DHCR24 siRNA for 48 h. **J** The effects of DHCR24 overexpression on SRSF3 silencing-induced ROS production in HCT-116 and SW480 cells that were transfected with SRSSF3 siRNA alone or co-transfected with Flag-DHCR24 for 48 h. **K** Annexin V-FITC/PI staining and flow cytometric analysis to investigate the effects of DHCR24 silencing on the apoptosis of HCT-116 and SW480 cells that were transfected with vehicle control or DHCR24 siRNA for 72 h. **L** Colony formation assays for investigating the effects of DHCR24 knockdown on the proliferation of HCT-116 and SW480 cells that were transfected with DHCR24 siRNA for 24 h and then incubated in six-well plates for 2 weeks. **M** Transwell migration assays for investigating the effects of DHCR24 knockdown on the migration of HCT-116 and SW480 cells that were transfected with DHCR24 siRNA for 48 h. Each experiment was performed in triplicate. Data represent mean ± SD. Significance was assessed by two-sided *t* test. ***P < 0.001; **P < 0.01; *P < 0.05.

To explore the mechanisms underlying SRSF3-induced cell apoptosis, we sequenced transcripts in HCT-116 cells after silencing SRSF3 (Fig. 2A). We performed KEGG pathway enrichment analysis of the genes downregulated by SRSF3 silencing. We found that most genes were enriched in metabolic pathways (Supplementary Fig. 1B), suggesting regulatory roles of SRSF3 in metabolism. Metabolic stress exists in tumor microenvironments and induces cell death through ROS-induced apoptosis [21]. Moreover, ROS are one of the most important regulators of cell apoptosis [22], so we investigated the effects of SRSF3 on the production of ROS. As expected, we found that SRSF3 silencing markedly elevated the level of ROS in HCT-116 and SW480 cells (Fig. 2B). The effects of SRSF3 on the expression of ROS-related genes were further confirmed by RT–PCR assays. We found that DHCR24 expression was obviously suppressed by SRSF3 knockdown in HCT-116 cells (Fig. 2C). Further investigation showed that both DHCR24 mRNA and protein were downregulated by SRSF3 knockdown (Fig. 2D, E). To determine this, we first detected DHCR24 expression in 364 CRC tissues by IHC staining. We found high expression of DHCR24 in 281 (77.2%) CRC tissues (Fig. 2F; Supplementary Table 3), especially adenocarcinoma tissues (OR = 2.38, *P* = 0.036; Supplementary Table 3). Notably, DHCR24 expression was positively correlated with SRSF3 and CA199, a tumor marker, in this corpus of CRC tissues (Supplementary Table 3). In addition, high expression of DHCR24 and a positive correlation between DHCR24 and SRSF3 were also observed in both CPTAC and TCGA samples (Supplementary Fig. 1C, D), suggesting a possible SRSF3-mediated regulation of DHCR24.

Then, we performed RIP assays to assess the involvement of SRSF3 in the splicing of DHCR24. Since the TP53 gene has been identified as a target gene of SRSF3 [23], we utilized it as a positive control to evaluate the splicing efficiency of SRSF3. We detected DHCR24 mRNA in the SRSF3 protein-antibody-bead system, demonstrating direct binding of the SRSF3 protein to DHCR24 mRNA (Fig. 2G). Furthermore, we constructed a minigene recombinant plasmid containing exons 3-4 of DHCR24 and transfected it with the SRSF3 expression vector into SW480 cells. We found that the retention of intron between exons 3 and 4 was inhibited by reinforced SRSF3 (Fig. 2H), indicating that SRSF3 promoted the splicing of DHCR24 exons.

Although DHCR24 exerts an antiapoptotic function as a ROS scavenger in murine neuroblastoma cells [24] and melanoma cells [19], the roles of DHCR24 in CRC remain largely unknown. To address this point, we measured the ROS level after silencing DHCR24 in HCT-116 and SW480 cells. We found that the ROS level was significantly increased by DHCR24 silencing (Fig. 2I). Moreover, SRSF3 silencing-induced ROS were reduced by DHCR24 reinforcement (Fig. 2J). These findings demonstrate that DHCR24 is involved in the SRSF3 silencing-induced release of ROS, which drives us to explore the oncogenic roles of DHCR24 in CRC. Considering that DHCR24 was highly expressed in CRC and DHCR24 silencing induced the release of ROS, we speculated that DHCR24 silencing may promote cell apoptosis. Indeed, when we silenced DHCR24 in HCT-116 and SW480 cells, the apoptosis of the cells was apparently exacerbated (Fig. 2K). To test the possible contribution of DHCR24 to CRC progression, we performed colony formation assays and Transwell migration assays and found that DHCR24 silencing evidently prevented the proliferation and migration of HCT-116 and SW480 cells (Fig. 2L, M). Taken together, these findings demonstrate that SRSF3 stimulates ROS generation through splicing of DHCR24, which may function as an oncogene in CRC cells.

### Discovery of SRSF3 inhibitor SFI003

**Fig. 3:**
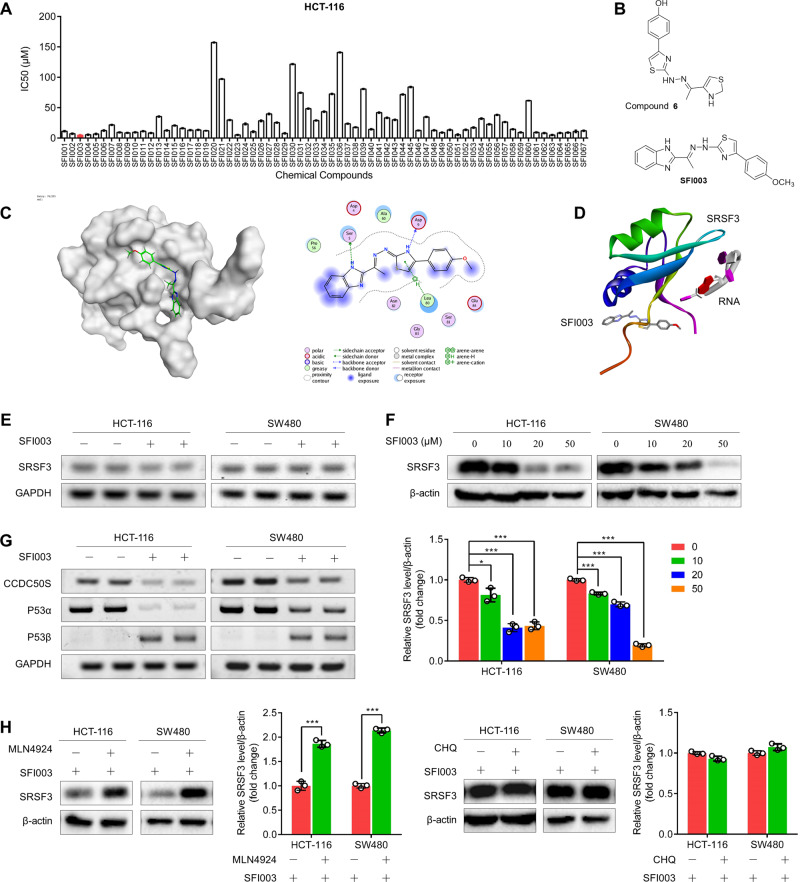
Discovery of SRSF3 inhibitor SFI003. **A** The IC50 values of chemical compounds in HCT-116 cells. The cells were treated with the indicated doses of chemical compounds for 72 h. **B** The chemical structures of compound 6 and SFI003. **C** The simulated binding modes of SFI003 to the SRSF3 protein with three bonds at residues Ser5, Asp9, and Leu80. **D** The simulated binding modes of SFI003 and the RNA CAUC, a predicted binding site of SRSF3, on the SRSF3 protein. **E** RT–PCR results showed the effects of SFI003 (20 μM) on SRSF3 mRNA expression. **F** WB results showed the effects of various doses of SFI003 on SRSF3 protein expression. **G** RT–PCR results showed the effects of SFI003 on the mRNA expression of CCDC50S, P53α, and P53β. HCT-116 and SW480 cells were treated with SFI003 at 20 μM for 72 h. **H** The effects of MLN4924 and CHQ on SFI003-induced SRSF3 degradation in HCT-116 and SW480 cells. The cells were treated with 20 μM SFI003 alone for 72 h, 20 μM SFI003 for 60 h and 3 μM MLN4924 or 50 μM CHQ for an additional 12 h. Data represent mean ± SD. Significance was assessed by two-sided *t* test. ***P < 0.001; **P < 0.01; *P < 0.05.

The fact that SRSF3 exerted oncogenic roles in CRC through splicing of DHCR24 and generation of ROS drove us to explore whether SRSF3 could be used as a therapeutic target in CRC. We designed a hierarchical strategy that combines cascade docking, experimental assays, and chemical optimization to screen and identify potential SRSF3 inhibitors. We first constructed a well-prepared and minimized SRSF3 homology model to screen compounds from commercial databases containing more than 1,500,000 compounds. Based on the predicted drug-likeness properties, REOS filtering, and core scaffold clustering, we selected 22 diverse compounds for the following biological experimental assays. Among them, compound 6 displayed satisfactory inhibitory activity against SRSF3. Taking compound 6 as a lead compound, we designed and chemically synthesized dozens of derivatives. We detected the inhibitory activities of these compounds in HCT-116 cells and found that SFI003 was the most potent compound with the lowest IC_50_ value (Fig. 3A, B).

To examine whether SFI003 bound to SRSF3, we in silico predicted the binding of SFI003 to the SRSF3 protein. The results demonstrated that SFI003 bound to residues Ser5, Asp9, and Leu80 of SRSF3 (Fig. 3C). The docking score of SFI003 is −5.4390178. Moreover, we found that the predicted binding site of SFI003 was distant from the binding site of pre-mRNA to SRSF3 protein (Fig. 3D), suggesting that SFI003 may not disrupt the binding of SRSF3 to target mRNAs. Another possibility was that SFI003 blocked SRSF3 activities by directly reducing its expression level. To address this, we tested the impacts of SFI003 on SRSF3 expression. We treated HCT-116 and SW480 cells with SFI003 and unexpectedly found that SFI003 apparently reduced the level of SRSF3 protein instead of RNA, suggesting that SFI003 may directly affect the degradation of SRSF3 protein instead of impeding the transcription of SRSF3 (Fig. 3E, F). As a result, SFI003 markedly reduced the mRNA levels of CCDC50S and p53α and elevated the mRNA level of p53β (Fig. 3G), two verified target genes of SRSF3 [23, 25], suggesting that SFI003 had a functional effect on RNA splicing of SRSF3 target genes. To investigate the mechanisms underlying SFI003-induced SRSF3 degradation, we treated HCT-116 and SW480 cells with SFI003 and inhibitors against proteasomes or lysosomes, including MG132, MLN4924, and CHQ. Interestingly, we found that SFI003-induced SRSF3 degradation was rescued by MLN4924, a neddylation inhibitor, and MG132, a proteasome inhibitor rather than CHQ, an inhibitor of lysosomes, in HCT-116 and SW480 cells (Fig. 3H and Supplementary Fig. 2G), indicating that SFI003 led to neddylation-dependent SRSF3 degradation in HCT-116 and SW480 cells. This was in agreement with previous reports on neddylation-dependent SRSF3 degradation [26, 27]. Taken together, these findings suggest that SFI003 disrupts SRSF3-mediated splicing by reducing its protein level in a neddylation-dependent manner.

### SFI003 inhibits the proliferation and migration of CRC cells

**Fig. 4:**
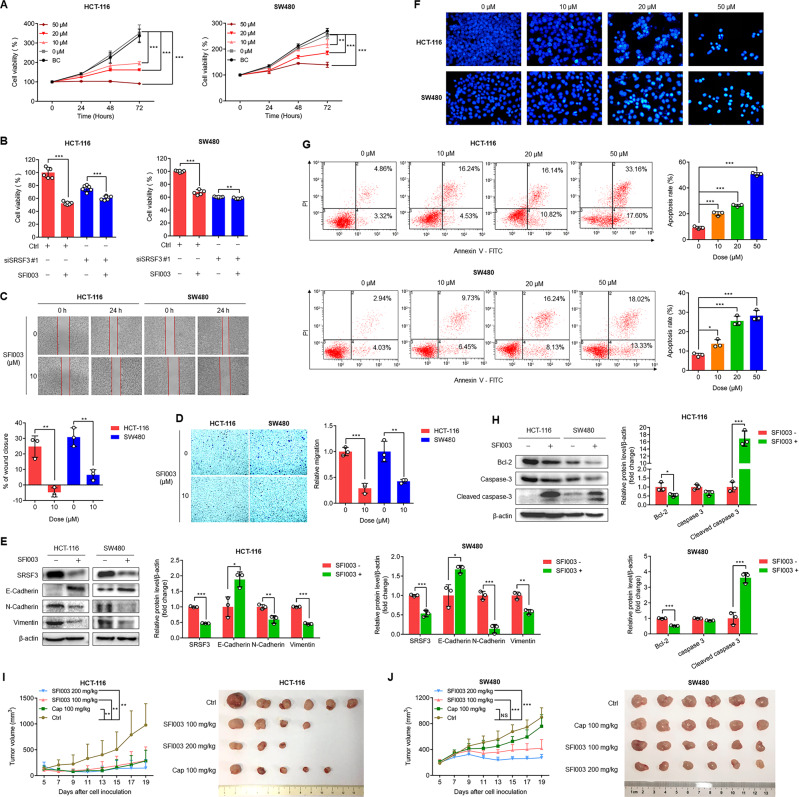
SFI003 inhibited the migration and induced the apoptosis of CRC cells. **A** MTT assays for investigating the effects of SFI003 on cell viability. The cells were treated with the indicated doses of SFI003 for 24, 48 and 72 h. **B** The effects of SRSF3 silencing on SFI003-suppressed cell viability. HCT-116 and SW480 cells were transfected with siRNA control or SRSF3 siRNA for 24 h and then treated with SFI003 at 10 μM in HCT-116 cells or 50 μM in SW480 cells for 72 h. Wound healing assays (**C**) and Transwell assays (**D**) to investigate the effects of SFI003 on the migration abilities of HCT-116 and SW480 cells transfected with 10 μM SFI003 for 24 h. **E** The effects of SFI003 on the expression of EMT markers E-Cadherin, N-Cadherin, and Vimentin. **F** Hoechst staining assays to investigate the effects of SFI003 on cell apoptosis. **G** Annexin V-FITC/PI staining and flow cytometric analysis to investigate the effects of SFI003 on cell apoptosis. (**H**) The effects of SFI003 on the expression of the apoptosis markers Bcl-2, Caspase 3, and cleaved Caspase 3. HCT-116 and SW480 cells were treated with SFI003 at 20 μM (Fig. **E** and **H**) or the indicated doses (Fig. **F** and **G**) for 72 h. Growth curves and photos of HCT-116 (**I**) and SW480 (**J**) xenografts treated with SFI003 (100, 200 mg/kg) or capecitabine (100 mg/kg). Each experiment was performed in triplicate. Data represent mean ± SD. Significance was assessed by two-sided *t* test. ***P < 0.001; **P < 0.01; *P < 0.05.

Since SFI003 was capable of promoting SRSF3 degradation, we examined the cytotoxicity of SFI003 on CRC cells. We treated HCT-116 and SW480 cells with SFI003 at various doses for different times and found that SFI003 inhibited the proliferation of these cells in dose- and time-dependent manners (Fig. 4A). SFI003 inhibited the proliferation of HCT-116 and SW480 cells with IC_50_ values of 8.78 µM and 48.67 µM, respectively (Supplementary Fig. 3A). When we silenced SRSF3 in HCT-116 and SW480 cells before treatment with SFI003, the cytotoxicity of SFI003 was eliminated, indicating that SRSF3 was required for SFI003-induced cell death (Fig. 4B). Consistent with the SRSF3 silencing-induced apoptosis and weakened migration capacity of CRC cells, SFI003 repressed the migration (Fig. 4C–E) and promoted apoptosis of CRC cells (Fig. 4F–H). These findings demonstrate that SFI003 is effective in inhibiting the proliferation and migration of CRC cells.

To further investigate the pharmacological roles of SFI003 in vivo, we subcutaneously injected HCT-116 and SW480 cells into mice. After palpable tumors formed, we orally administered SFI003 or capecitabine, a clinical first-line treatment for CRC, to tumor-bearing mice for two weeks. We found that SFI003 efficiently suppressed the growth of HCT-116 and SW480 xenografts in a dose-dependent manner (Fig. 4I, J and Supplementary Fig. 3B). Moreover, similar antitumor activities were observed between SFI003 and capecitabine in HCT-116 xenografts. Notably, two 100 mg/kg SFI003-treated tumors and three 200 mg/kg SFI003-treated tumors disappeared, while only one tumor treated with 100 mg/kg capecitabine was lost (Fig. 4I). Interestingly, SFI003 showed stronger inhibitory activity than capecitabine against SW480 tumors, although a slight reduction in body weight was observed (Supplementary Fig. 3B). This might result from a high concentration of SFI003 in tumors close to the plasma of tumor-bearing mice (Supplementary Fig. 3C). These findings demonstrate that SFI003 notably displays potent antitumor activity comparable to capecitabine in CRC. Finally, we evaluated the druggability of SFI003, and found that SFI003 is well tolerated in vivo (Supplementary Table 4; Supplementary Fig. 3D–F).

### SFI003 induces CRC cell apoptosis through the SRSF3/DHCR24/ROS axis

**Fig. 5:**
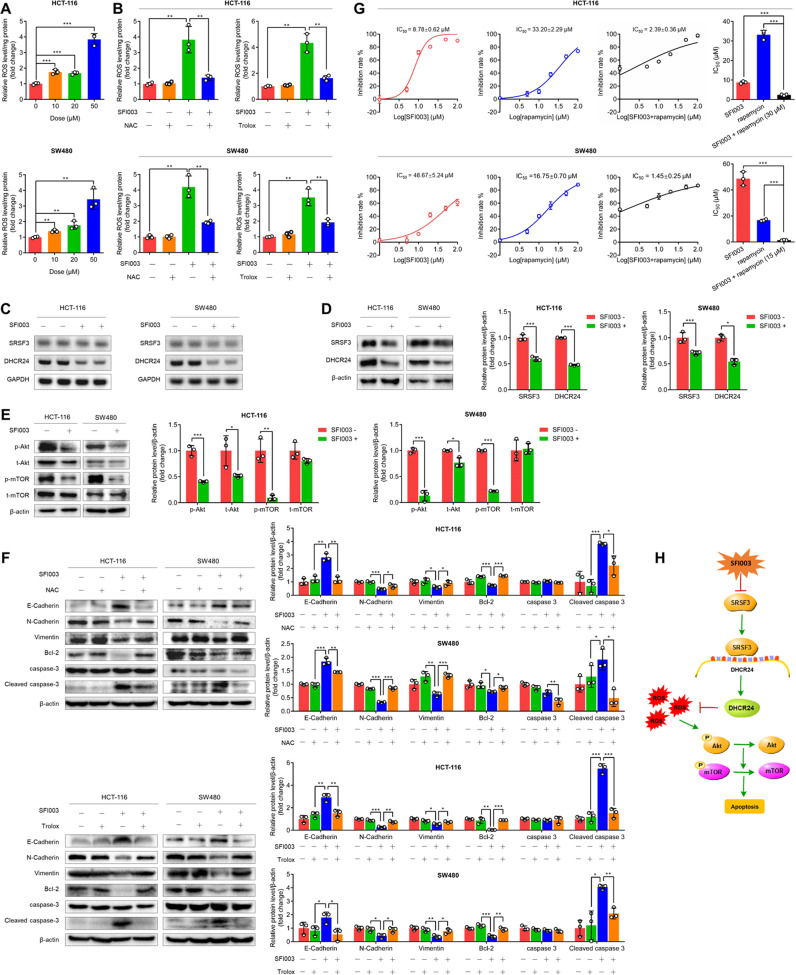
SFI003 inhibited cell growth through modulation of the SRSF3/DHCR24/ROS axis. **A** The effects of SFI003 on ROS production. HCT-116 and SW480 cells were treated with SFI003 at 10, 20, and 50 μM for 72 h. **B** The effects of NAC and Trolox on SFI003-induced ROS production. HCT-116 and SW480 cells were treated with 20 μM SFI003 and/or 5 mM NAC or 100 μM Trolox for 72 h. The effects of SFI003 on the mRNA (**C**) and protein (**D**) expression of SRSF3 and DHCR24. (**E**) The effects of SFI003 on p-Akt, Akt, p-mTOR, and mTOR protein expression. (**F**) The effects of SFI003 on the expression of EMT markers E-Cadherin, N-Cadherin, and Vimentin, as well as apoptosis markers Bcl-2, Caspase 3, and cleaved Caspase 3. Cells were treated as in (**B**). In Fig. (**C**–**F**), HCT-116 and SW480 cells were treated with 20 μM SFI003 for 72 h. (**G**) The inhibition curves of SFI003, rapamycin, or in combination in HCT-116 and SW480 cells. The cells were treated with the indicated doses of SFI003, rapamycin, or in combination for 72 h. **H** Schematic diagram of the proposed mechanisms underlying SFI003-induced cell apoptosis. Each experiment was performed in triplicate. Data represent mean ± SD. Significance was assessed by two-sided *t* test. ***P < 0.001; **P < 0.01; *P < 0.05.

Given that SRSF3 knockdown attenuated DHCR24 expression and consequently induced ROS generation and cell apoptosis, we speculated that SFI003-induced apoptosis was due to DHCR24 suppression and consequent ROS generation. As expected, ROS were significantly enhanced by SFI003 in a dose-dependent manner in both HCT-116 and SW480 cells (Fig. 5A). We treated cells with the general ROS scavengers N-Acetylcysteine (NAC), a precursor of reduced glutathione, and Trolox, a derivative of the antioxidant vitamine E, and found that both of them significantly reversed SFI003-induced ROS (Fig. 5B). Moreover, we found that both DHCR24 mRNA and protein were inhibited by SFI003 in HCT-116 and SW480 cells (Fig. 5C, D). Since the Akt-mammalian target of rapamycin (mTOR) pathway is a well-known downstream signal transduction pathway of ROS [21], we explored the effects of SFI003 on this pathway and found that the activities of Akt (p-Akt) and mTOR (p-mTOR) were inhibited by SFI003 in HCT-116 and SW480 cells (Fig. 5E). To further confirm that SFI003 exerts antitumor activity through mTOR pathway, we investigated the effects of an mTOR inhibitor, rapamycin, on the cytotoxicity of SFI003 in CRC cells. We measured the IC_50_ values of SFI003, rapamycin, or in combination in HCT-116 and SW480 cells. We found that the IC_50_ values were 8.78 µM and 48.67 µM for SFI003 alone and 33.20 µM and 16.75 µM for rapamycin alone in HCT-116 and SW480 cells, respectively (Fig. 5G). The IC_50_ values of SFI003 in combination with rapamycin were decreased from 8.78 µM to 2.39 µM in HCT-116 cells and from 48.67 µM to 1.45 µM in SW480 cells (Fig. 5G). Moreover, synergetic effects between SFI003 and rapamycin with CI values less than 1 were observed in both HCT-116 and SW480 cells (Supplementary Table 5, Supplementary Fig. 3G). In addition, we found that SFI003-induced apoptosis and migration disability of CRC cells were reversed by NAC and Trolox (Fig. 5F). These findings demonstrate that SFI003 induces CRC cell apoptosis through the SRSF3/DHCR24/ROS axis (Fig. 5G).

## Discussion

Recent studies have identified that high expression of SRSF3 contributes to tumorigenesis by promoting cancer cell proliferation, migration, invasion, and metastasis [28]. Meanwhile, SRSF3 also functions as an oncogene by regulating the cell cycle, apoptosis, energy metabolism, and immune escape in CRC cells [28]. However, the molecular details concerning the mechanism by which SRSF3 silencing induces CRC cell apoptosis remain to be further investigated. Our findings further support the oncogenic roles of SRSF3 in the promotion of CRC cell proliferation in vitro and in vivo. Here, we, for the first time, identified DHCR24 as a target gene of SRSF3, which partially contributes to the oncogenic functions of SRSF3. Moreover, we synthesized a novel SRSF3 inhibitor, SFI003, which showed significant inhibitory effects on CRC progression. Accumulated evidence has shown that SRSF3 acts as an oncogene in CRC by impeding cell cycle arrest and apoptosis [18]. In this study, we confirmed that SRSF3 was upregulated in CRC tissues, especially adenocarcinoma tissues, and positively correlated with Ki67. In addition, we found that SRSF3 silencing induced cell apoptosis via ROS generation. ROS are one of the most important regulators of apoptosis in cancer cells [29, 30]. Excessive high levels of ROS induce cancer cell apoptosis, showing an anticancer role [31, 32]. ROS are commonly higher in CRC cells than in their normal counterpart cells [33, 34]. Therefore, it is possible that ROS induce cell apoptosis while not affecting normal cells, demonstrating fewer side effects [34]. During this process, DHCR24 has been identified as an important suppresser of ROS regulation [35]. Here, we demonstrate for the first time that SRSF3 silencing induces CRC cell apoptosis by promoting ROS generation, which is attributed to the splicing of DHCR24. Furthermore, DHCR24 is highly expressed in CRC tissues, suggesting that DHCR24 works as an oncogene and potential therapeutic target for CRC treatment. DHCR24 expression is positively correlated with SRSF3 and CA199 in CRC tissues. Additionally, high expression of SRSF3 and DHCR24 was detected in other types of cancers, such as uterine corpus endometrial carcinoma and lung adenocarcinoma, suggesting that further investigations are required on the antitumor effects and underlying mechanisms of SFI003 in these cancers.

CAT, a critical scavenger of hydrogen peroxide, is also undergoing splicing [36]. We hypothesized that CAT inhibition might also be responsible for SRSF3 silencing-induced ROS generation. Unexpectedly, SRSF3 had no obvious effect on either the mRNA or protein levels of CAT (Supplementary Fig. 2H and 2I). Moreover, the TCGA database showed that there was no significant relationship between SRSF3 and CAT expression in CRC clinical samples (Supplementary Fig. 1E). Collectively, these findings suggest that CAT is not a target of SRSF3 and that SRSF3-stimulated ROS might not result from splicing of CAT.

TP53, a target gene of SRSF3, was utilized as a positive control for the evaluation of SRSF3-mediated splicing in the current study. The TP53 gene can be translated into at least 12 isoforms, and the longest isoform encodes the canonical p53 protein, also termed p53α, which is the most abundant p53 isoform [37]. Of these p53 isoforms, ∆133p53α and p53β are best characterized as endogenous regulators of cellular senescence [38]. P53β functions as a tumor suppressor that induces cancer cell senescence and apoptosis [37]. It has been reported that down-regulation of SRSF3 drives p53α isoform splicing towards the p53β isoform through inducing the inclusion of exon i9 [23]. Therefore, inhibition of SRSF3 retards tumor growth primarily through inducing p53β [39]. In the current study, we also confirmed that SRSF3 knockdown attenuated the mRNA level of p53α and elevated the mRNA level of p53β in HCT-116 and SW480 cells, although the TCGA data showed that there was no significant correlation between P53 and SRSF3 expression in CRC clinical samples (Supplementary Fig. 1 F and 2H). These findings suggest that SRSF3 inhibition leads to CRC cell apoptosis at least partly through induced p53β.

An inhibitor of SRSF6, indacaterol, has been identified to block CRC progression [40]. Thus, SRSF3 inhibitors are highly anticipated for CRC treatment [28]. Indeed, the novel SRSF3 inhibitor SFI003 effectively inhibits the proliferation of CRC cells by inducing cell apoptosis both in vitro and in vivo. SFI003 could bind to the SRSF3 protein, thereby attenuating the protein level of SRSF3 by inducing SRSF3 degradation in a neddylation-dependent manner. Unexpectedly, SFI003 shows a more potent antitumor efficacy than capecitabine in SW480 xenografts, and two of six HCT-116 xenografts were completely suppressed by 100 mg/kg SFI003. Furthermore, SFI003 achieves good bioavailability, tumor distribution, good tolerance, and drug-likeness properties. These findings indicate SFI003 as a candidate therapeutic inhibitor of SRSF3 for CRC treatment.

In summary, we have identified a novel therapeutic target, SRSF3, and its novel oral inhibitor SFI003, which inhibits cell growth and induces cell apoptosis through the SRSF3/DHCR24/ROS axis. Moreover, this axis is characterized for the first time as an apoptosis-related pathway for cancer therapy, which is responsible for SFI003-induced apoptosis and excessive ROS generation. Therefore, our study offers novel insights into the oncogenic roles of SRSF3 and provides strong evidence to support SFI003 as an anti-CRC compound by targeting SRSF3.

## Supplementary information

Supplementary Materials

Original western blots

## Data Availability

All data needed to evaluate the conclusions in the paper are present in the paper and/or the Supplementary Materials. Additional data related to this paper may be requested from the authors.
