# Supplementary material for: A novel SRSF3 inhibitor, SFI003, exerts anticancer activity against colorectal cancer by modulating the SRSF3/DHCR24/ROS axis
Source: Cell Death Discov. 2022 May 2;8:238. doi: 10.1038/s41420-022-01039-9 (PMC9061822; doi:10.1038/s41420-022-01039-9)
Supplement: Supplementary file 1 — Supplementary Materials [file 41420_2022_1039_MOESM1_ESM.pdf]

Supplementary Materials

Methods

Chemical synthesis of SFI003

The chemical synthesis process of SFI003 is shown in Supplementary Fig. 4. (i) O-phenylenediamine (1.0 equiv) and lactic acid (1.2 equiv) were added to 4 M HCl (5.0 volumes). The mixture was stirred at 90 °C for 1.5 h and then cooled at room temperature. The pH was adjusted to 10-11 by ammonia, and then the mixture was filtered to get compound **1**. (ii) Compound **1** (1.0 equiv) was added to acetic acid (5.0 volumes) and heated to 90 °C. CrO_3 (2.0 equiv) was dissolved in water and then added dropwise to the mixture. The mixture was stirred at 105 °C for 0.5 h and then cooled at room temperature. Ethyl acetate and water was added to the solution. The aqueous layer was extracted with ethyl acetate (3×). The organic layers were gathered, washed with brine, dried over Na_2SO_4, filtered, and evaporated under reduced pressure. The crude product was purified by column chromatography (ethyl acetate/petroleum ether, 1/10) to give compound **2**. (iii) To a mixture of compound **2** (1.0 equiv), thiosemicarbazide (1.2 equiv) and EtOH (5.0 volumes), a few drops of acetic acid were added. The mixture was stirred at 80 °C for 6 h and then cooled at room temperature. The mixture was filtered to get pure compound **3**. (iv) To a mixture of compound **3** (1.0 equiv), 2-bromo-4'-methoxyacetophenone (1.0 equiv) and EtOH (5.0 volumes), a few drops of acetic acid were added. The mixture was stirred at room temperature for 0.5 h. The mixture was filtered, and the precipitate was dried to get the target compound. The chemical structure of SFI003 was identified by ^{1}H NMR and ^{13}C NMR spectra.

Pharmacokinetics and tissue distribution of SFI003 in mice

The male ICR mice (n=3) were orally (p.o.) or intravenously (i.v.) administrated with 100mg/kg and 10mg/kg SFI003, respectively. The blood samples of the mice were collected via tail vein at 0.25, 0.5, 1, 2, 4, 8 and 24 h after p.o. administration, or at 0.083, 0.25, 0.5, 1, 2, 4, 8 and 24 h after i.v. injection. The plasma were separated from

the blood samples by centrifuging at 5,000 rpm under 4℃ for 5 min. For determination of SFI003 concentration in mice tissues, the male ICR mice (n=3) were orally administrated with 100mg/kg SFI003, and the liver and kidney of the mice were collected at 0.25, 0.5, 1, 2, 4, 8, and 12 h after administration. An aliquot of 25 μl plasma or tissue sample was added into 100 μl acetonitrile containing internal standard Tolbutamide. The mixture was vortexed for 3 min and centrifuged at 13,000 rpm for 10 min. An aliquot of 10 μl resulting supernatant was injected to LC-MS/MS system (API4000 QTrap; AB Sciex) for compound analysis. The analytes were separated in a Venusil XBP-C18 column (2.1×50 mm, 5 μm; Agela, China) using the mixture of 5mM NH4OAc-0.1% formic acid (A) and methanol (B) as mobile phase. The gradient condition was: 1.3 min, 2%B; 2.0 min, 90%B; 3.5 min, 90%B; 3.8 min, 2%B. The elutes were detected by MS/MS spectrometry using ESI positive mode with m/z 271.1>172.2, DP 70V, and CE 18V for Tolbutamide, and m/z 364.0>206.2, DP 56V, and CE 32V for SFI003. The pharmacokinetics parameters were calculated by using Phoenix WinNolin 8.2.

Results

Identification of SFI003 structure

The identification results of SFI003 structure was: ^{1}H NMR (600 MHz, DMSO) δ 7.81 – 7.73 (m, 4H), 7.50 (dd, J = 6.0, 3.0 Hz, 2H), 7.31 (s, 1H), 6.97 (d, J = 8.6 Hz, 2H), 3.76 (s, 3H), 2.46 (s, 3H); ^{13}C NMR (151 MHz, DMSO) δ 148.06, 140.83, 134.17, 133.70, 132.83, 129.20, 128.88, 128.81, 128.37, 126.19, 126.01, 114.59, 106.23, 56.46, 55.73, 40.42, 40.30, 40.16, 40.02, 39.88, 39.74, 39.60, 39.46, 18.97, 14.10.

Druggability evaluation of SFI003

Next, we evaluated the druggability of SFI003. We first examined the pharmacokinetic properties of SFI003 in mice. We measured the plasma concentration of SFI003 in mice after oral administration of 100 mg/kg SFI003. We found that the

plasma concentration of SFI003 reached a maximum at 4436.67 ng/ml at 2 h after administration (Supplementary Table 3; Supplementary Fig. 3D). We then intravenously injected 10 mg/kg SFI003 into mice and measured the plasma concentration of SFI003, on which we calculated the bioavailability of SFI003 to be 52.51% (Supplementary Table 3; Supplementary Fig. 3D). Then, we evaluated the tissue distribution of SFI003 in mice by determining the SFI003 concentration in the plasma, liver, and kidney after oral administration of 100 mg/kg SFI003. We found that SFI003 was highly accumulated in the liver and kidney (Supplementary Fig. 3E). Consequently, we biochemically analyzed the serum levels of functional markers for the liver and kidney, including alkaline phosphatase (ALP), alanine aminotransferase (ALT), aspartate aminotransferase (AST), creatinine (CRE), gamma-glutamyl transpeptidase (GGT), and urea (URE), in SFI003-treated mice. Except for moderate changes in CRE and URE, no alteration of ALP, ALT, AST, or GGT was observed for SFI003 (Supplementary Fig. 3F). These findings suggest that SFI003 is well tolerated *in vivo*.

We further tested the metabolic stability of SFI003 in human or rat liver microsomes. We found that the half times were 31.50 min and 53.31 min, and the intrinsic clearances were 3.31 and 2.80 L/h/kg in human and rat liver microsomes, respectively. In addition, SFI003 is druggable with a suitable molecular weight of 363.4362, 4 aliphatic and aromatic rings, 6 rotatable bonds, 2 hydrogen bond donors, 6 hydrogen bond receptors, and a plasma protein binding rate of 41.4%.

Tables

Table 1. The primers for RT-PCR

Gene	Primers	Product size (bp)
CAT	Forward: 5’-TCCCATCGCAGTTCGGTTCT-3’	133
	Reverse: 5’-GGGGGTGTTATTTCCAACGAG-3’	
CCDC50S	Forward: 5’-GCTGGCTATTGAGGCAGAG-3’	178
	Reverse: 5’-TGGCTTCATTCCTCCATCTT-3’	
DHCR24	Forward: 5’-ATGGAAGGAGCAGGGTAGCA-3’	168
	Reverse: 5’-AAGGGCTCCACACGGACAAT-3’	
DHFR	Forward: 5’-AGAACATGGGCATCGGCAAG-3’	143
	Reverse: 5’-GGAGAACCAGGTCTTCTTACCC-3’	
GAPDH	Forward: 5’-TGCACCACCAACTGCTTAGC-3’	87
	Reverse: 5’-GGCATGGACTGTGGTCATGAG-3’	
GPX4	Forward: 5’-ATGCACGAGTTTTCCGCCAA-3’	117
	Reverse: 5’-GTTTACTTCGGTCTTGCCTCAC-3’	
p53α	Forward: 5’-CTCTGACTGTACCACCATCCACTA-3’	373
	Reverse: 5’-GAGTTCCAAGGCCTCATTCAGCTC-3’	
p53β	Forward: 5’- CTTTGAGGTGCGTGTTTGTGC-3’	205
	Reverse: 5’- TTGAAAGCTGGTCTGGTCCTGA-3’	
PNPT1	Forward: 5’-CTGAGCGATGGTCCTTTCCT-3’	217
	Reverse: 5’-TTTTACTGACCGCTGTGACC-3’	
PRDX3	Forward: 5’-TGGAAGAAACCCTCCGCT-3’	119
	Reverse: 5’-TTTGGAAGCAGCTGGACTTG-3’	
SOD2	Forward: 5’-TCACCGAGGAGAAGTACCAGGA-3’	380
	Reverse: 5’-GTAGTAAGCGTGCTCCCACA-3’	
SRSF3	Forward: 5’-GGAAACAATGGCAACAAGACGG-3’	79
	Reverse: 5’-TTCTAGCAACCCACACACTTCG-3’	
minigene	Forward: 5’-CTGCTGACCTCCATTGGCTG-3’	139
exon3-4	Reverse: 5’-CGTAAGCAGTGCAGATGTGTTGG-3’	

Table 2. Correlation between SRSF3 expression and clinicopathological characteristics

	variables	SRSF3 expression		OR (95%CI)	p value
		low	High		
Gender	Male	51	143	0.82 (0.51-1.30)	0.407
	Female	48	110		
Age (year)	≤60	31	79	0.87 (0.52-1.46)	0.692
	>60	56	124		
Location	Colon	60	149	0.96 (0.61-1.51)	0.908
	Rectum	47	112		
Tumor size	≤5 cm	65	176	0.77 (0.48-1.22)	0.277
	>5 cm	41	85		
Tumor volume	≤25 cm^3	58	133	1.16 (0.74-1.83)	0.565
	>25 cm^3	48	128		
Adenocarcinoma	Yes	92	240	2.30 (1.10-4.80)	**0.040**
	No	15	17		
T stage	T1/T2	21	51	1.00 (0.57-1.76)	1.000
	T3/T4	85	207		
N stage	N0	61	151	0.96 (0.61-1.52)	0.907
	N1/N2	45	107		
Metastatic lymph nodes	≤2	30	58	1.66 (0.80-3.43)	0.208
	>2	15	48		
M stage	M0	101	239	1.61 (0.58-4.42)	0.487
	M1	5	19		
TNM stage	I/II	59	148	0.93 (0.59-1.47)	0.816
	III/IV	47	110		
Borrmann type	I	28	77	0.68 (0.33-1.42)	0.344
	II	17	32		
	III	34	76	0.81 (0.38-1.70)	0.567
	IV	15	27		
Ki67	Low	23	33	2.99 (1.12-7.96)	**0.040**
	High	7	30		
P53	Low	16	28	1.43 (0.60-3.42)	0.507
	High	14	35		
MSH2	Low	6	9	1.50 (0.48-4.69)	0.551
	High	24	54		
MSH6	Low	15	22	1.86 (0.77-4.51)	0.181
	High	15	41		
CEA	≤5 ng/ml	29	74	1.22 (0.62-2.41)	0.608
	>5 ng/ml	18	56		
CA199	≤37 U/ml	38	105	0.89 (0.37-2.09)	0.824
	>37 U/ml	9	22		

	variables	SRSF3 expression		OR (95%CI)	p value
		low	High		
CA125	≤35 U/ml	38	99	3.07 (0.37-25.39)	0.445
	>35 U/ml	1	8		

Table 3. Correlation between DHCR24 expression and clinicopathological characteristics

	variables	DHCR24		OR (95%CI)	p value
		low	High		
SRSF3	Low	35	72	2.12 (1.23-3.53)	**0.006**
	High	48	209		
Gender	Male	40	134	1.17 (0.68-2.02)	0.584
	Female	28	110		
Age (year)	≤60	25	70	1.43 (0.79-2.60)	0.277
	>60	32	128		
Location	Colon	42	147	1.12 (0.66-1.92)	0.785
	Rectum	28	110		
Tumor size	≤5 cm	44	173	0.84 (0.48-1.47)	0.567
	>5 cm	25	83		
Tumor volume	≤25 cm^3	29	139	0.61 (0.36-1.05)	0.078
	>25 cm^3	40	117		
Adenocarcinoma	Yes	58	238	2.38 (1.07-5.27)	**0.036**
	No	11	19		
T stage	T1/T2	14	51	1.01 (0.52-1.96)	1.000
	T3/T4	55	202		
N stage	N0	40	147	1.00 (0.58-1.71)	1.000
	N1/N2	29	106		
Metastatic lymph nodes	≤2	17	59	1.23 (0.53-2.88)	0.673
	>2	11	47		
M stage	M0	64	237	0.86 (0.31-2.45)	0.785
	M1	5	16		
TNM stage	I/II	40	143	1.06 (0.62-1.82)	0.891
	III/IV	29	110		
Borrmann type	I	23	68	1.18 (0.51-2.76)	0.832
	II	10	35		
	III	17	80	1.03 (0.37-2.86)	1.000
	IV	6	29		
Ki67	Low	12	44	0.99 (0.36-2.71)	1.000
	High	8	29		
P53	Low	8	36	0.69 (0.25-1.87)	0.614
	High	12	37		
MSH2	Low	3	12	0.90 (0.23-3.55)	1.000
	High	17	61		
MSH6	Low	7	30	0.77 (0.28-2.16)	0.797
	High	13	43		
CEA	≤5 ng/ml	23	61	1.37 (0.64-2.94)	0.450
	>5 ng/ml	14	51		
CA199	≤37 U/ml	34	88	8.89 (1.15-68.4)	**0.016**

	variables	DHCR24		OR (95%CI)	p value
		low	High		
CA125	>37 U/ml	1	23	1.86 (0.21-16.54)	1.000
	≤35 U/ml	32	86		
	>35 U/ml	1	5		

Table 4. The pharmacokinetics properties of SFI003 in ICR mice

Parameter	Value	
Dosing Route	i.v.	p.o.
Dose (mg/kg)	10	100
T_{max} (hr)	/	2.00
C_{max} (ng/mL)	/	4436.67
$AUC_{0\text{-}last}$ (ng/mL*hr)	5886.43	30911.46
AUC_{INF} (ng/mL*hr)	5893.24	30919.45
$t_{1/2}$ (hr)	0.40	1.86
MRTINF_obs(hr)	0.44	4.66
CL_obs (L/hr/kg)	1696.86	/
Vss (L/kg)	0.75	/
F (%)	52.51	

Table 5. The combination index of SFI003 and rapamycin in HCT-116 and sw480 cells.

HCT-116				SW480			
SFI003 (μM)	rapamycin (μM)	Fa	CI	SFI003 (μM)	rapamycin (μM)	Fa	CI
1	30	0.473525	0.450	1	15	0.534128	0.793
5	30	0.500591	0.337	5	15	0.554716	0.816
10	30	0.5778	0.619	10	15	0.7035	0.601
20	30	0.6904	0.857	20	15	0.7763	0.533
50	30	0.91719	0.967	50	15	0.8479	0.496
100	30	0.9794	0.952	100	15	0.8719	0.569

Figures

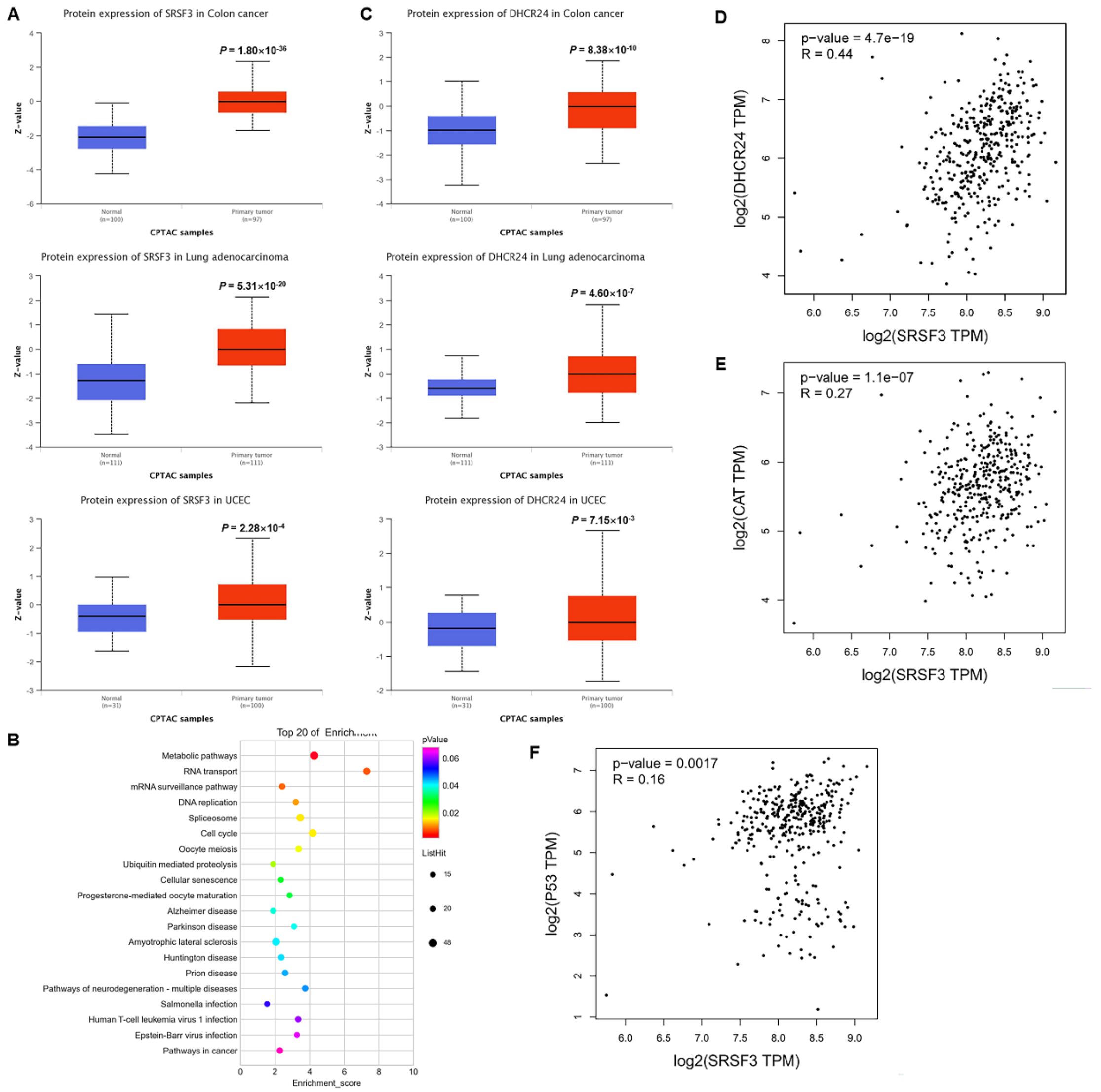

Fig. 1 The expression of SRSF3 and DHCR24. (**A**) The expression of SRSF3 protein in colon, lung, and uterine corpus endometrial carcinoma samples from CPTAC database. (**B**) The pathway enrichment of SRSF3-related genes (r>0.5) in CRC samples from TCGA database. (**C**) The expression of DHCR24 protein in colon, lung, and uterine corpus endometrial carcinoma samples from CPTAC database. (**D**) Positive correlation between SRSF3 and DHCR24 mRNA expression in CRC samples from TCGA database. (**E**) The correlation between SRSF3 and CAT mRNA expression in CRC samples from TCGA database. (**F**) The correlation between SRSF3 and P53 mRNA expression in CRC samples from TCGA database. Data represent mean ± SD. Significance was assessed by two-sided *t*-test.

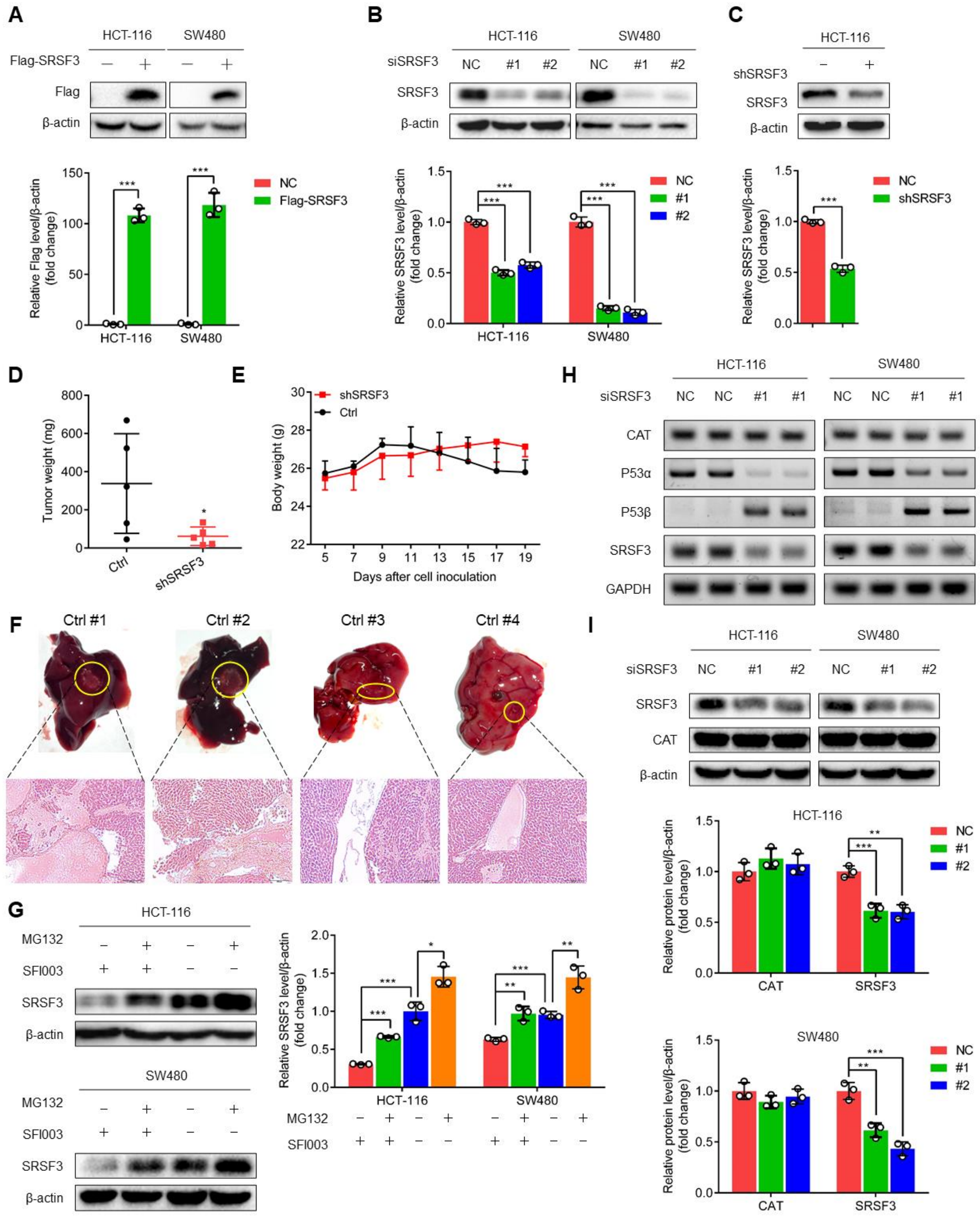

Fig. 2 SRSF3 knockdown suppressed CRC tumor growth and liver damage *in vivo*. (**A**) SRSF3 protein expression in HCT-116 and SW480 cells upon transfection with vector control or Flag-SRSF3 expression vector for 48 h. (**B**) SRSF3 protein expression in HCT-116 and SW480 cells upon transfection with siRNA control or SRSF3 siRNA for 48 h. (**C**) The protein level of SRSF3 in HCT-116-control and HCT-116-shSRSF3 cells. (**D-F**) The tumor weight (D), body weight (E) and H＆E stained livers (F) of tumor-bearing mice. (**G**) The effects of SFI003 and MG132 on the

expression of SRSF3 protein. HCT-116 and SW480 cells were treated with 20 μM SFI003 for 72 h and/or 10 μM MG132 for 12 h. (**H**) The effects of SRSF3 silence on the mRNA expression of CAT, P53α, and P53β. (**I**) The effects of SRSF3 silence on the protein expression of CAT. HCT-116 and SW480 cells were transfected with siRNA control or SRSF3 siRNA for 48 h. Data represent mean ± SD. Significance was assessed by two-sided *t*-test. ***$P < 0.001$; **$P < 0.01$; *$P < 0.05$.

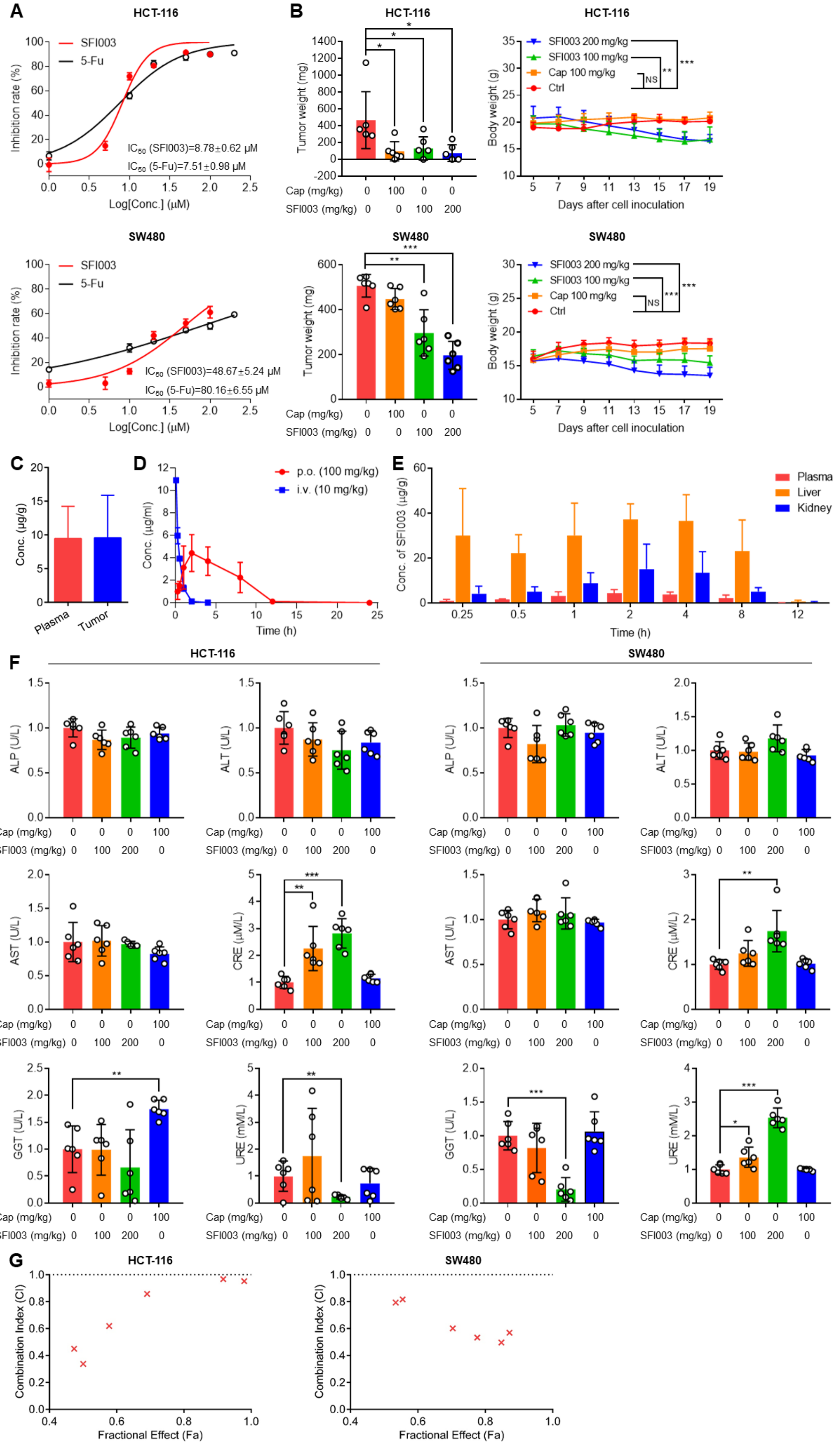
A
HCT-116
SFI003
5-Fu
Inhibition rate (%)
IC50 (SFI003)=8.78±0.62 μM
IC50 (5-Fu)=7.51±0.98 μM
Log[Conc.] (μM)
SW480
IC50 (SFI003)=48.67±5.24 μM
IC50 (5-Fu)=80.16±6.55 μM
B
Tumor weight (mg)
Cap (mg/kg)
SFI003 (mg/kg)
Body weight (g)
SFI003 200 mg/kg
SFI003 100 mg/kg
Cap 100 mg/kg
Ctrl
NS
Days after cell inoculation
C
Conc. (μg/g)
Plasma
Tumor
D
Conc. (μg/ml)
p.o. (100 mg/kg)
i.v. (10 mg/kg)
Time (h)
E
Conc. of SFI003 (μg/g)
Plasma
Liver
Kidney
Time (h)
F
HCT-116
SW480
ALP (U/L)
ALT (U/L)
AST (U/L)
CRE (μM/L)
GGT (U/L)
URE (mM/L)
G
HCT-116
SW480
Combination Index (CI)
Fractional Effect (Fa)

Fig. 3 The activities and safety of SFI003 against CRC. (**A**) The inhibition curves of SFI003 and 5-Fu in HCT-116 and SW480 cells, which were treated with indicated doses of 5-Fu or SFI003 for 72 h. (**B**) The tumor weight and body weight of the HCT-116- or SW480-tumor bearing nude mice upon treatment with SFI003 or Capecitabine. (**C**) The concentrations of SFI003 in the plasma and tumors of nude mice grafted with SW480 cells after treatment with 100 mg/kg SFI003 for 2 h. (**D**) The plasma concentration-time curve of SFI003 in ICR mice. (**E**) The distribution of SFI003 in the plasma, liver, and kidney of ICR mice treated at 100 mg/kg SFI003. (**F**) The biochemical analysis of the serum in the HCT-116- or SW480-tumor bearing nude mice after treatment with SFI003 or Capecitabine. (**G**) Fractional Effect-Combination Index Plot of SFI003 combined with rapamycin in HCT-116 and SW480 cells. Data represent mean ± SD. Significance was assessed by two-sided *t*-test. ***$P < 0.001$; **$P < 0.01$; *$P < 0.05$.

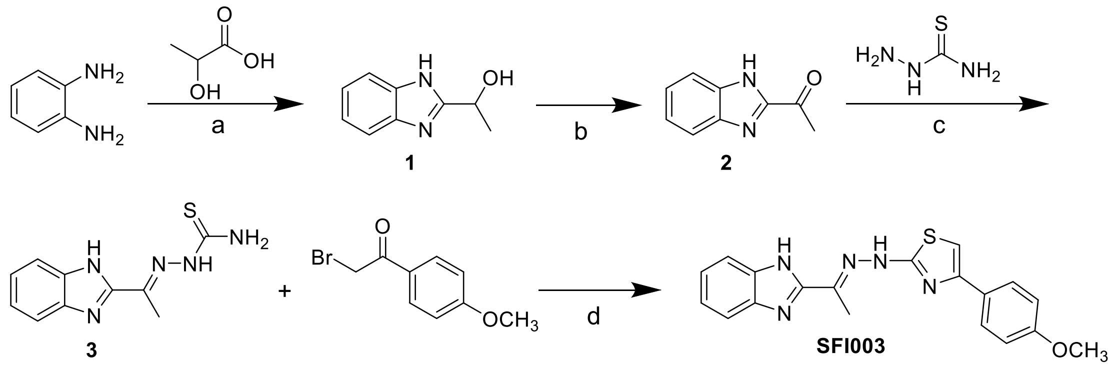

Fig.4 The chemically synthesis process of SFI003. (a) 4M HCl, 90 °C, 1.5 h; (b) CrO_3, AcOH, 105 °C, 0.5 h; (c) EtOH, AcOH, 80 °C, 6 h; (d) EtOH, AcOH, room temperature, 0.5 h.
