# Supplementary material for: A novel SRSF3 inhibitor, SFI003, exerts anticancer activity against colorectal cancer by modulating the SRSF3/DHCR24/ROS axis
Source: Cell Death Discov. 2022 May 2;8:238. doi: 10.1038/s41420-022-01039-9 (PMC9061822; doi:10.1038/s41420-022-01039-9)
Supplement: Supplementary file 2 — Original western blots [file 41420_2022_1039_MOESM2_ESM.pptx]

## Slide 1
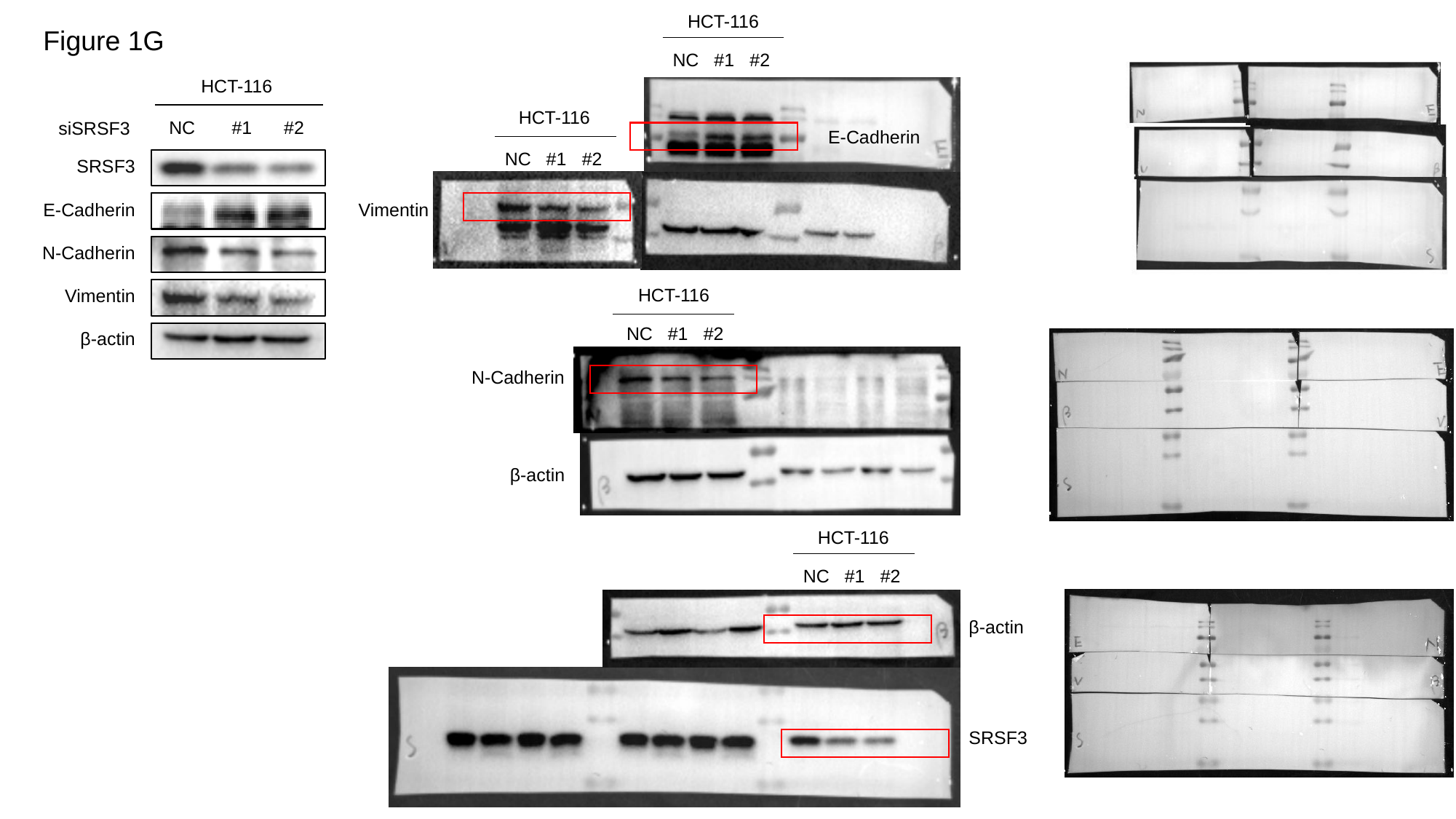

HCT-116
Figure 1G
NC #1 #2
HCT-116
HCT-116
| NC | #1 | #2 |
| --- | --- | --- |
| siSRSF3 |
| --- |
| SRSF3 |
| E-Cadherin |
| N-Cadherin |
| Vimentin |
| β-actin |
E-Cadherin
NC #1 #2
Vimentin
HCT-116
NC #1 #2
N-Cadherin
β-actin
HCT-116
NC #1 #2
β-actin
SRSF3

## Slide 2
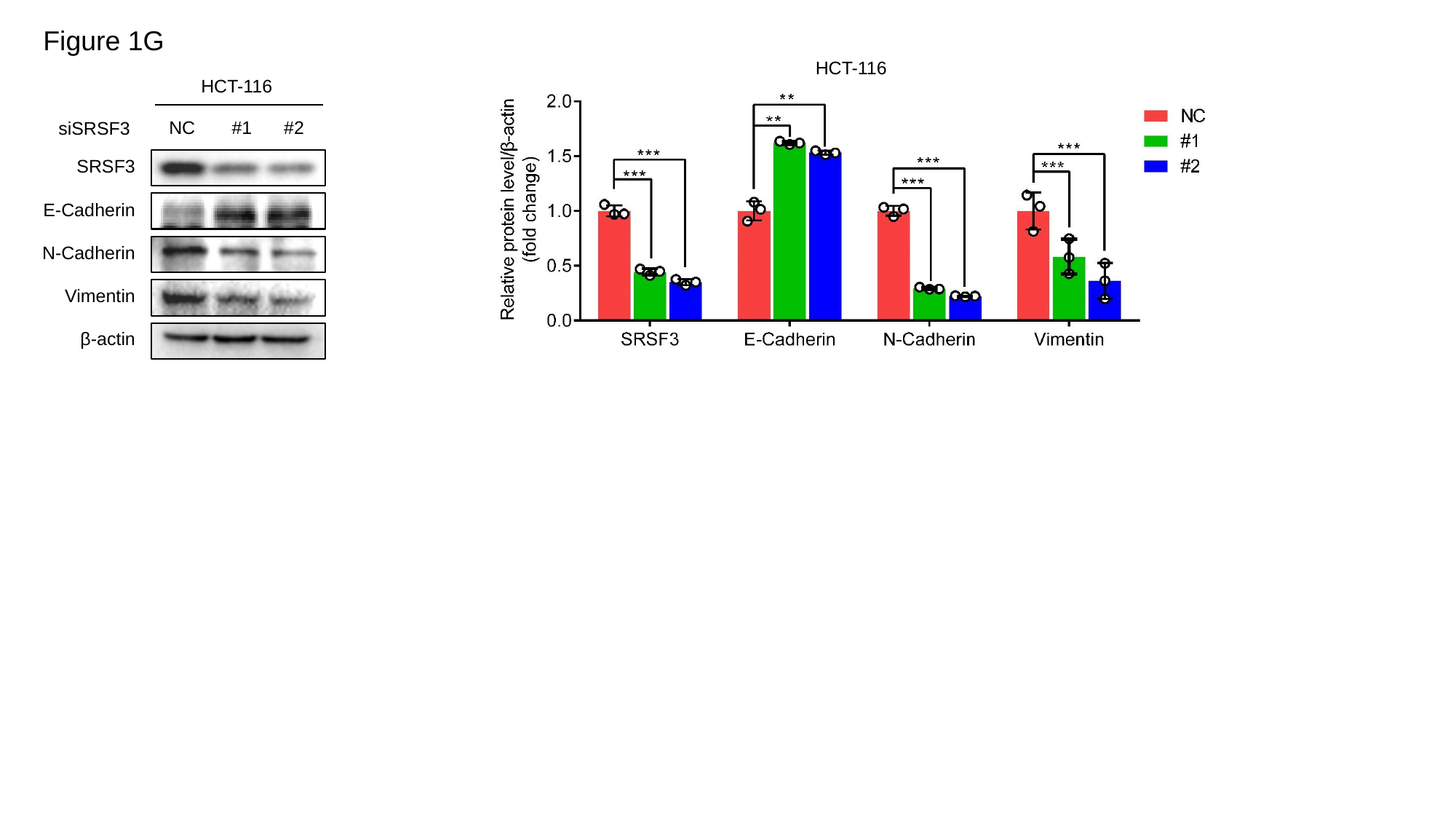

Figure 1G
HCT-116
HCT-116
| NC | #1 | #2 |
| --- | --- | --- |
| siSRSF3 |
| --- |
| SRSF3 |
| E-Cadherin |
| N-Cadherin |
| Vimentin |
| β-actin |

## Slide 3
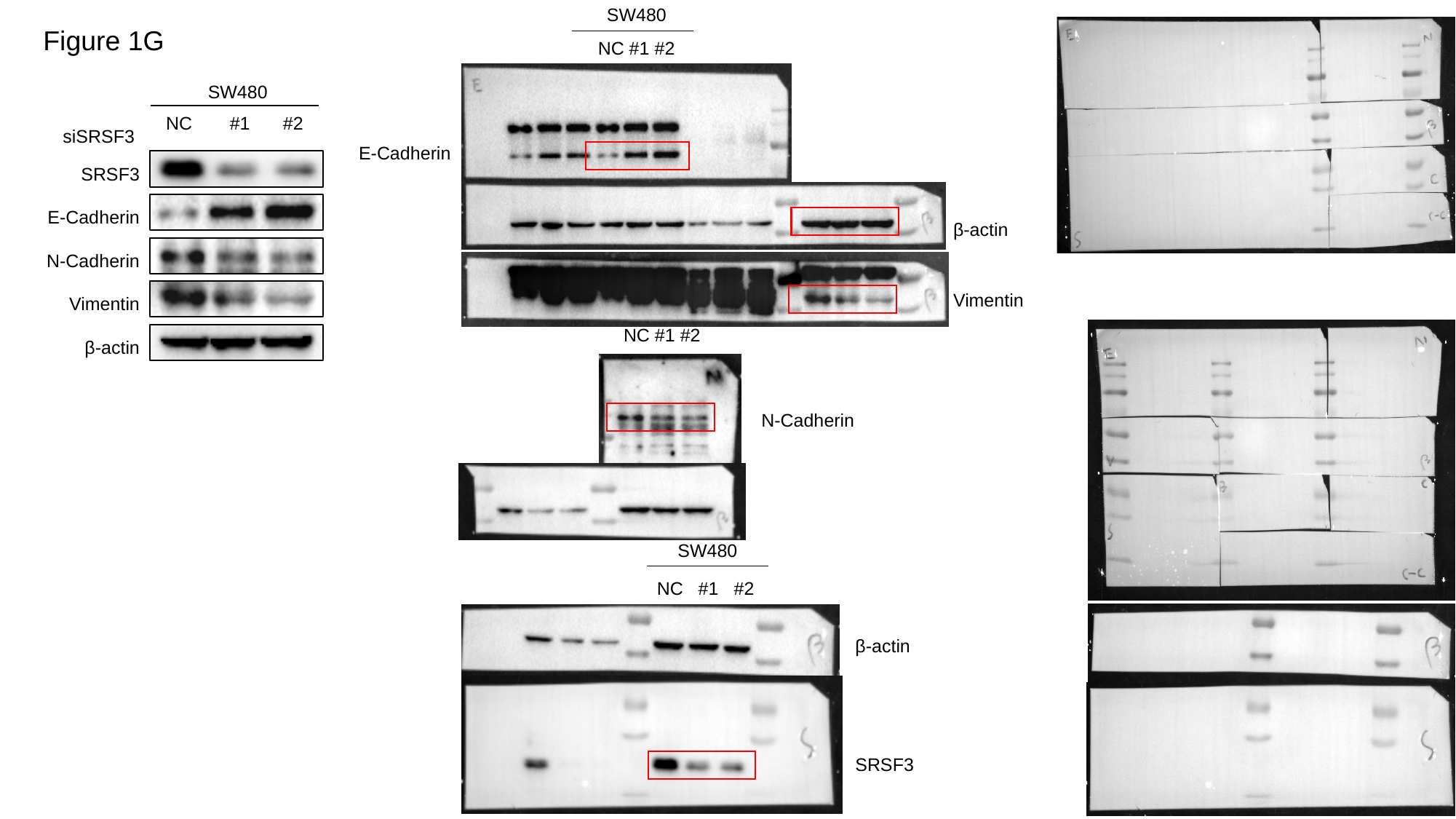

SW480
Figure 1G
NC #1 #2
SW480
| NC | #1 | #2 |
| --- | --- | --- |
| siSRSF3 |
| --- |
| SRSF3 |
| E-Cadherin |
| N-Cadherin |
| Vimentin |
| β-actin |
E-Cadherin
β-actin
Vimentin
NC #1 #2
N-Cadherin
SW480
NC #1 #2
β-actin
SRSF3

## Slide 4
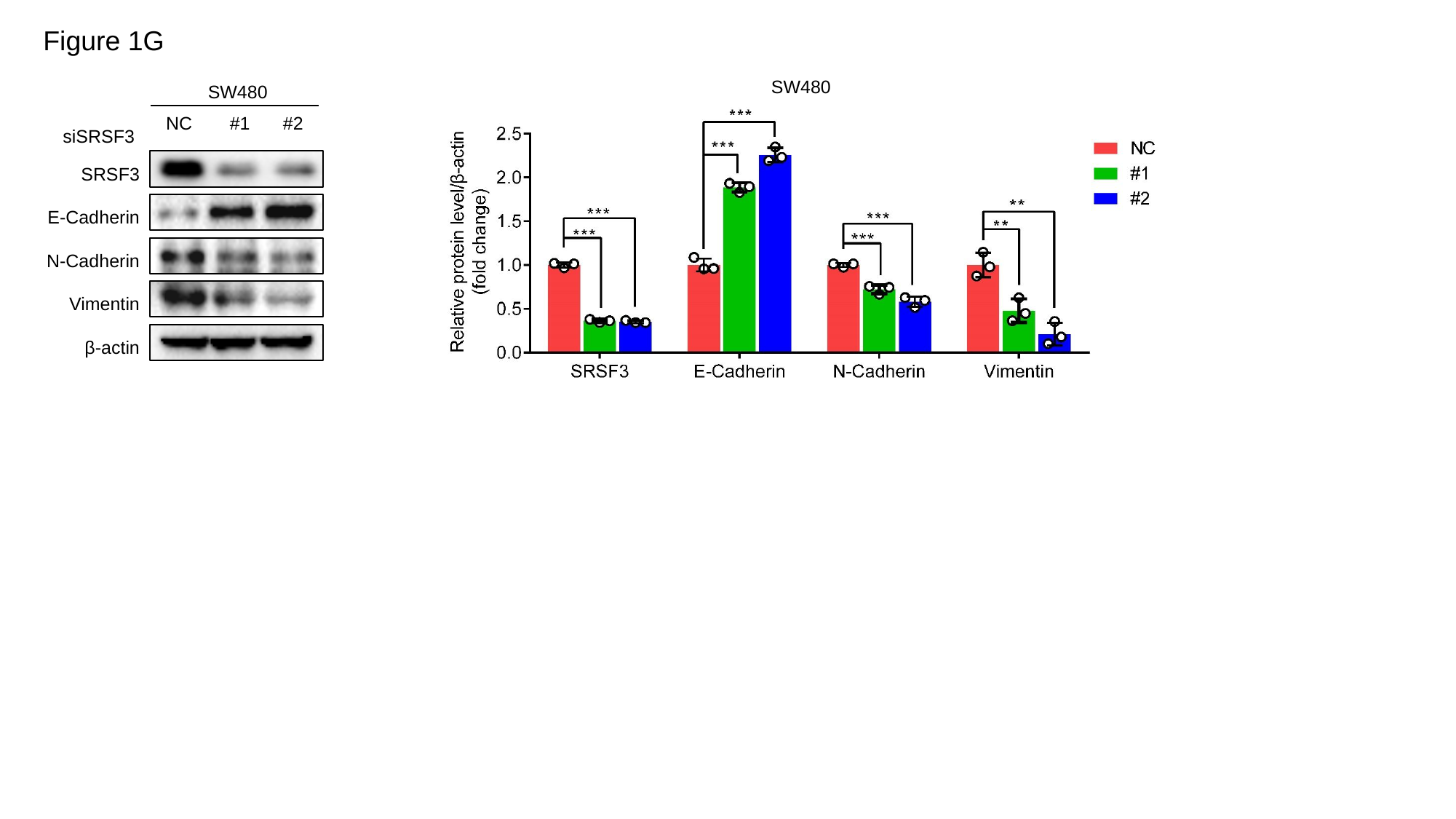

Figure 1G
SW480
SW480
| NC | #1 | #2 |
| --- | --- | --- |
| siSRSF3 |
| --- |
| SRSF3 |
| E-Cadherin |
| N-Cadherin |
| Vimentin |
| β-actin |

## Slide 5
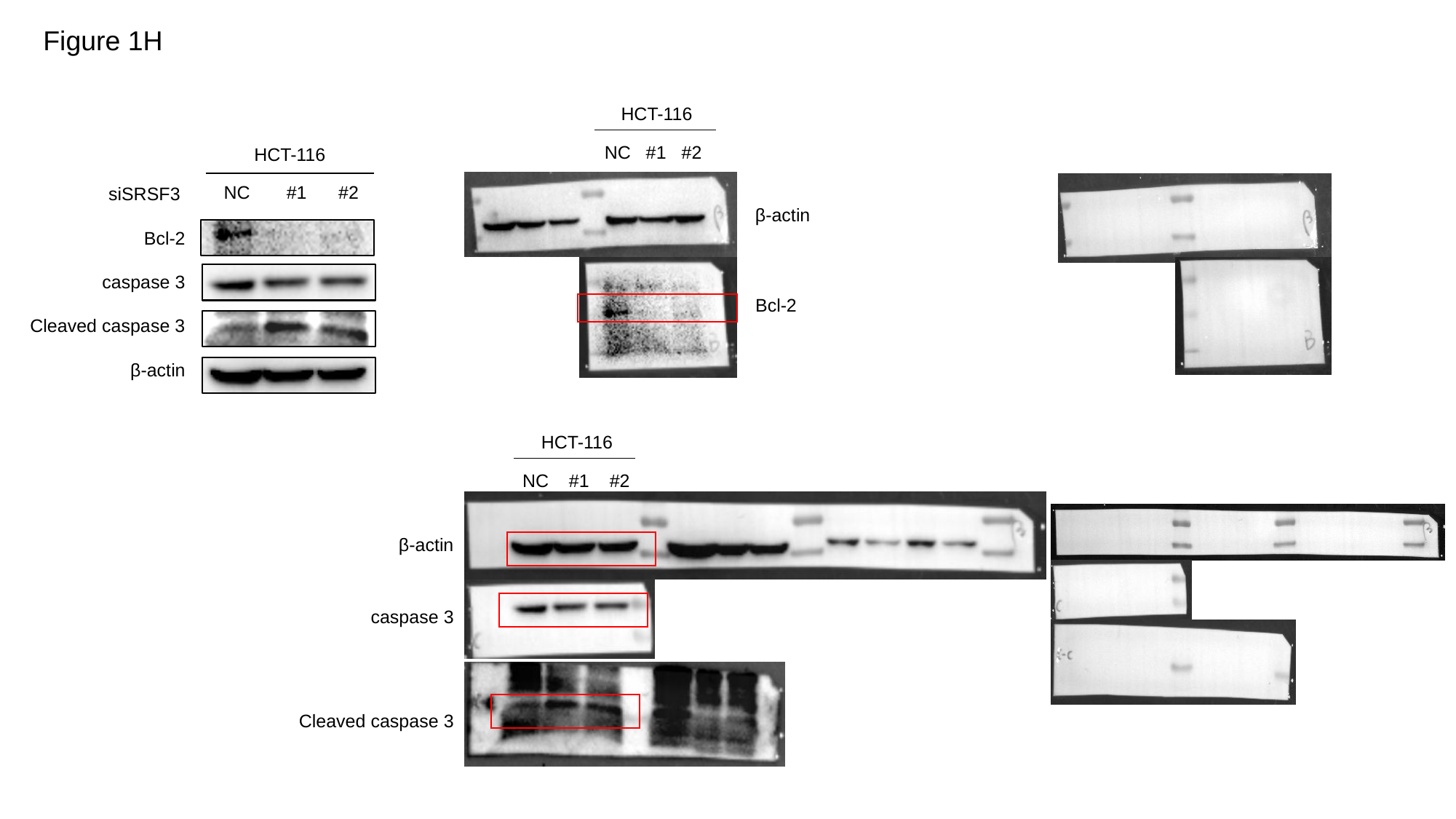

Figure 1H
HCT-116
NC #1 #2
HCT-116
| NC | #1 | #2 |
| --- | --- | --- |
| siSRSF3 |
| --- |
| Bcl-2 |
| caspase 3 |
| Cleaved caspase 3 |
| β-actin |
β-actin
Bcl-2
HCT-116
NC #1 #2
β-actin
caspase 3
Cleaved caspase 3

## Slide 6
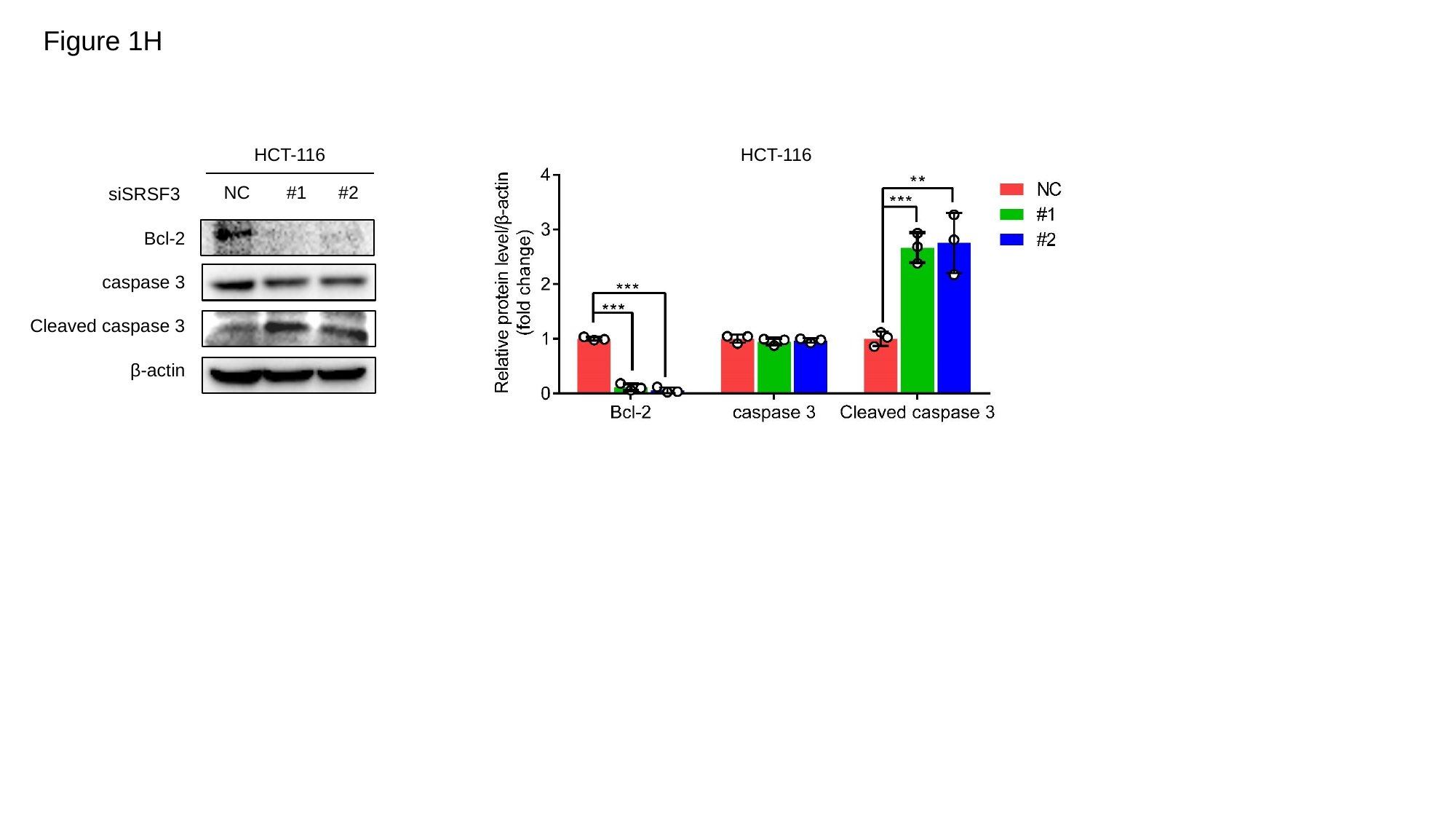

Figure 1H
HCT-116
HCT-116
| NC | #1 | #2 |
| --- | --- | --- |
| siSRSF3 |
| --- |
| Bcl-2 |
| caspase 3 |
| Cleaved caspase 3 |
| β-actin |

## Slide 7
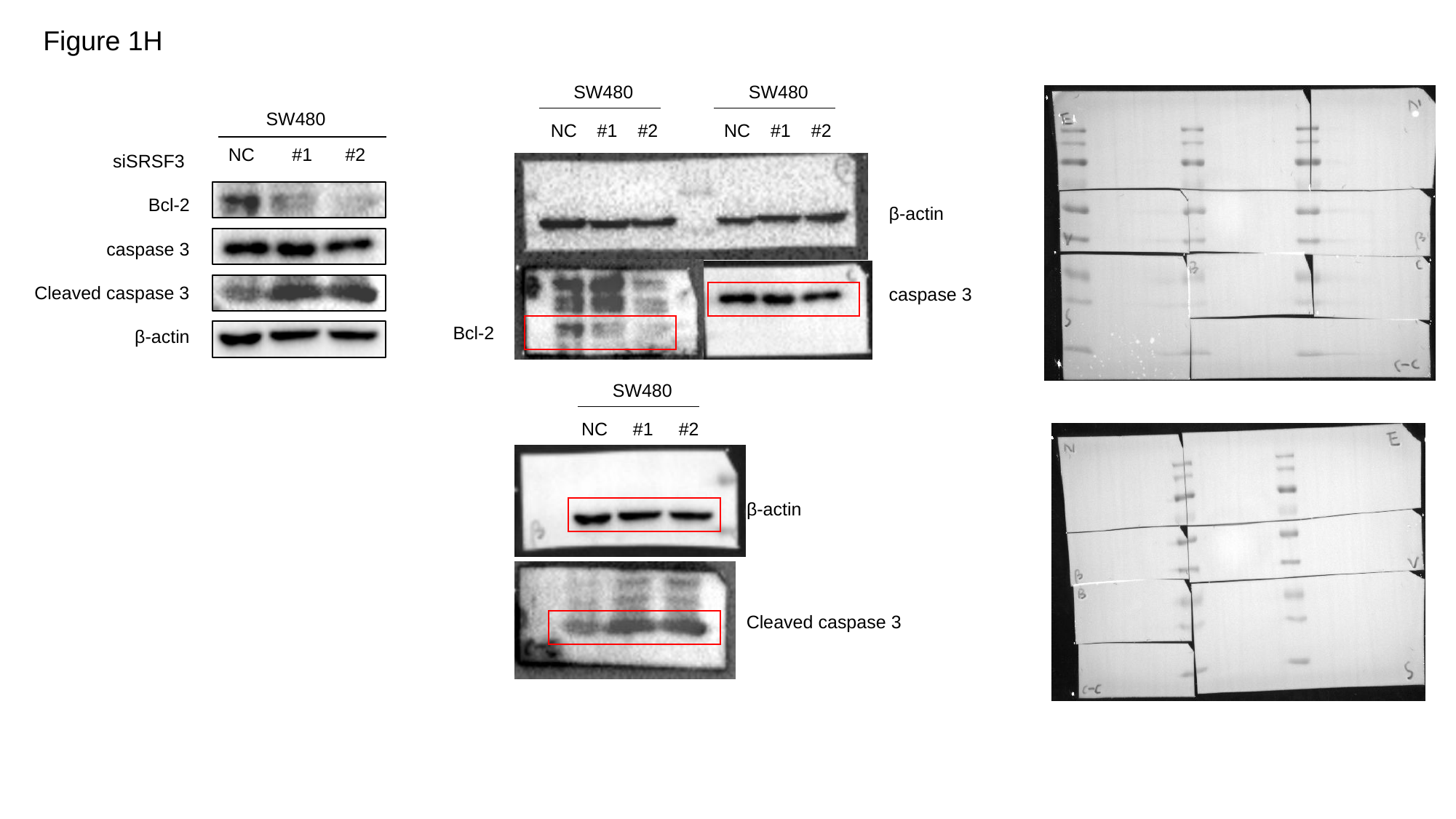

Figure 1H
SW480
SW480
SW480
NC #1 #2
NC #1 #2
| NC | #1 | #2 |
| --- | --- | --- |
| siSRSF3 |
| --- |
| Bcl-2 |
| caspase 3 |
| Cleaved caspase 3 |
| β-actin |
β-actin
caspase 3
Bcl-2
SW480
NC #1 #2
β-actin
Cleaved caspase 3

## Slide 8
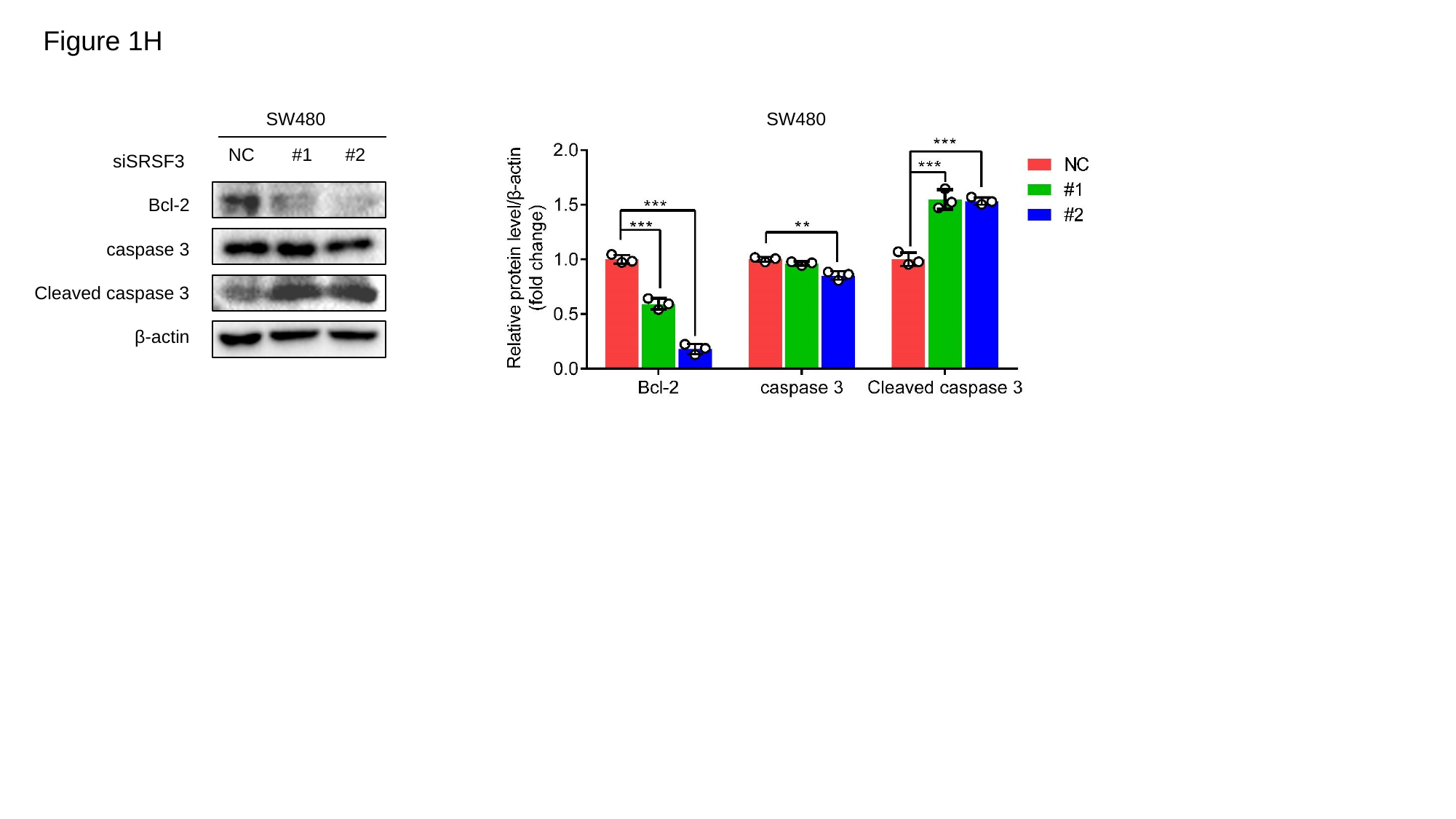

Figure 1H
SW480
SW480
| NC | #1 | #2 |
| --- | --- | --- |
| siSRSF3 |
| --- |
| Bcl-2 |
| caspase 3 |
| Cleaved caspase 3 |
| β-actin |

## Slide 9
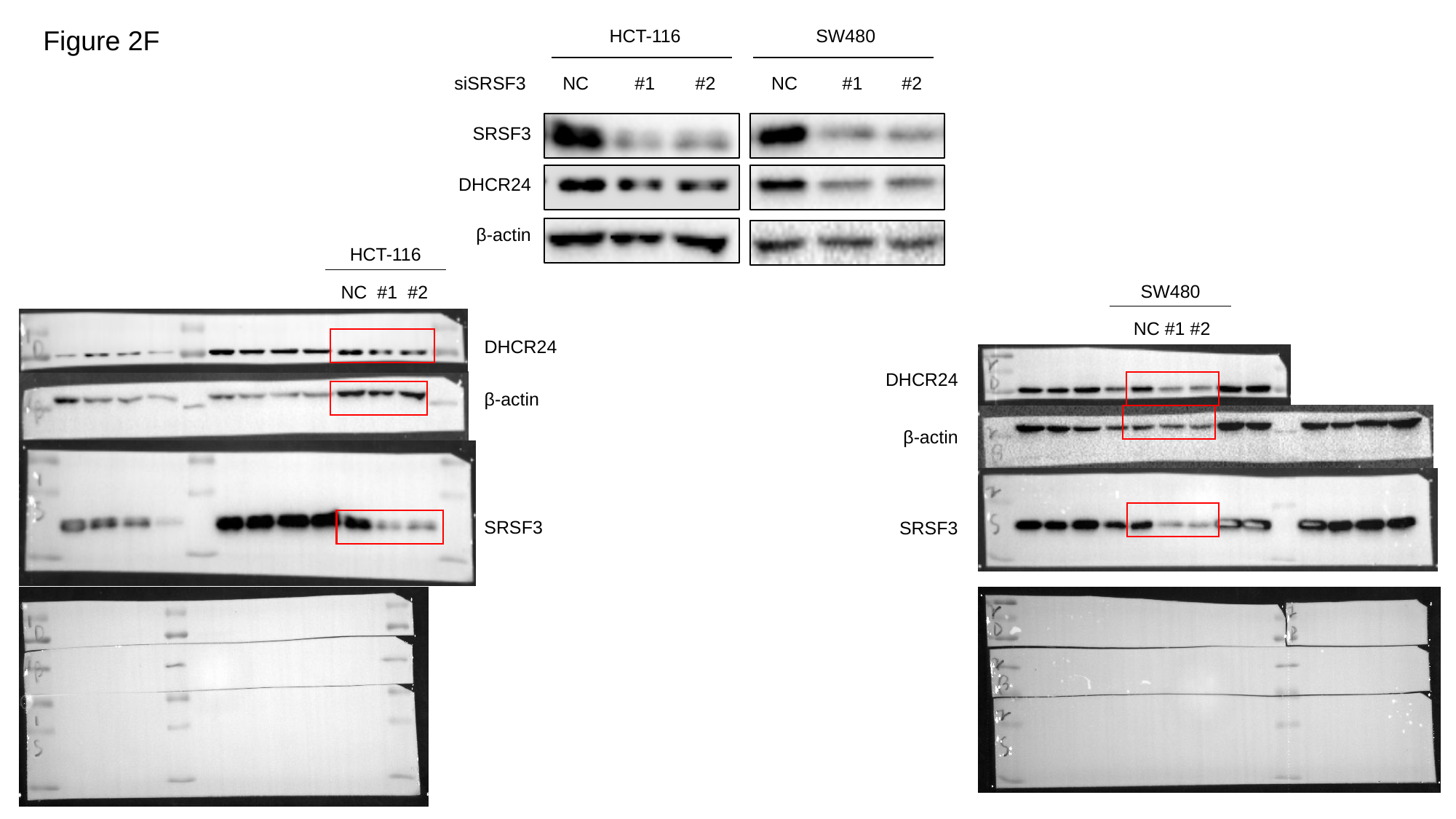

Figure 2F
HCT-116
SW480
| siSRSF3 |
| --- |
| SRSF3 |
| DHCR24 |
| β-actin |
| NC | #1 | #2 |
| --- | --- | --- |
| NC | #1 | #2 |
| --- | --- | --- |
HCT-116
SW480
NC #1 #2
NC #1 #2
DHCR24
DHCR24
β-actin
β-actin
SRSF3
SRSF3

## Slide 10
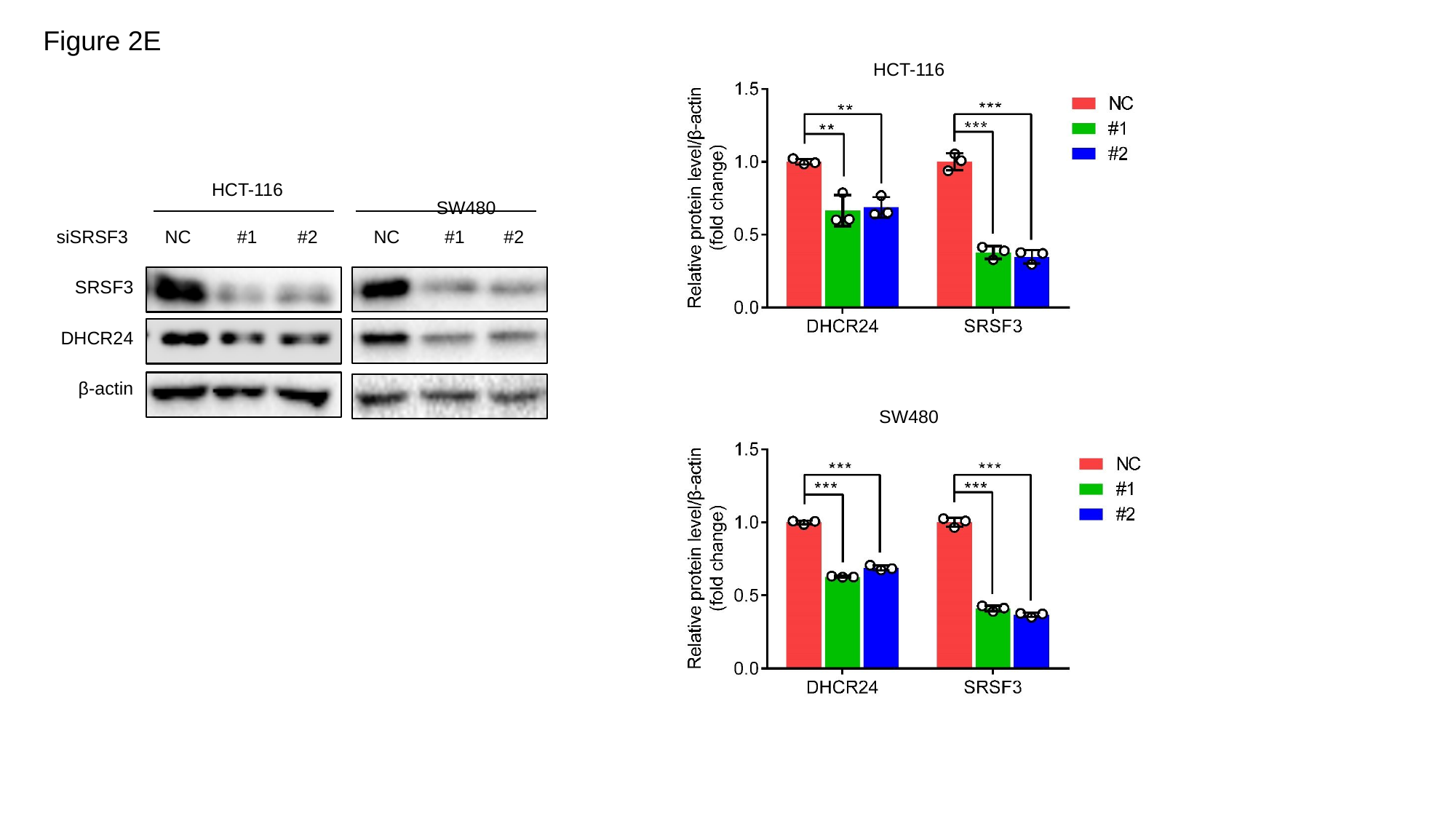

Figure 2E
HCT-116
HCT-116
SW480
| siSRSF3 |
| --- |
| SRSF3 |
| DHCR24 |
| β-actin |
| NC | #1 | #2 |
| --- | --- | --- |
| NC | #1 | #2 |
| --- | --- | --- |
SW480

## Slide 11
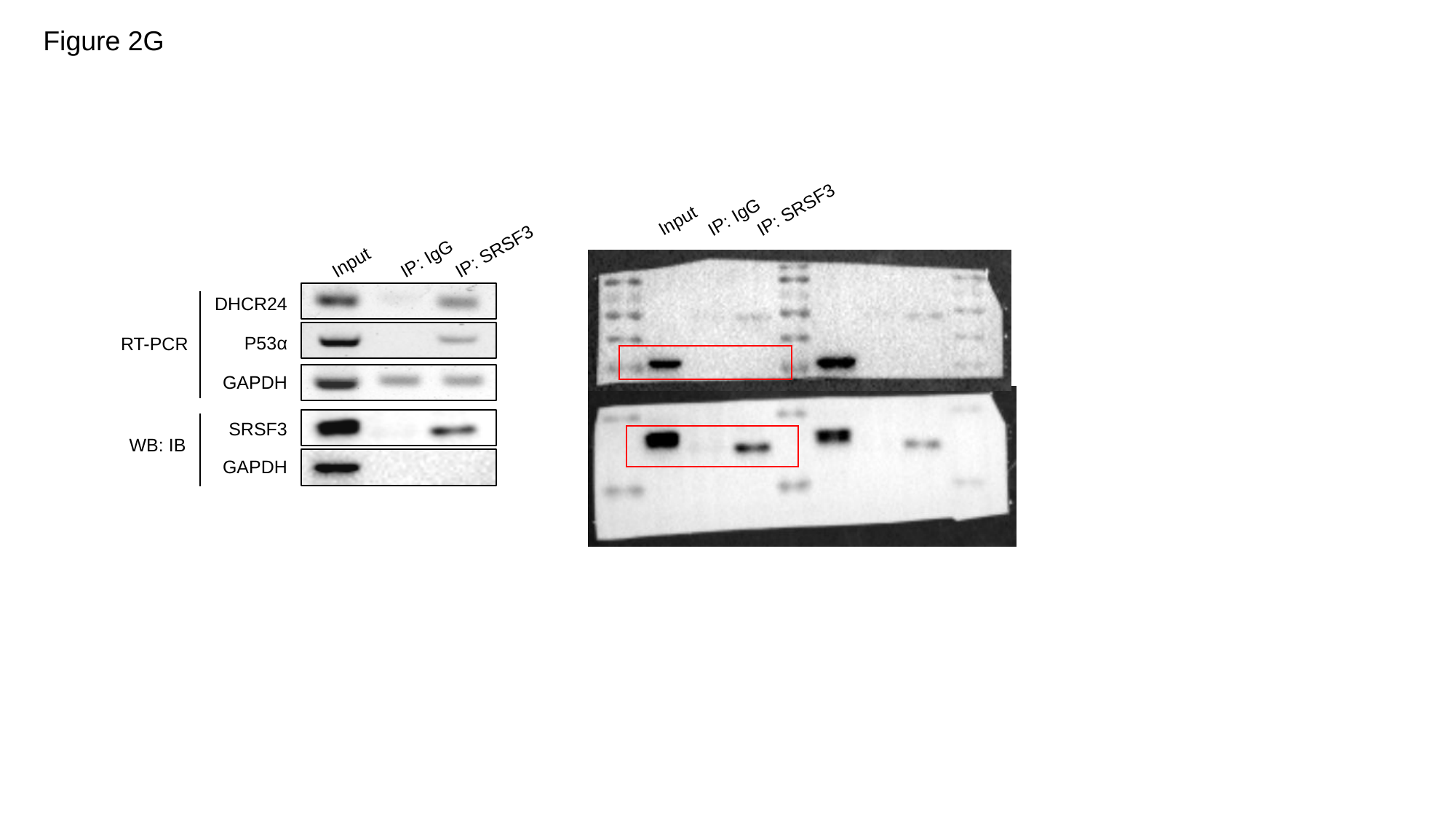

Figure 2G
IP: SRSF3
IP: IgG
Input
IP: SRSF3
IP: IgG
Input
| DHCR24 |
| --- |
| P53α |
| GAPDH |
RT-PCR
| SRSF3 |
| --- |
| GAPDH |
WB: IB

## Slide 12
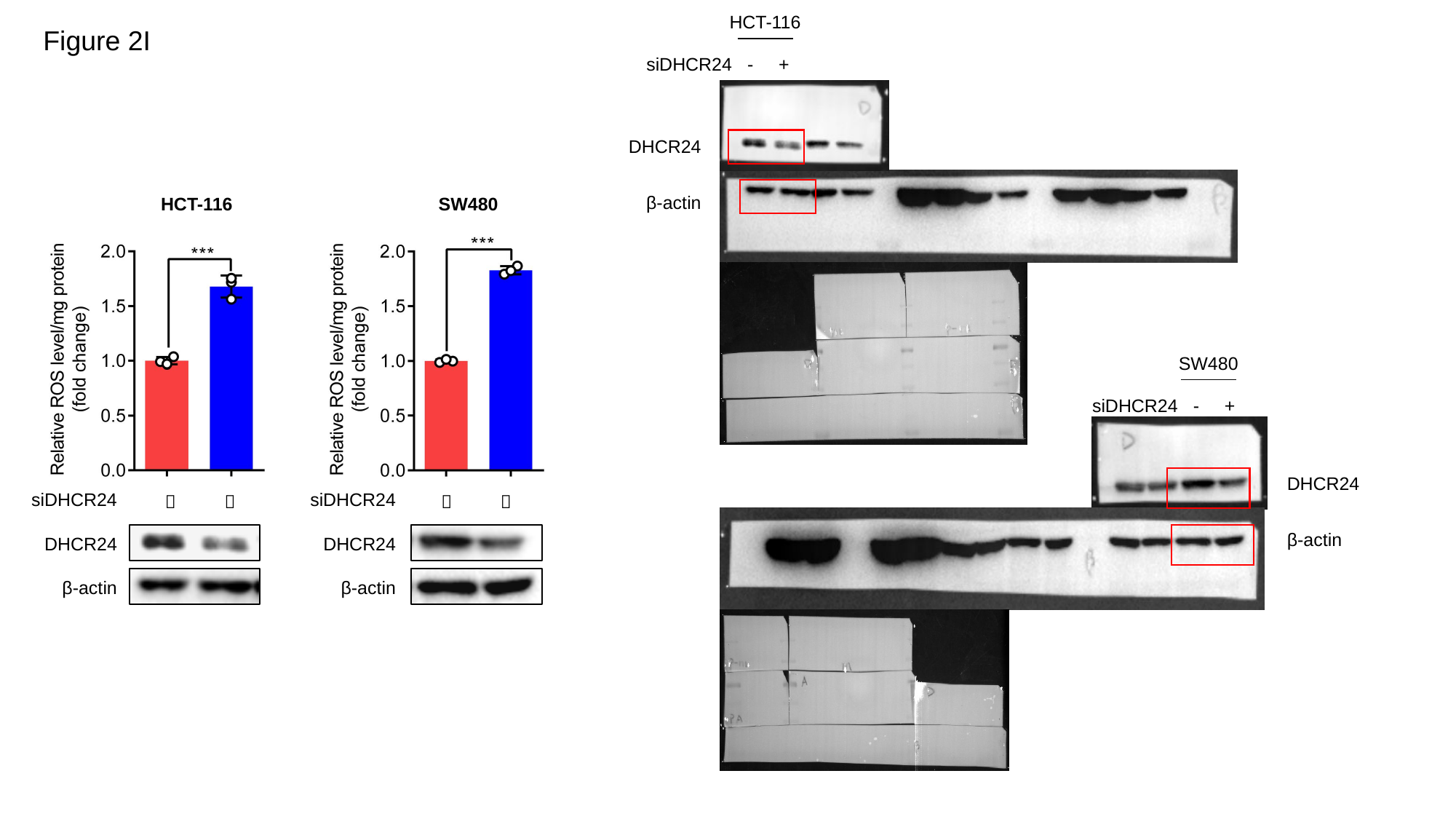

HCT-116
Figure 2I
siDHCR24 - +
DHCR24
β-actin
HCT-116
SW480
SW480
siDHCR24 - +
DHCR24
| － | ＋ |
| --- | --- |
| － | ＋ |
| --- | --- |
| siDHCR24 |
| --- |
| DHCR24 |
| β-actin |
| siDHCR24 |
| --- |
| DHCR24 |
| β-actin |
β-actin

## Slide 13
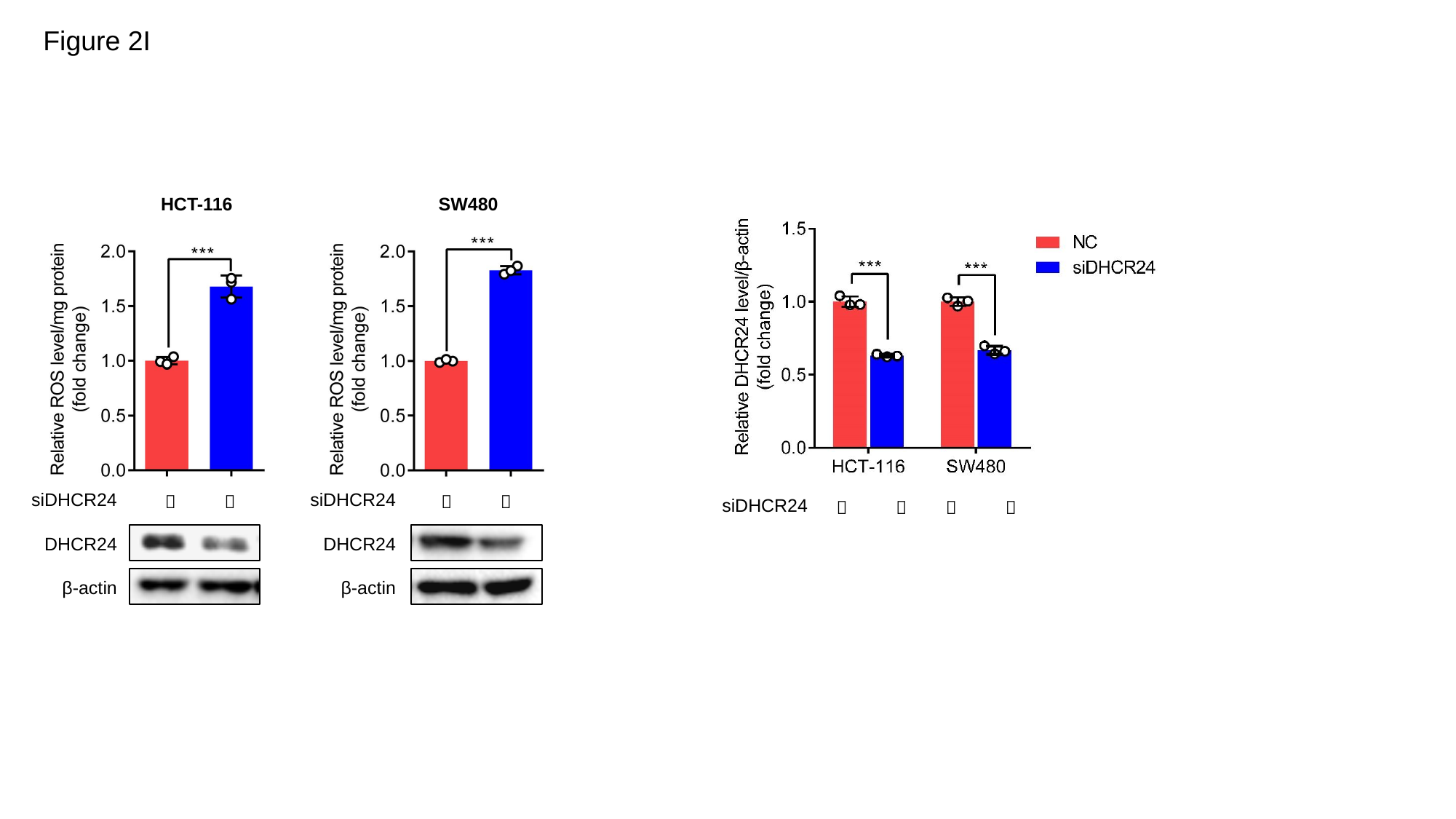

Figure 2I
HCT-116
SW480
| － | ＋ |
| --- | --- |
| － | ＋ |
| --- | --- |
| siDHCR24 |
| --- |
| DHCR24 |
| β-actin |
| siDHCR24 |
| --- |
| DHCR24 |
| β-actin |
siDHCR24
| － | ＋ |
| --- | --- |
| － | ＋ |
| --- | --- |

## Slide 14
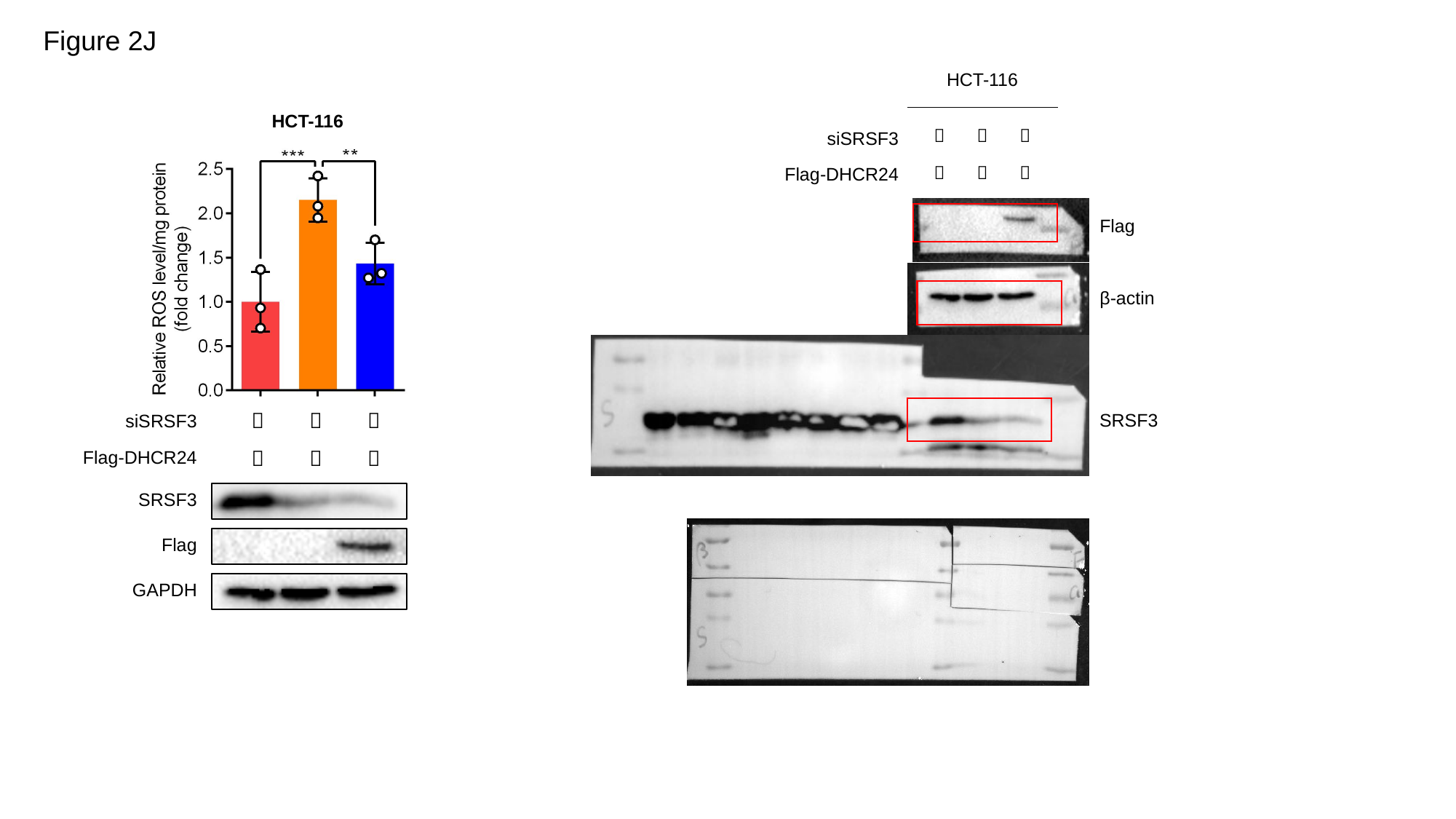

Figure 2J
HCT-116
HCT-116
| － | ＋ | ＋ |
| --- | --- | --- |
| － | － | ＋ |
| siSRSF3 |
| --- |
| Flag-DHCR24 |
Flag
β-actin
| － | ＋ | ＋ |
| --- | --- | --- |
| － | － | ＋ |
SRSF3
| siSRSF3 |
| --- |
| Flag-DHCR24 |
| SRSF3 |
| --- |
| Flag |
| GAPDH |

## Slide 15
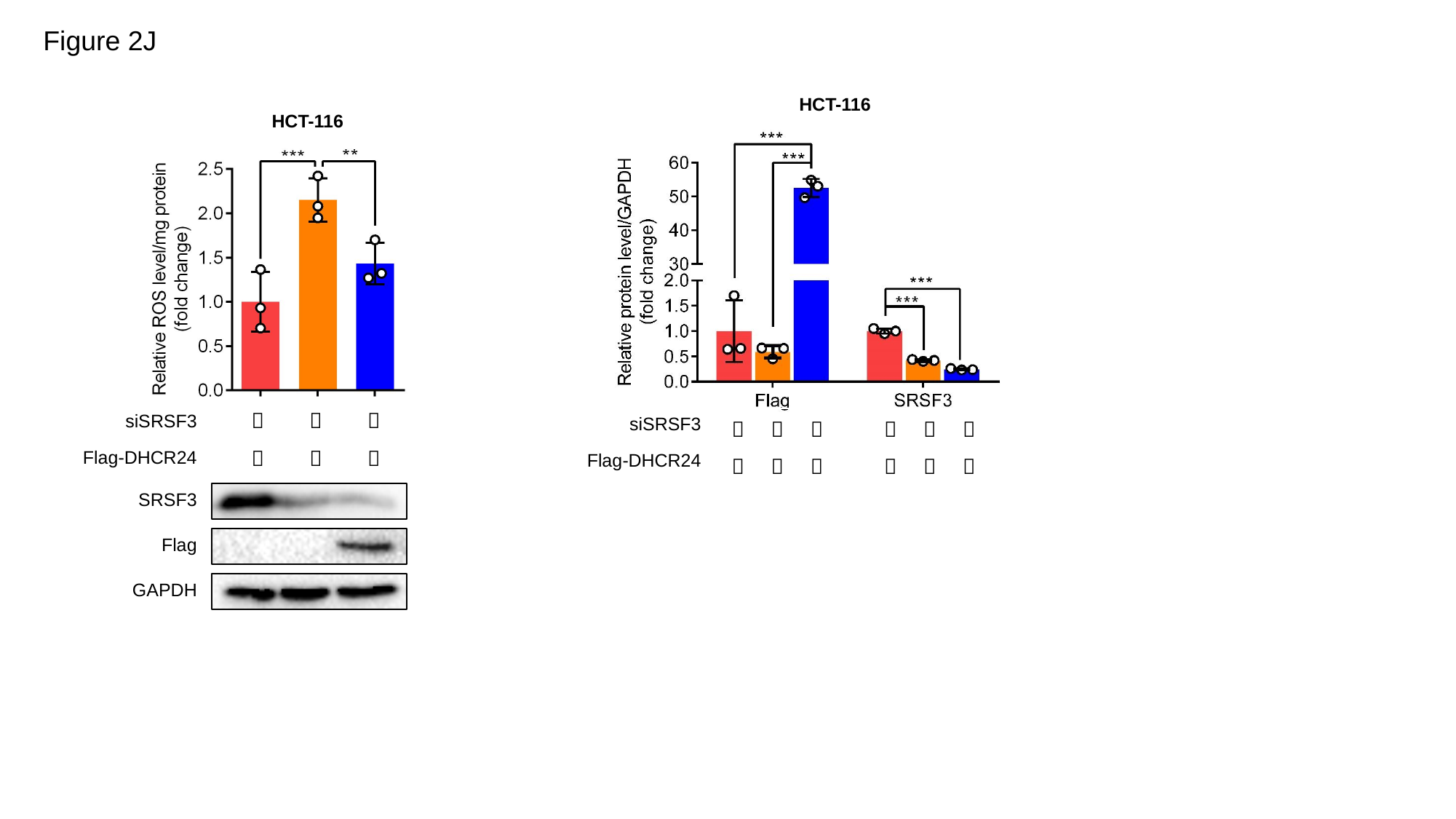

Figure 2J
HCT-116
HCT-116
| － | ＋ | ＋ |
| --- | --- | --- |
| － | － | ＋ |
| siSRSF3 |
| --- |
| Flag-DHCR24 |
| siSRSF3 |
| --- |
| Flag-DHCR24 |
| － | ＋ | ＋ |
| --- | --- | --- |
| － | － | ＋ |
| － | ＋ | ＋ |
| --- | --- | --- |
| － | － | ＋ |
| SRSF3 |
| --- |
| Flag |
| GAPDH |

## Slide 16
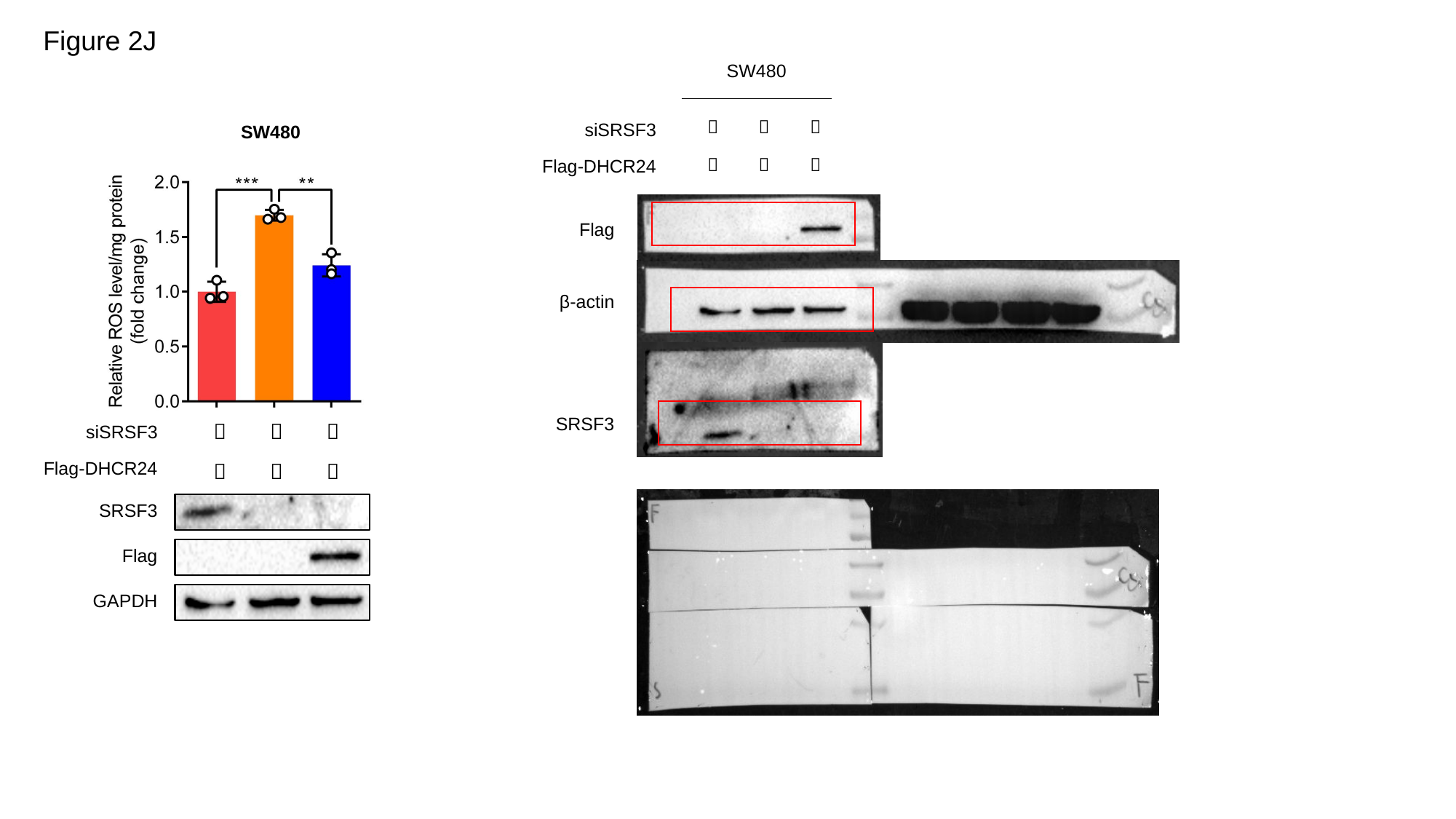

Figure 2J
SW480
| － | ＋ | ＋ |
| --- | --- | --- |
| － | － | ＋ |
| siSRSF3 |
| --- |
| Flag-DHCR24 |
SW480
Flag
β-actin
SRSF3
| － | ＋ | ＋ |
| --- | --- | --- |
| － | － | ＋ |
| siSRSF3 |
| --- |
| Flag-DHCR24 |
| SRSF3 |
| --- |
| Flag |
| GAPDH |

## Slide 17
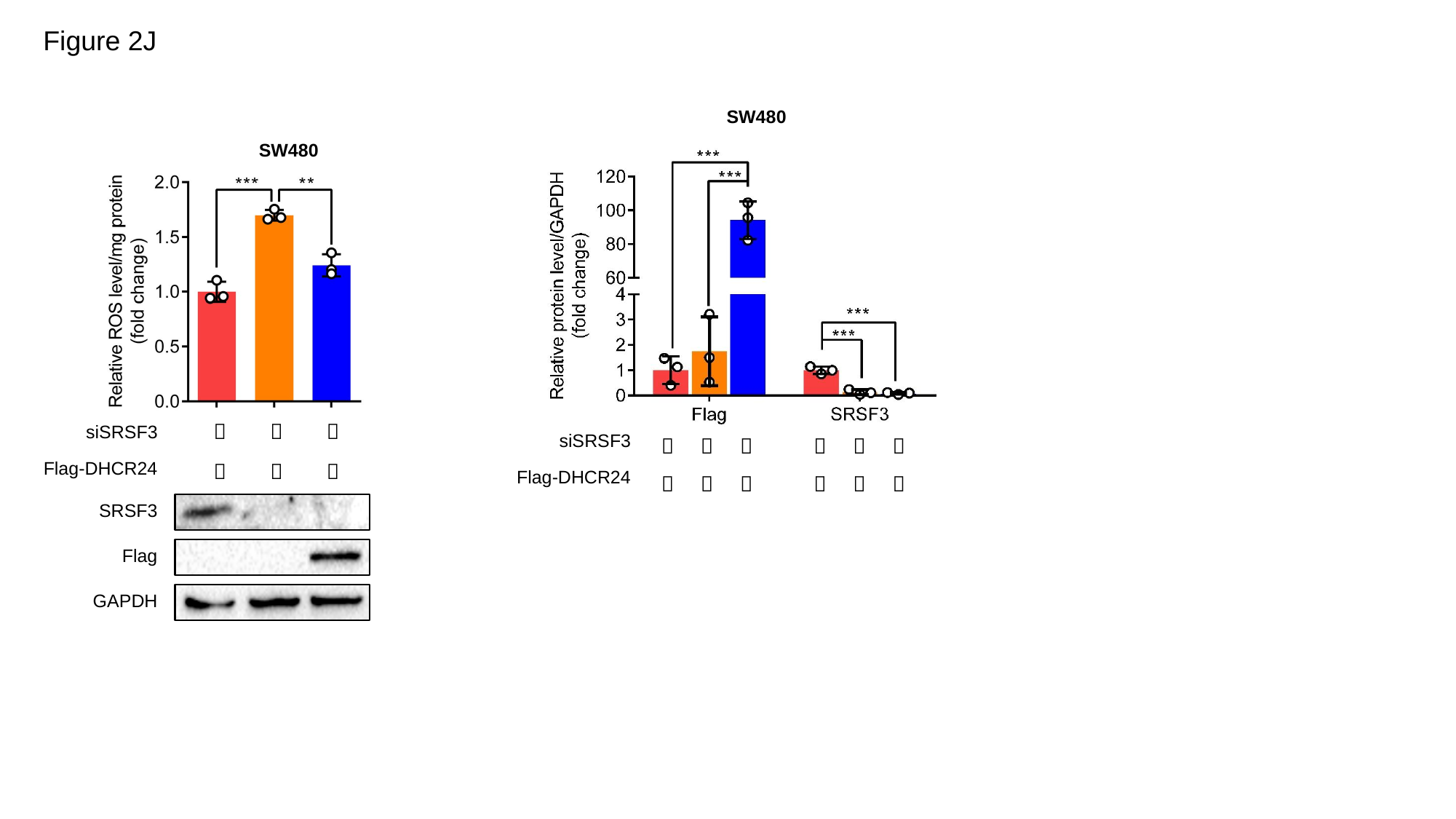

Figure 2J
SW480
SW480
| － | ＋ | ＋ |
| --- | --- | --- |
| － | － | ＋ |
| siSRSF3 |
| --- |
| Flag-DHCR24 |
| siSRSF3 |
| --- |
| Flag-DHCR24 |
| － | ＋ | ＋ |
| --- | --- | --- |
| － | － | ＋ |
| － | ＋ | ＋ |
| --- | --- | --- |
| － | － | ＋ |
| SRSF3 |
| --- |
| Flag |
| GAPDH |

## Slide 18
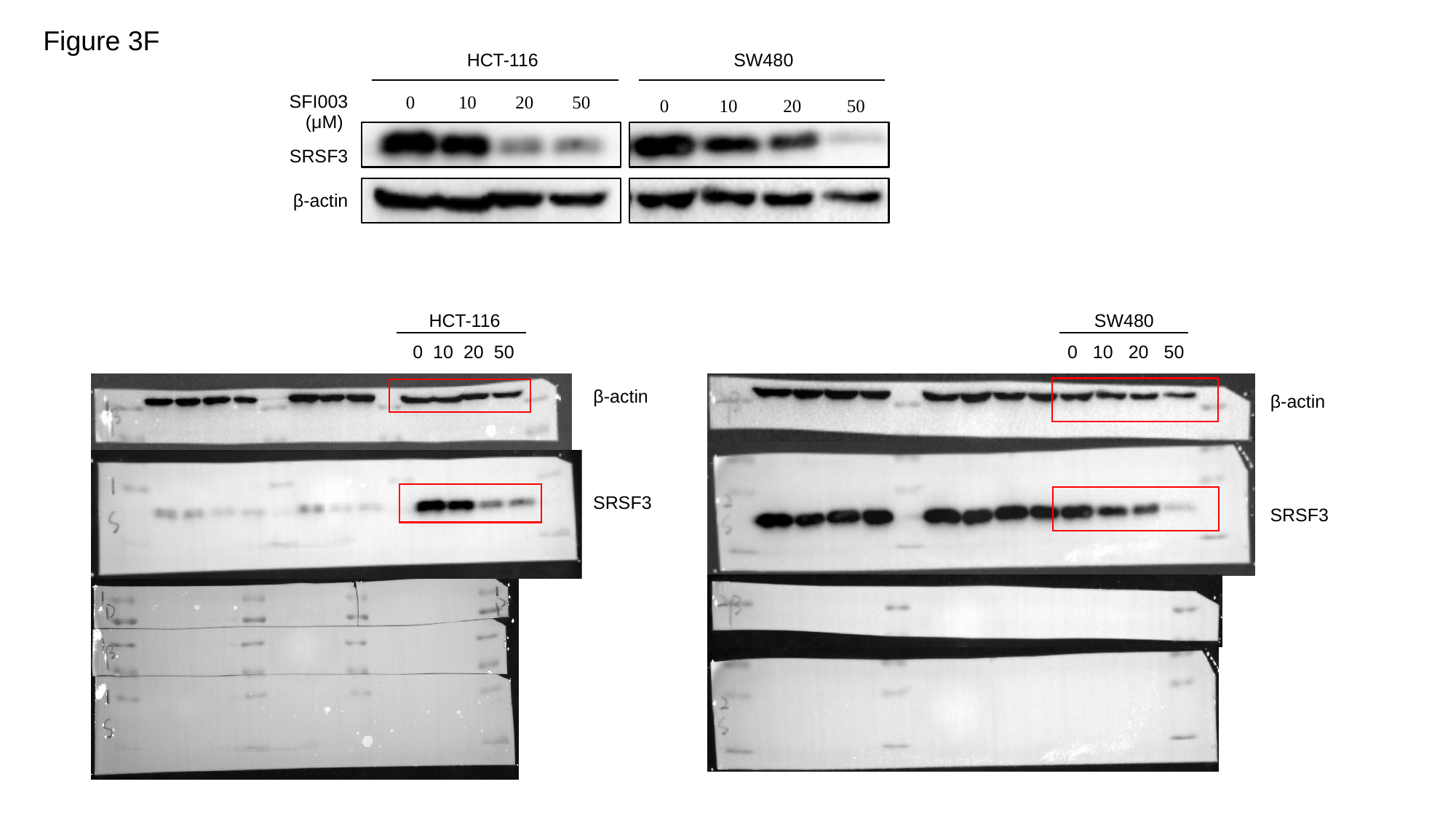

Figure 3F
HCT-116
SW480
| SFI003 (μM) |
| --- |
| SRSF3 |
| β-actin |
| 0 | 10 | 20 | 50 |
| --- | --- | --- | --- |
| 0 | 10 | 20 | 50 |
| --- | --- | --- | --- |
HCT-116
SW480
0 10 20 50
0 10 20 50
β-actin
β-actin
SRSF3
SRSF3

## Slide 19
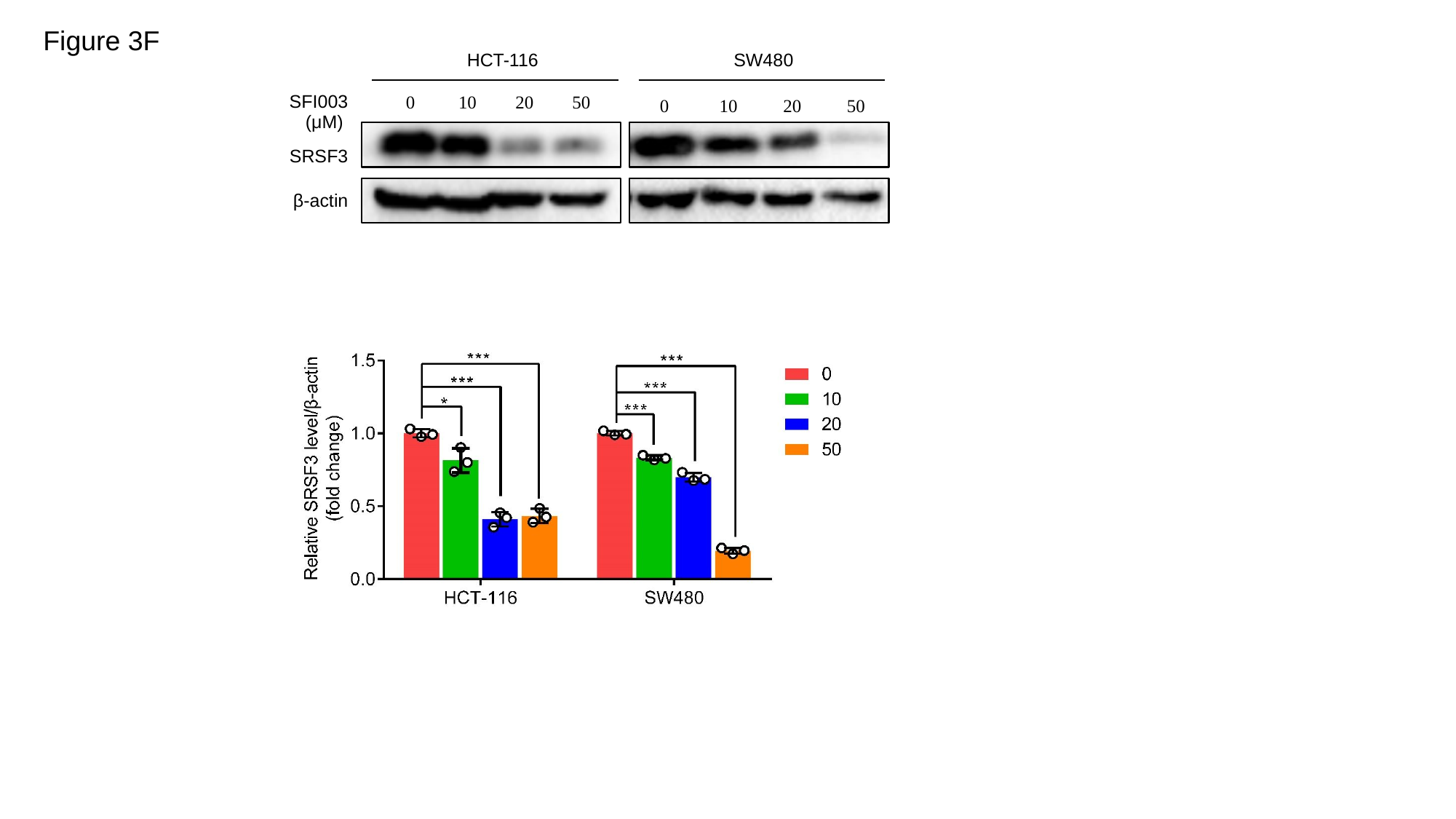

Figure 3F
HCT-116
SW480
| SFI003 (μM) |
| --- |
| SRSF3 |
| β-actin |
| 0 | 10 | 20 | 50 |
| --- | --- | --- | --- |
| 0 | 10 | 20 | 50 |
| --- | --- | --- | --- |

## Slide 20
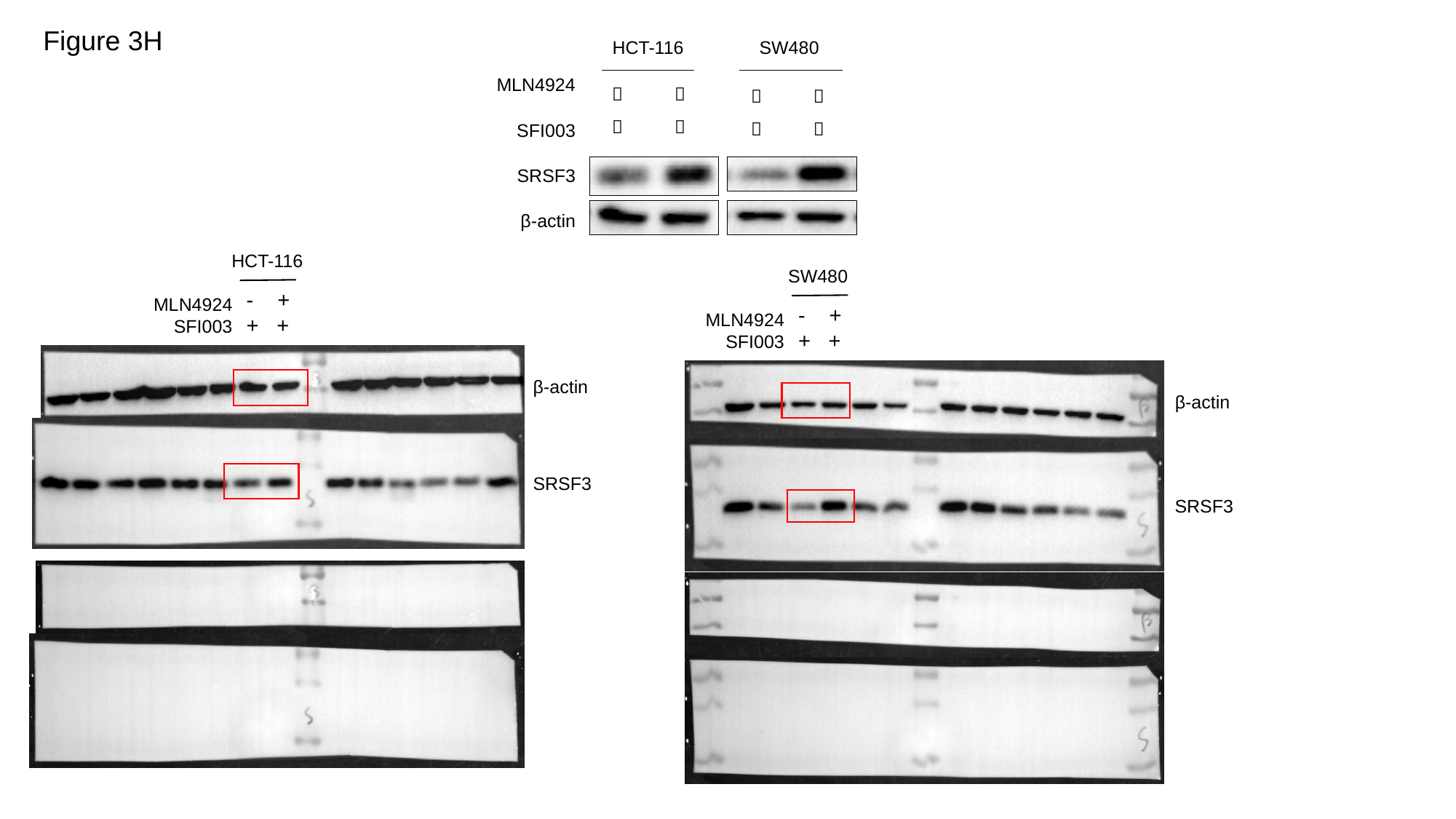

Figure 3H
HCT-116
SW480
| MLN4924 |
| --- |
| SFI003 |
| SRSF3 |
| β-actin |
| － | ＋ |
| --- | --- |
| ＋ | ＋ |
| － | ＋ |
| --- | --- |
| ＋ | ＋ |
HCT-116
SW480
- +
+ +
MLN4924SFI003
- +
+ +
MLN4924SFI003
β-actin
β-actin
SRSF3
SRSF3

## Slide 21
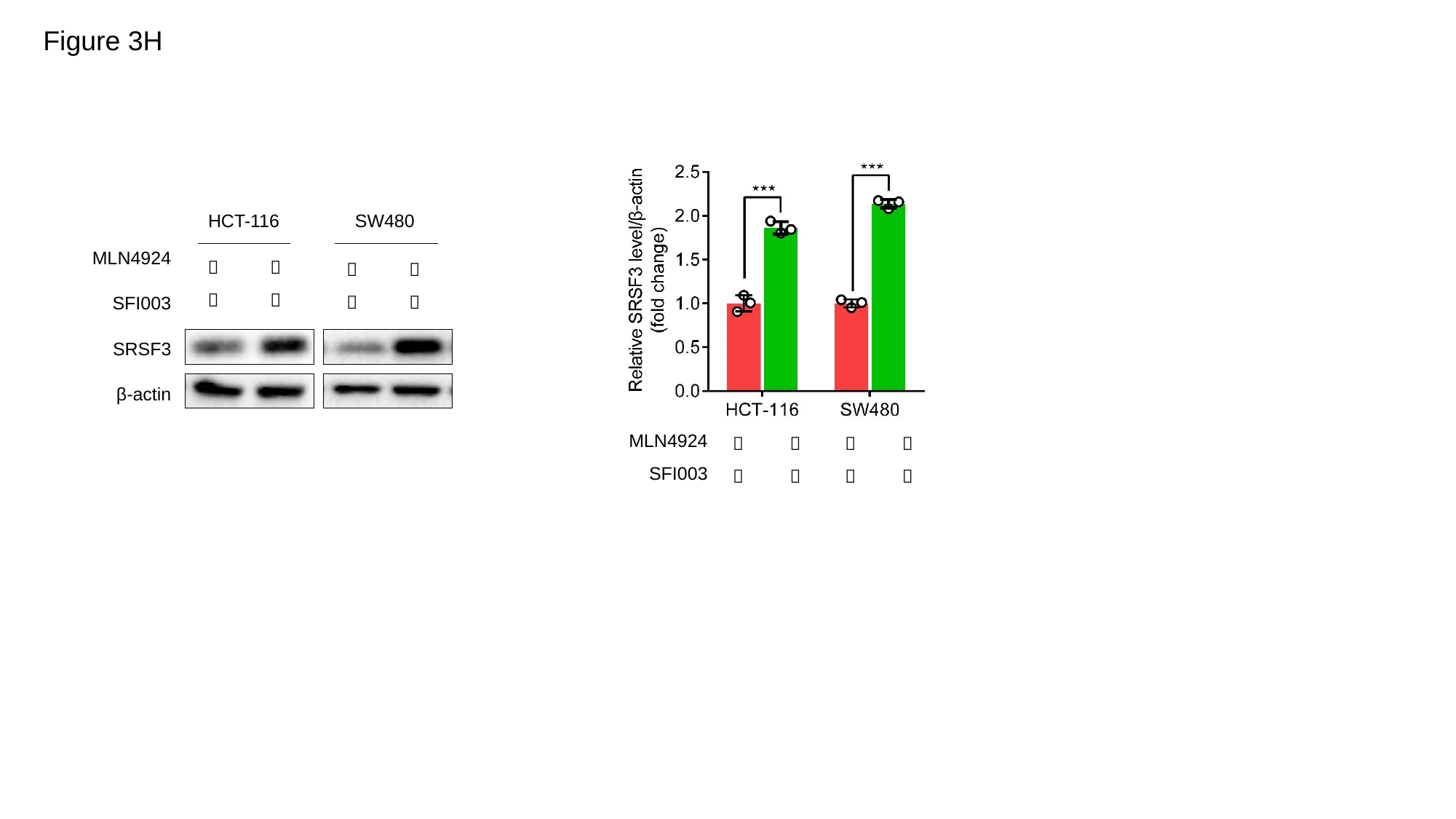

Figure 3H
HCT-116
SW480
| MLN4924 |
| --- |
| SFI003 |
| SRSF3 |
| β-actin |
| － | ＋ |
| --- | --- |
| ＋ | ＋ |
| － | ＋ |
| --- | --- |
| ＋ | ＋ |
| MLN4924 |
| --- |
| SFI003 |
| － | ＋ |
| --- | --- |
| ＋ | ＋ |
| － | ＋ |
| --- | --- |
| ＋ | ＋ |

## Slide 22
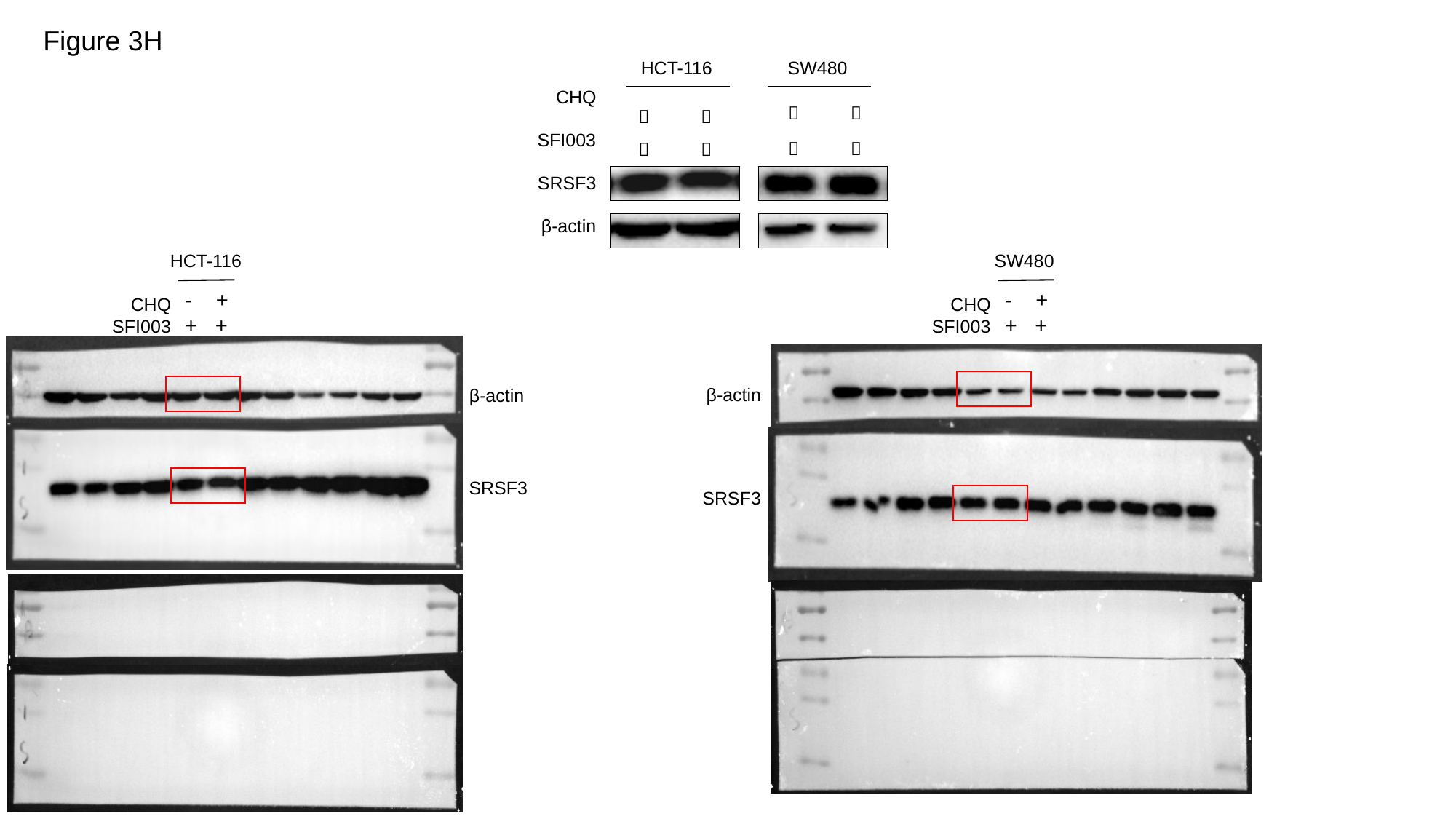

Figure 3H
HCT-116
SW480
| CHQ |
| --- |
| SFI003 |
| SRSF3 |
| β-actin |
| － | ＋ |
| --- | --- |
| ＋ | ＋ |
| － | ＋ |
| --- | --- |
| ＋ | ＋ |
HCT-116
SW480
- +
+ +
- +
+ +
CHQ
SFI003
CHQ
SFI003
β-actin
β-actin
SRSF3
SRSF3

## Slide 23
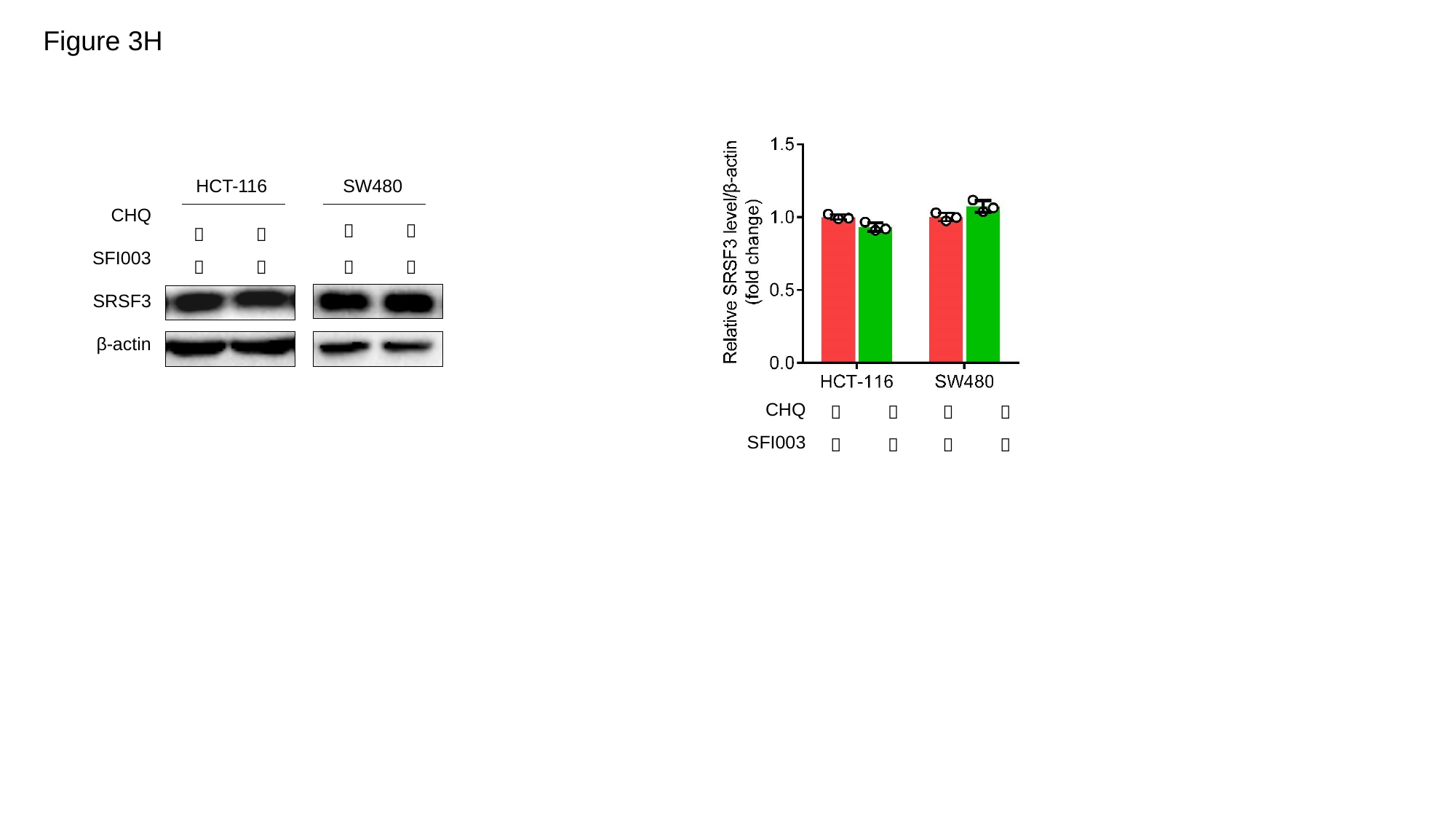

Figure 3H
HCT-116
SW480
| CHQ |
| --- |
| SFI003 |
| SRSF3 |
| β-actin |
| － | ＋ |
| --- | --- |
| ＋ | ＋ |
| － | ＋ |
| --- | --- |
| ＋ | ＋ |
| CHQ |
| --- |
| SFI003 |
| － | ＋ |
| --- | --- |
| ＋ | ＋ |
| － | ＋ |
| --- | --- |
| ＋ | ＋ |

## Slide 24
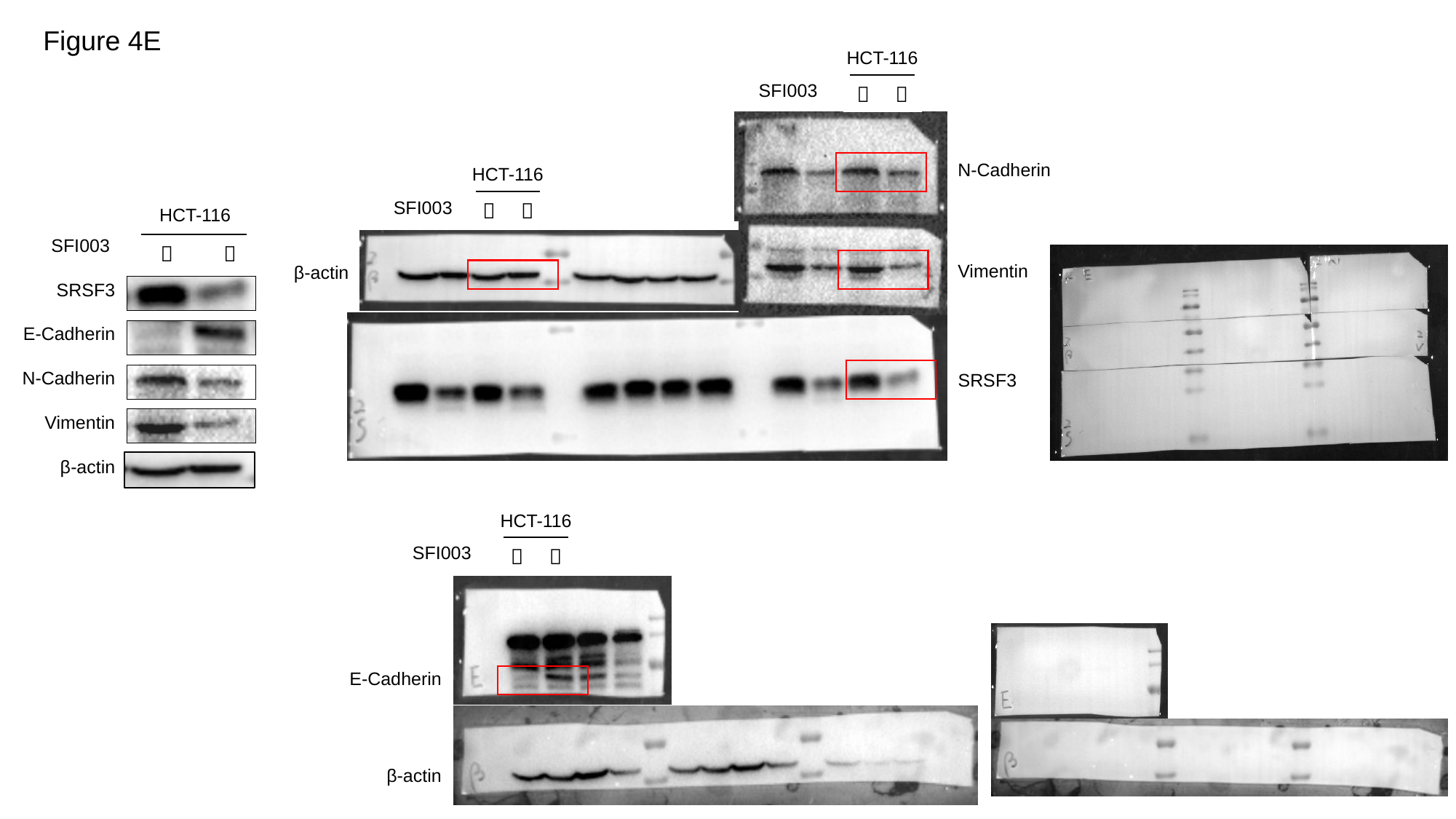

Figure 4E
HCT-116
SFI003
| － | ＋ |
| --- | --- |
N-Cadherin
HCT-116
| － | ＋ |
| --- | --- |
SFI003
HCT-116
| SFI003 |
| --- |
| SRSF3 |
| E-Cadherin |
| N-Cadherin |
| Vimentin |
| β-actin |
| － | ＋ |
| --- | --- |
Vimentin
β-actin
SRSF3
HCT-116
SFI003
| － | ＋ |
| --- | --- |
E-Cadherin
β-actin

## Slide 25
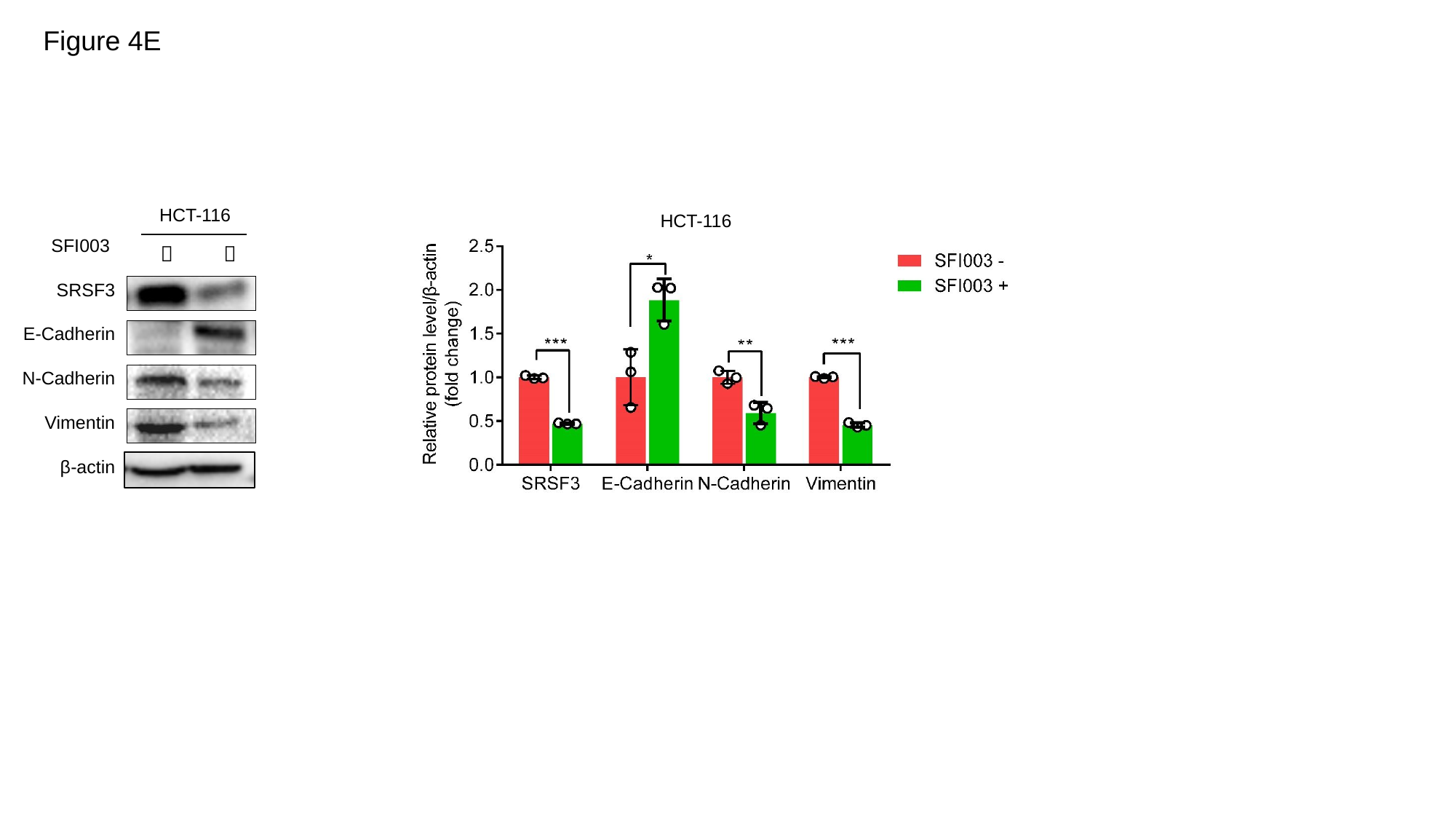

Figure 4E
HCT-116
HCT-116
| SFI003 |
| --- |
| SRSF3 |
| E-Cadherin |
| N-Cadherin |
| Vimentin |
| β-actin |
| － | ＋ |
| --- | --- |

## Slide 26
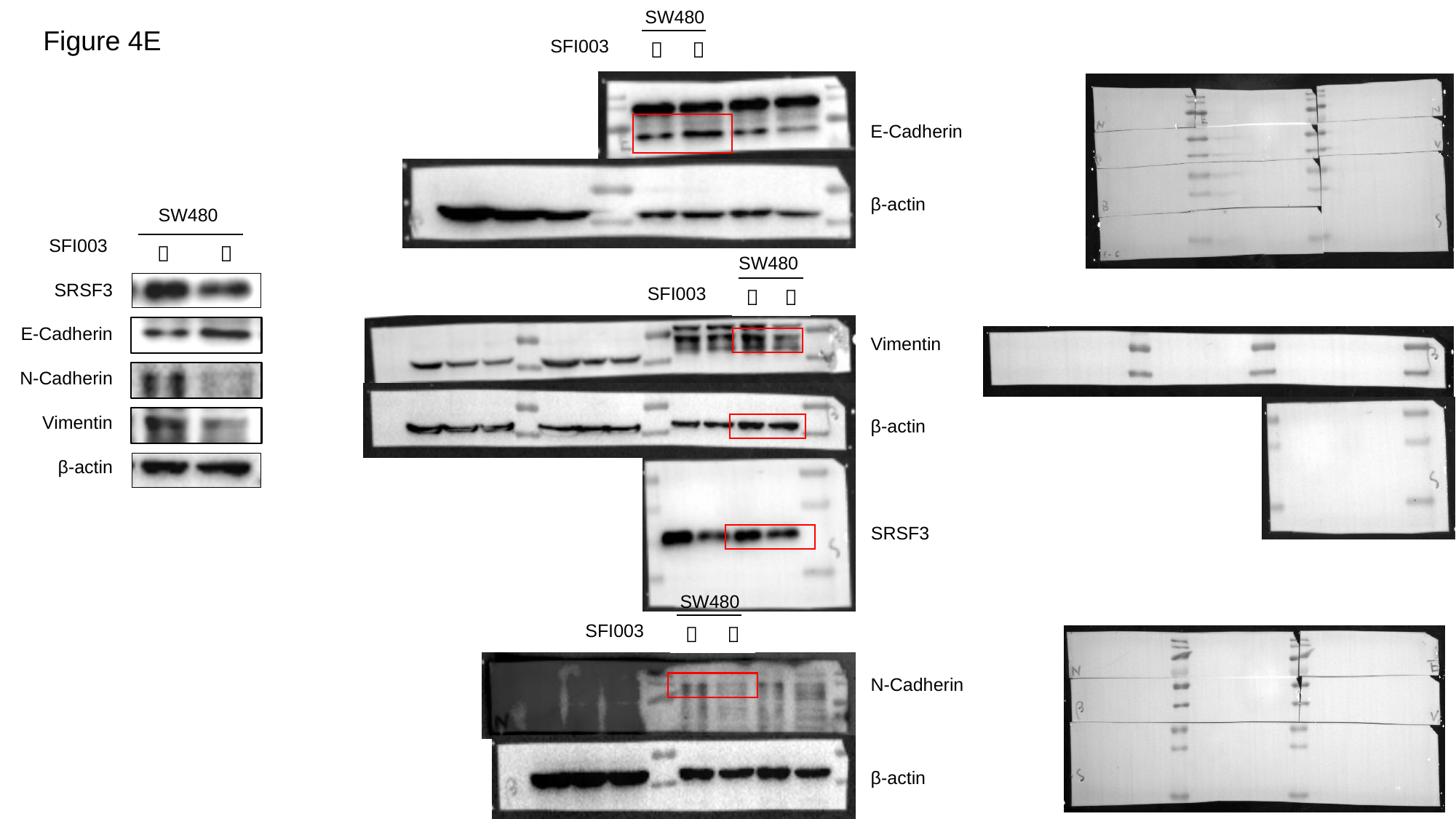

SW480
Figure 4E
SFI003
| － | ＋ |
| --- | --- |
E-Cadherin
β-actin
SW480
| SFI003 |
| --- |
| SRSF3 |
| E-Cadherin |
| N-Cadherin |
| Vimentin |
| β-actin |
| － | ＋ |
| --- | --- |
SW480
SFI003
| － | ＋ |
| --- | --- |
Vimentin
β-actin
SRSF3
SW480
SFI003
| － | ＋ |
| --- | --- |
N-Cadherin
β-actin

## Slide 27
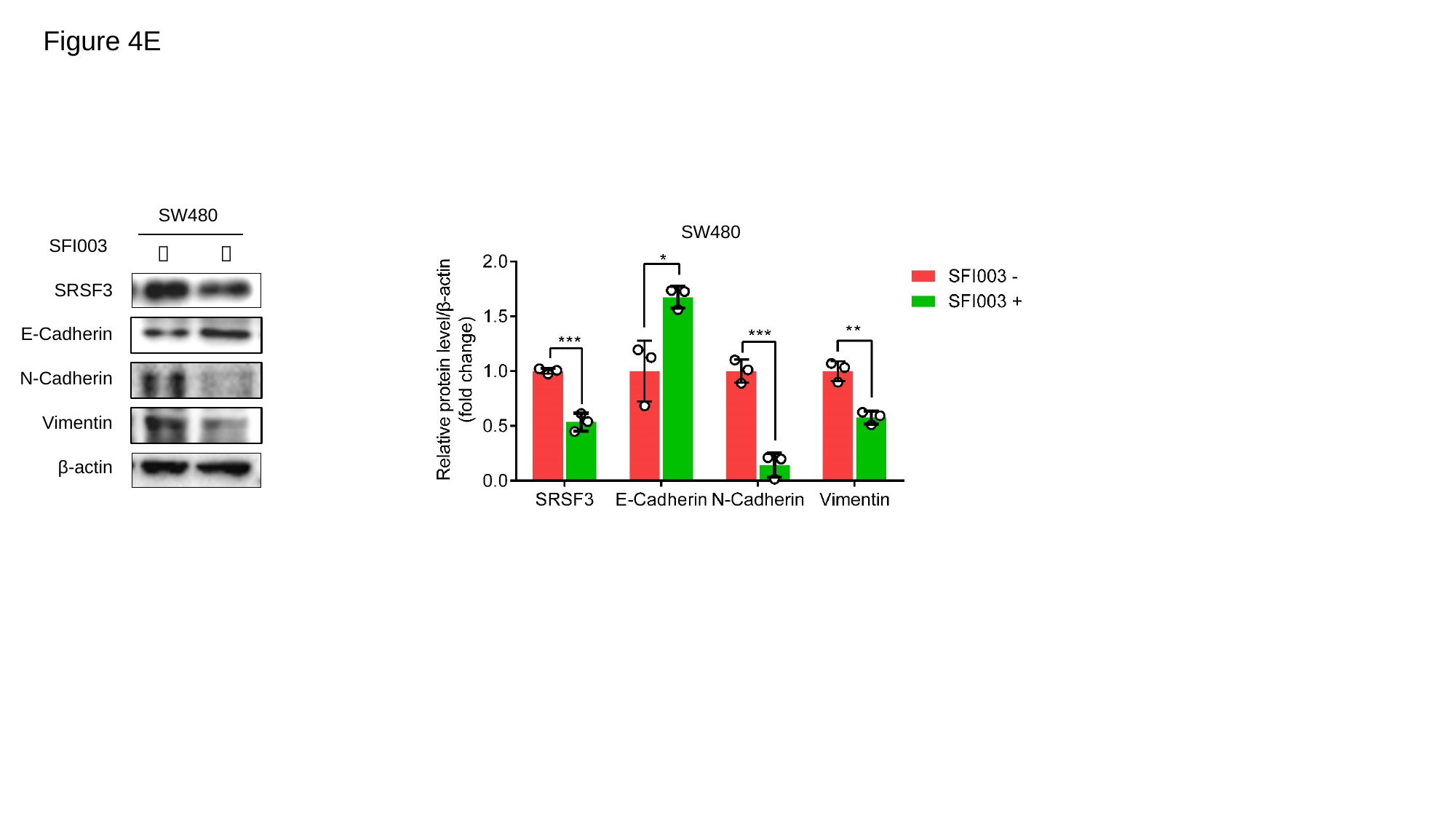

Figure 4E
SW480
SW480
| SFI003 |
| --- |
| SRSF3 |
| E-Cadherin |
| N-Cadherin |
| Vimentin |
| β-actin |
| － | ＋ |
| --- | --- |

## Slide 28
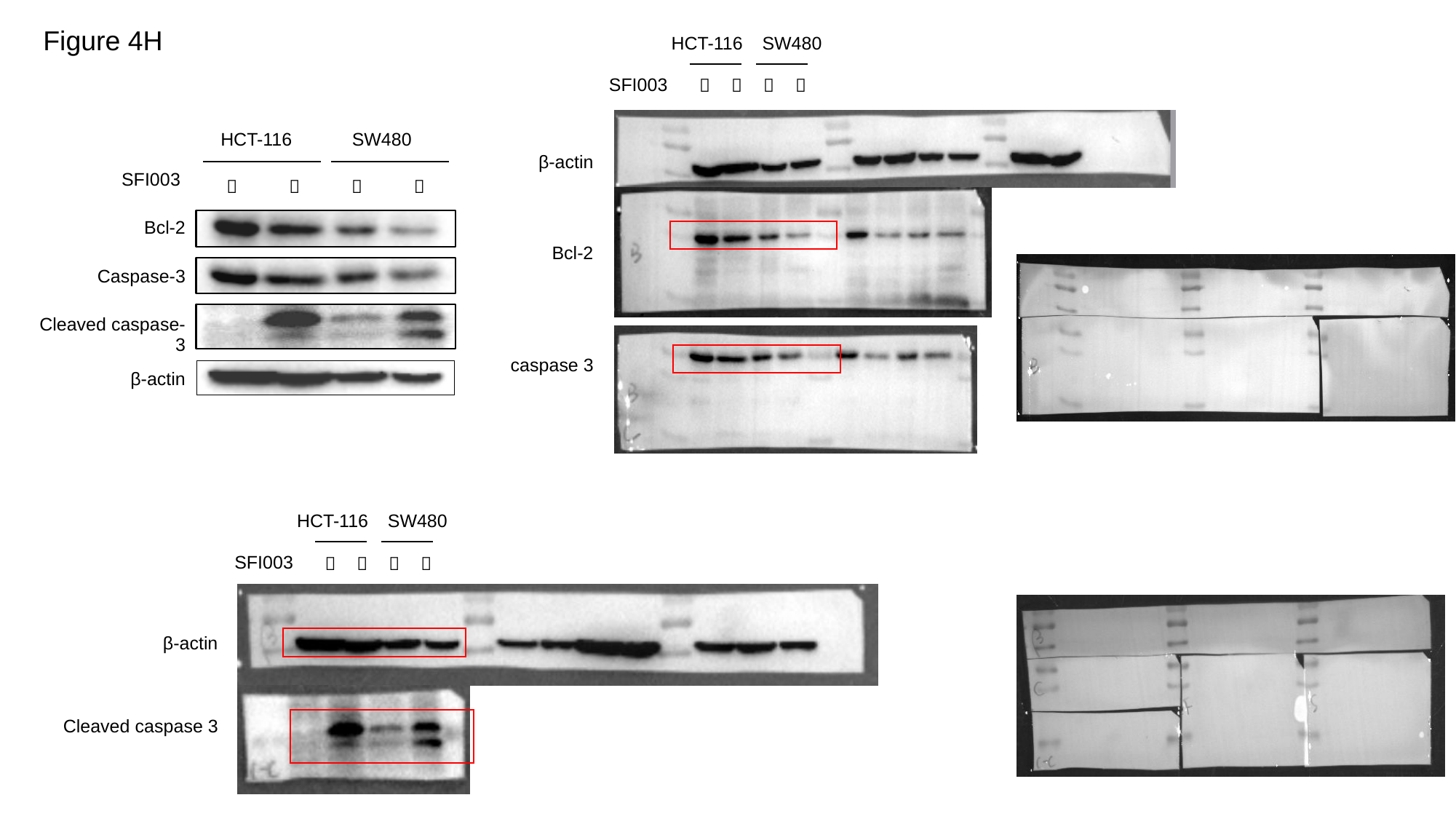

Figure 4H
HCT-116
SW480
SFI003
| － | ＋ | － | ＋ |
| --- | --- | --- | --- |
HCT-116
SW480
β-actin
| SFI003 |
| --- |
| Bcl-2 |
| Caspase-3 |
| Cleaved caspase-3 |
| β-actin |
| － | ＋ | － | ＋ |
| --- | --- | --- | --- |
Bcl-2
caspase 3
HCT-116
SW480
SFI003
| － | ＋ | － | ＋ |
| --- | --- | --- | --- |
β-actin
Cleaved caspase 3

## Slide 29
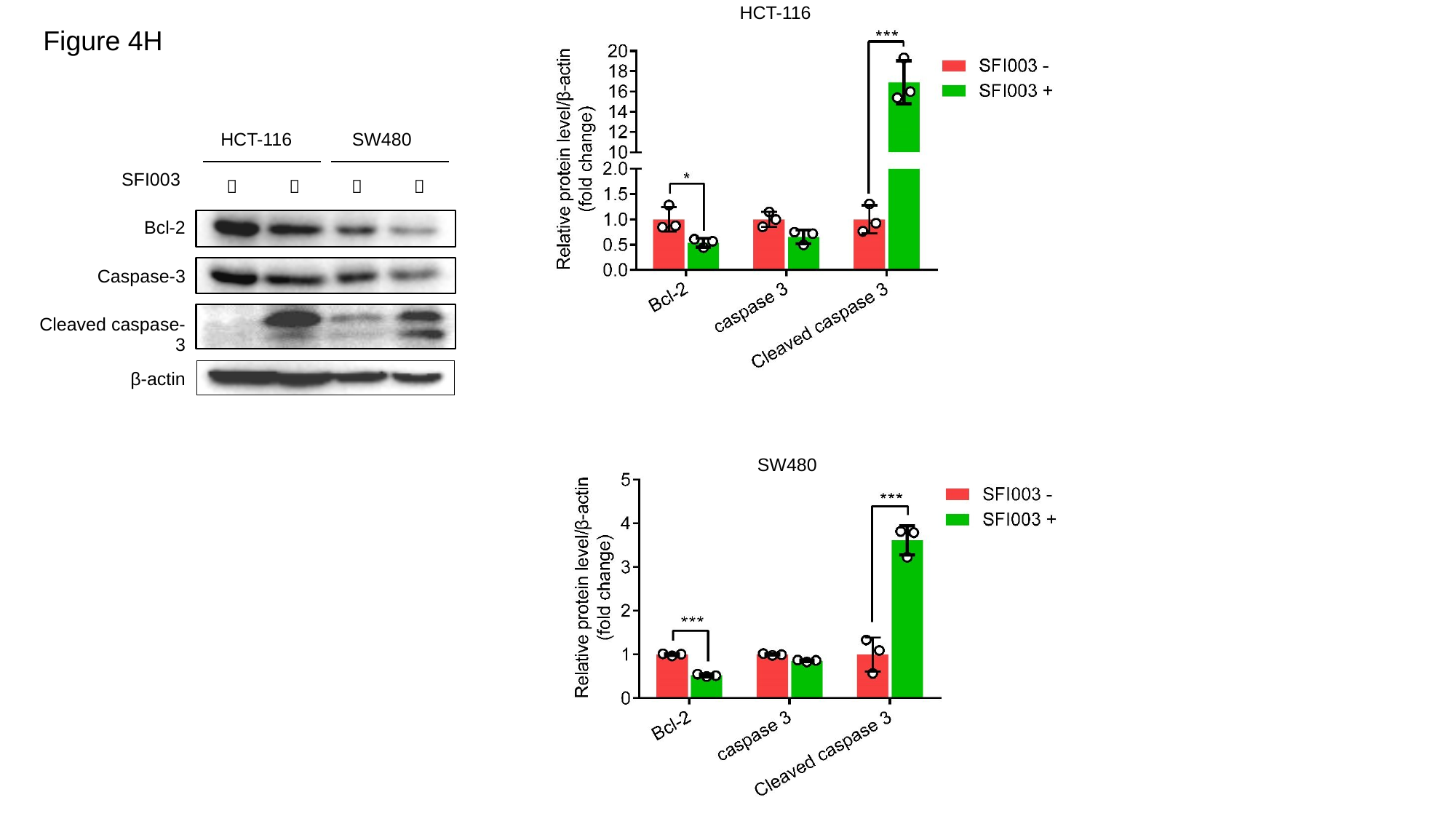

HCT-116
Figure 4H
HCT-116
SW480
| SFI003 |
| --- |
| Bcl-2 |
| Caspase-3 |
| Cleaved caspase-3 |
| β-actin |
| － | ＋ | － | ＋ |
| --- | --- | --- | --- |
SW480

## Slide 30
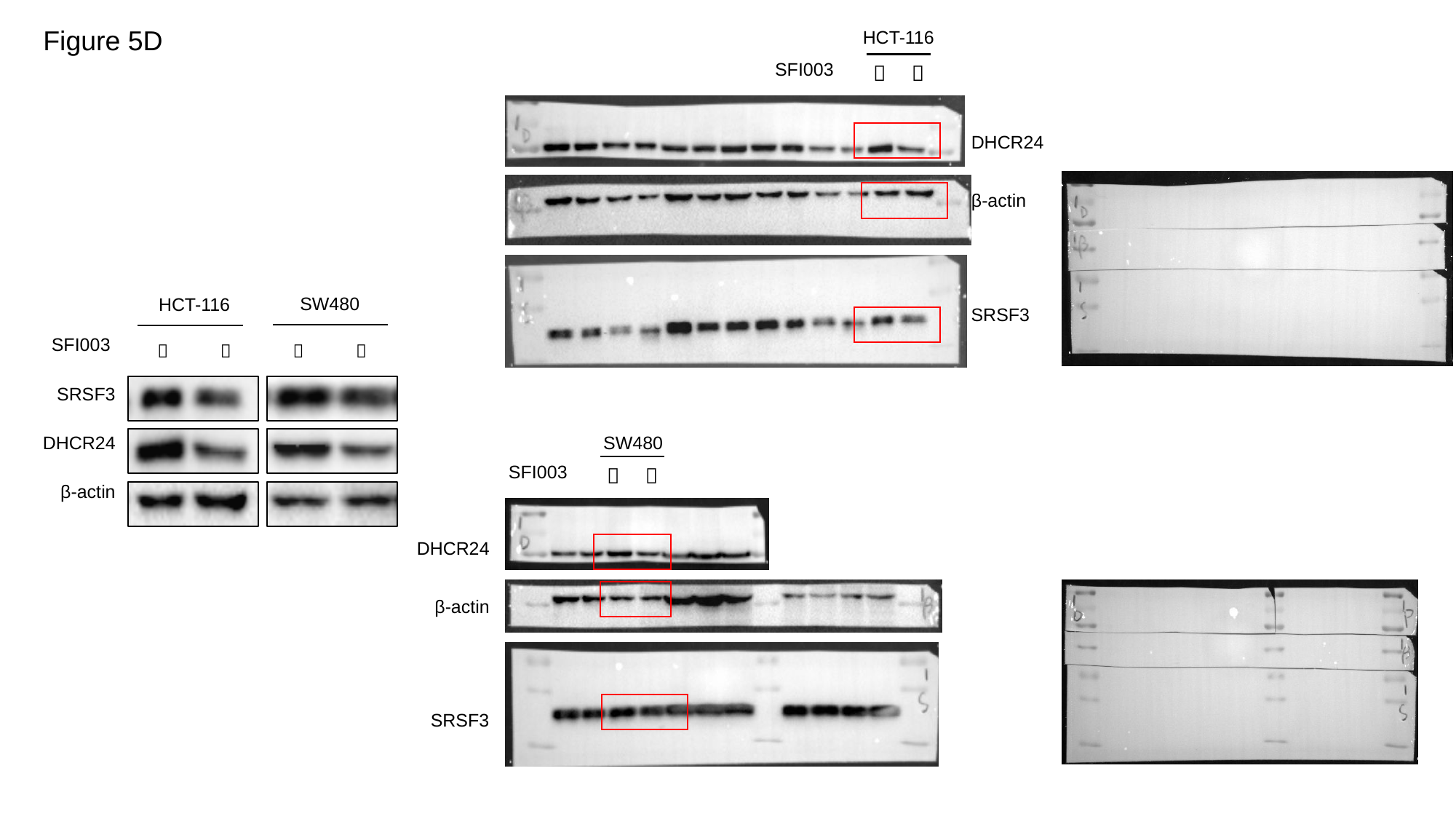

Figure 5D
HCT-116
SFI003
| － | ＋ |
| --- | --- |
DHCR24
β-actin
SW480
HCT-116
SRSF3
| SFI003 |
| --- |
| SRSF3 |
| DHCR24 |
| β-actin |
| － | ＋ |
| --- | --- |
| － | ＋ |
| --- | --- |
SW480
SFI003
| － | ＋ |
| --- | --- |
DHCR24
β-actin
SRSF3

## Slide 31
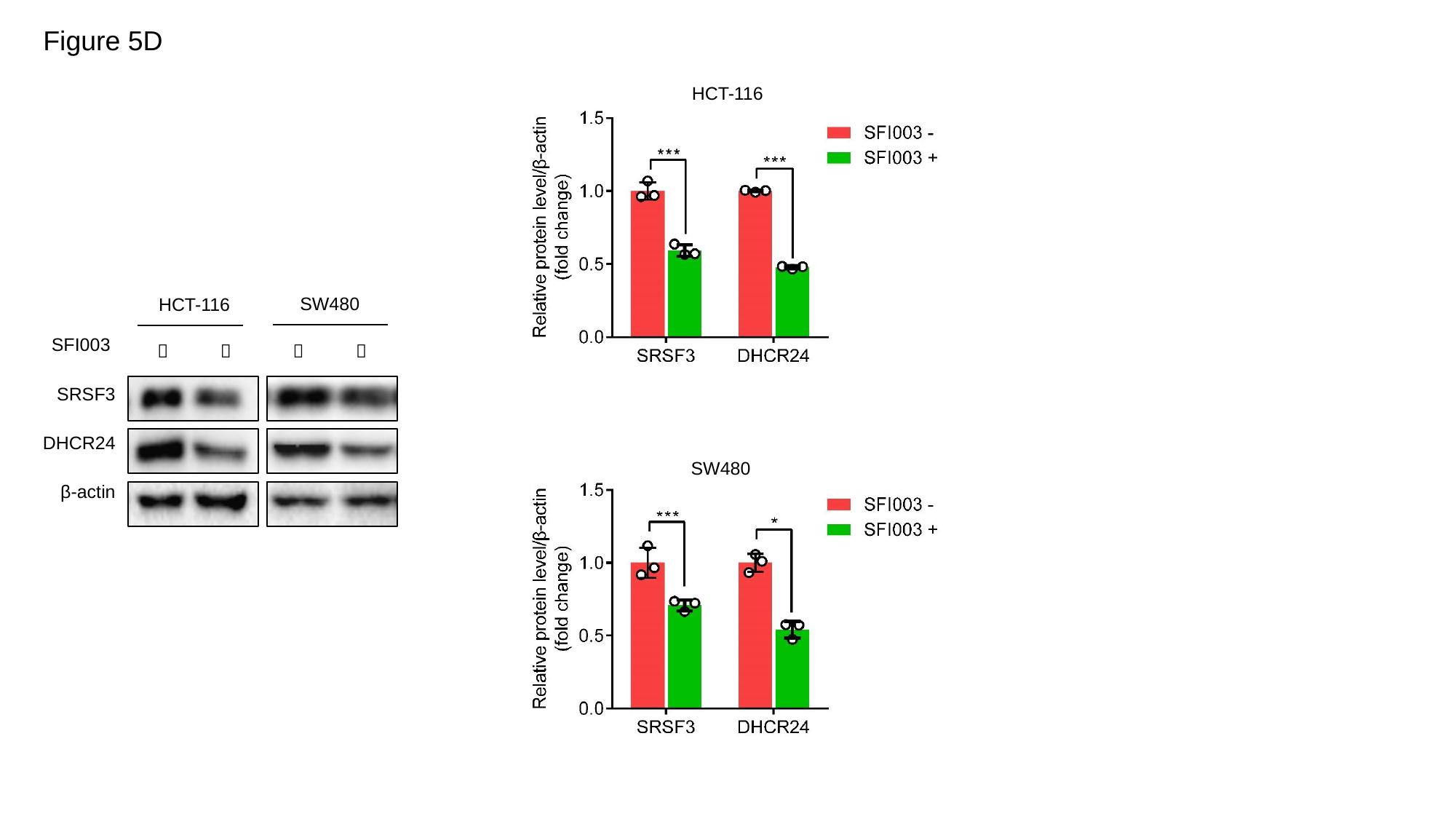

Figure 5D
HCT-116
SW480
HCT-116
| SFI003 |
| --- |
| SRSF3 |
| DHCR24 |
| β-actin |
| － | ＋ |
| --- | --- |
| － | ＋ |
| --- | --- |
SW480

## Slide 32
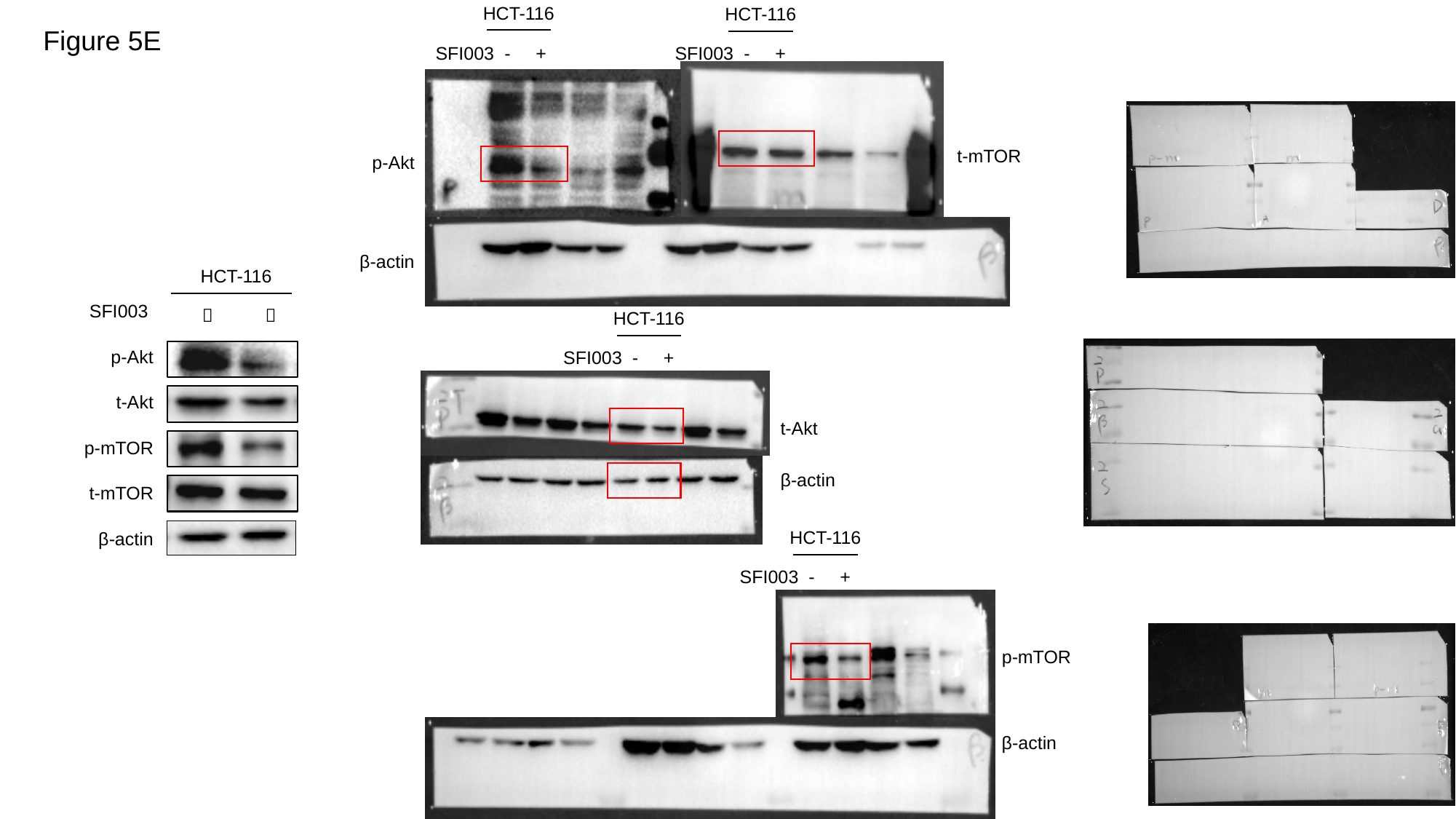

HCT-116
HCT-116
Figure 5E
SFI003 - +
SFI003 - +
t-mTOR
p-Akt
β-actin
HCT-116
| SFI003 |
| --- |
| p-Akt |
| t-Akt |
| p-mTOR |
| t-mTOR |
| β-actin |
| － | ＋ |
| --- | --- |
HCT-116
SFI003 - +
t-Akt
β-actin
HCT-116
SFI003 - +
p-mTOR
β-actin

## Slide 33
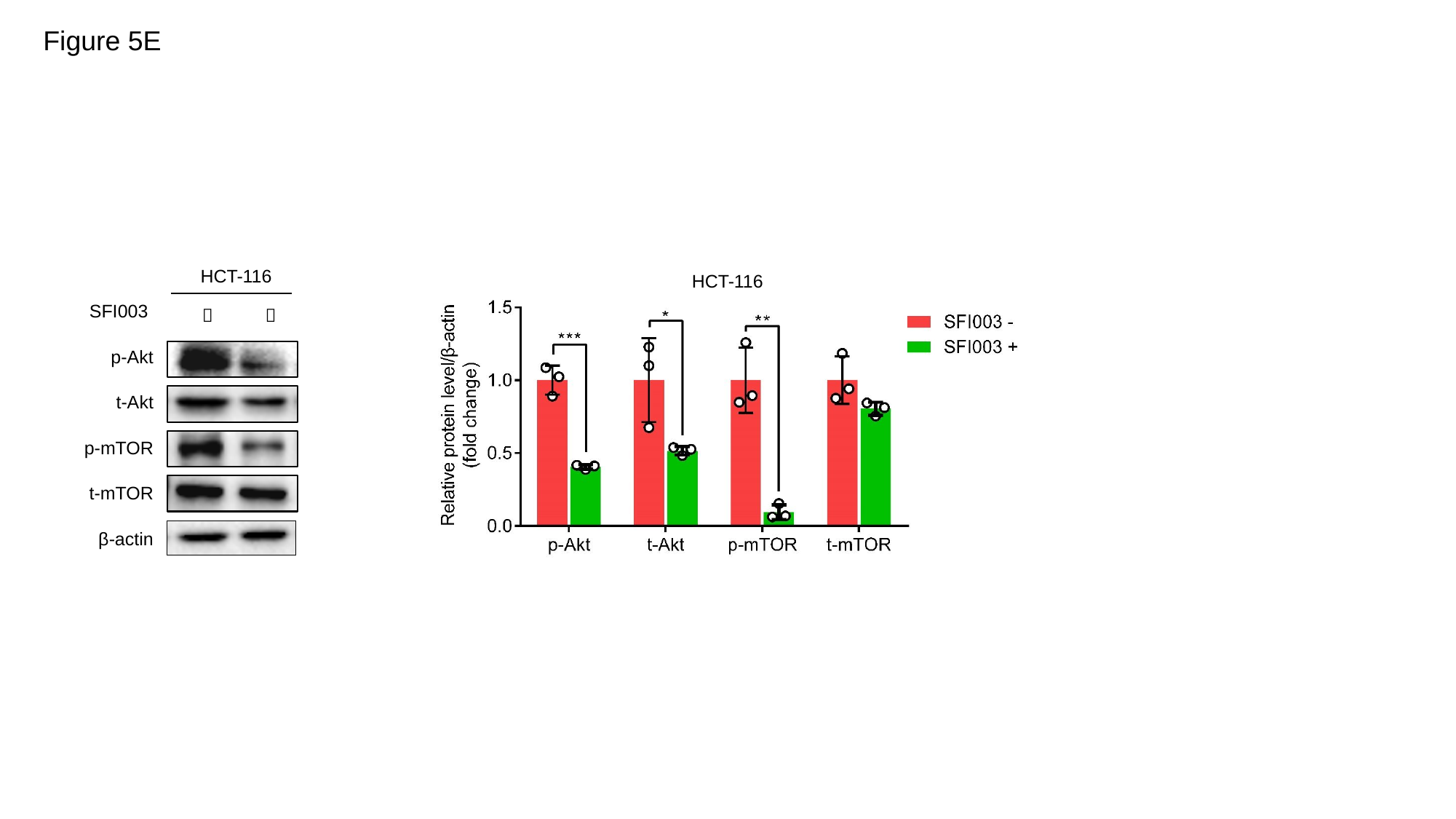

Figure 5E
HCT-116
HCT-116
| SFI003 |
| --- |
| p-Akt |
| t-Akt |
| p-mTOR |
| t-mTOR |
| β-actin |
| － | ＋ |
| --- | --- |

## Slide 34
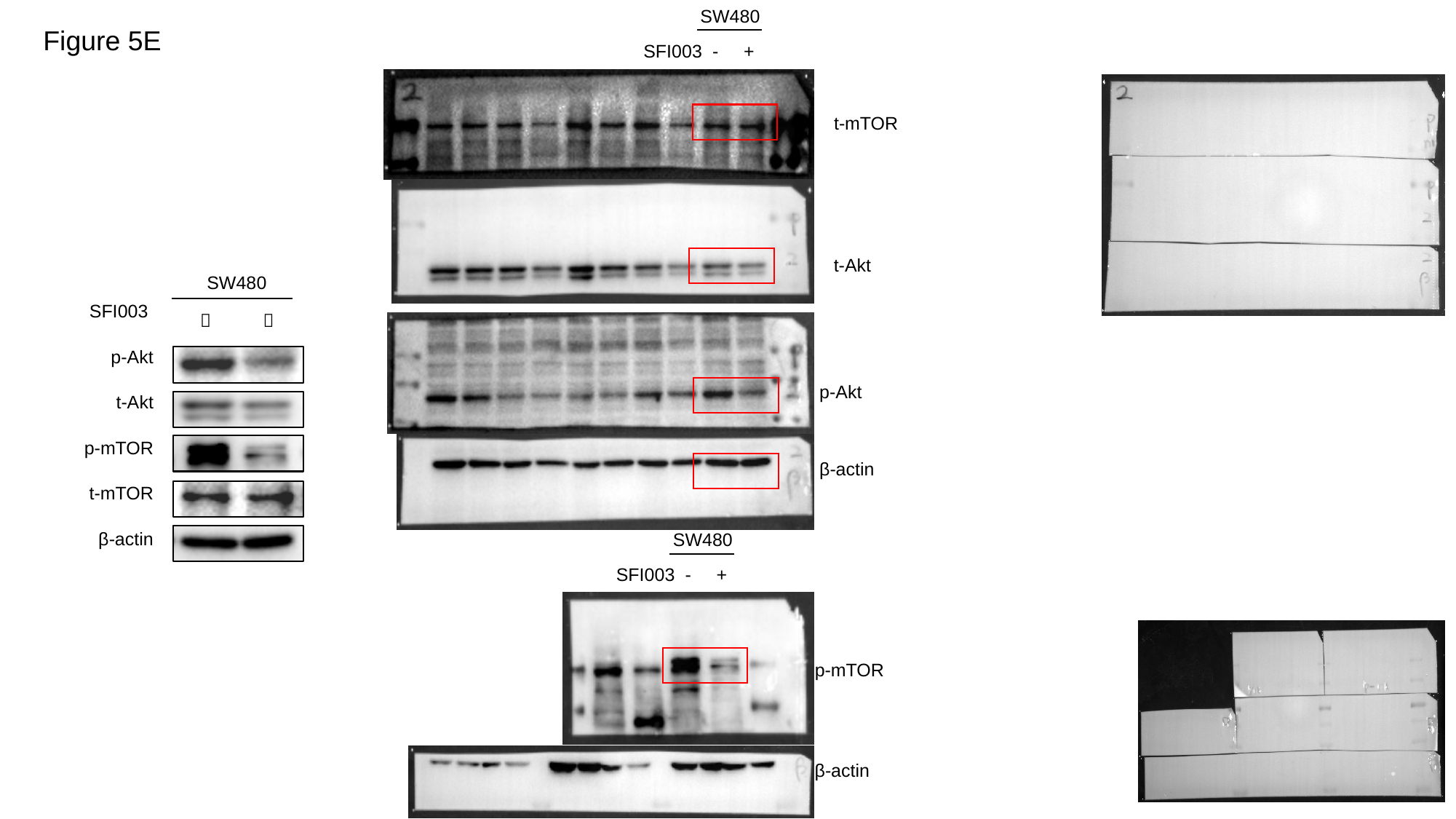

SW480
Figure 5E
| | |
| --- | --- |
SFI003 - +
t-mTOR
t-Akt
SW480
| SFI003 |
| --- |
| p-Akt |
| t-Akt |
| p-mTOR |
| t-mTOR |
| β-actin |
| － | ＋ |
| --- | --- |
p-Akt
β-actin
SW480
| | |
| --- | --- |
SFI003 - +
p-mTOR
β-actin

## Slide 35
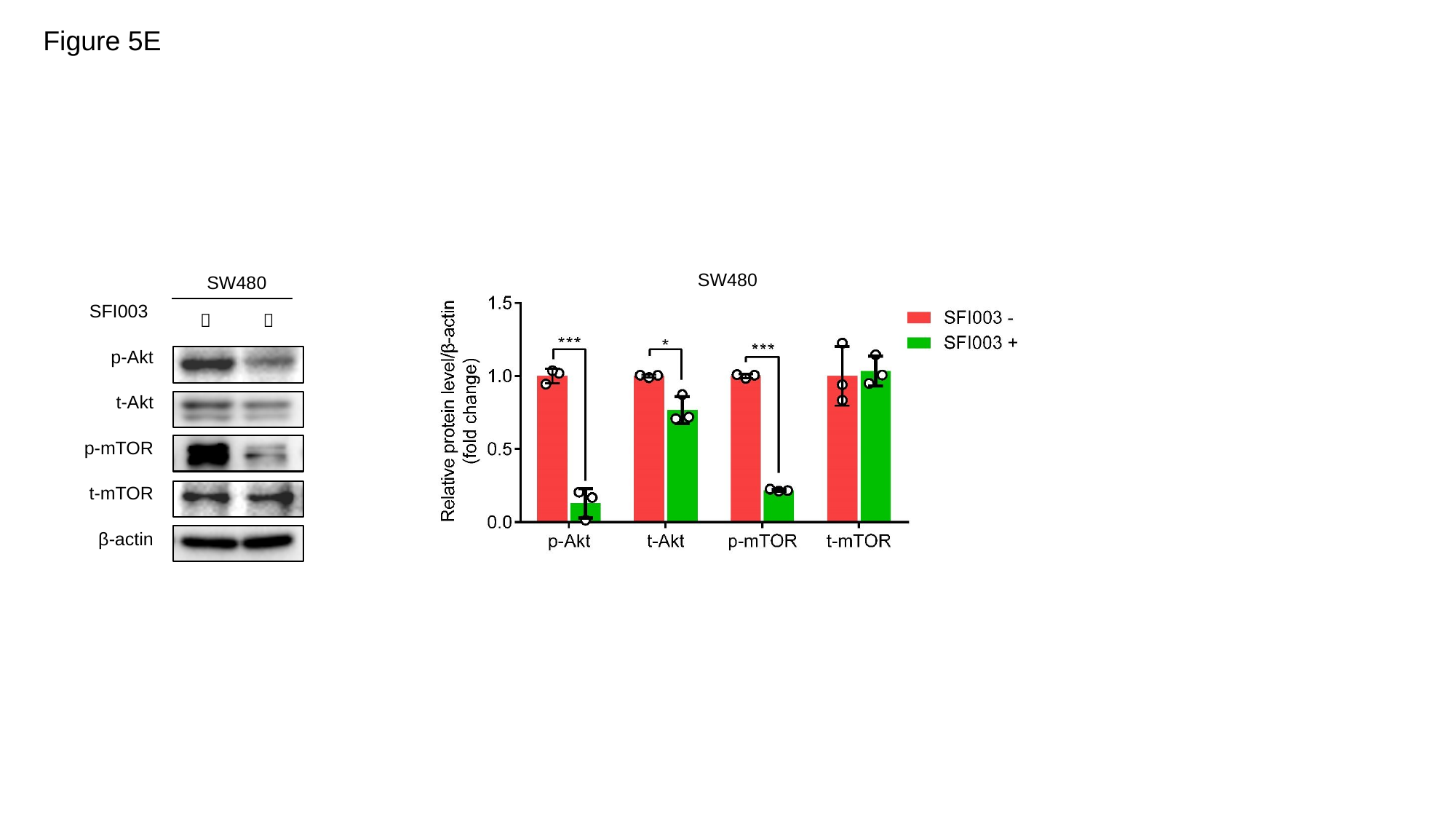

Figure 5E
SW480
SW480
| SFI003 |
| --- |
| p-Akt |
| t-Akt |
| p-mTOR |
| t-mTOR |
| β-actin |
| － | ＋ |
| --- | --- |

## Slide 36
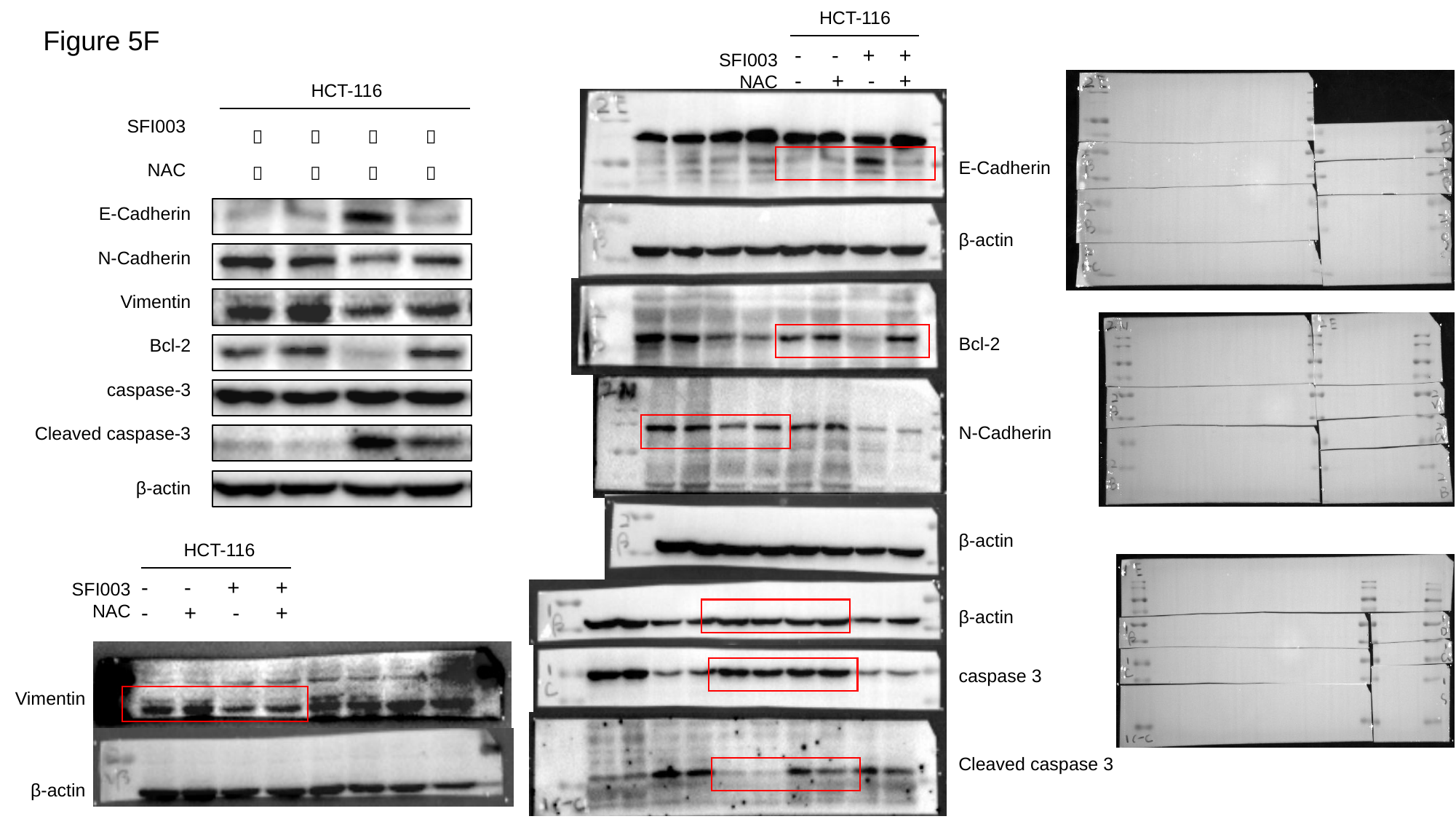

HCT-116
Figure 5F
- - + +
- + - +
SFI003
NAC
HCT-116
| SFI003 |
| --- |
| NAC |
| E-Cadherin |
| N-Cadherin |
| Vimentin |
| Bcl-2 |
| caspase-3 |
| Cleaved caspase-3 |
| β-actin |
| － | － | ＋ | ＋ |
| --- | --- | --- | --- |
| － | ＋ | － | ＋ |
E-Cadherin
β-actin
Bcl-2
N-Cadherin
β-actin
HCT-116
- - + +
- + - +
SFI003
NAC
β-actin
caspase 3
Vimentin
Cleaved caspase 3
β-actin

## Slide 37
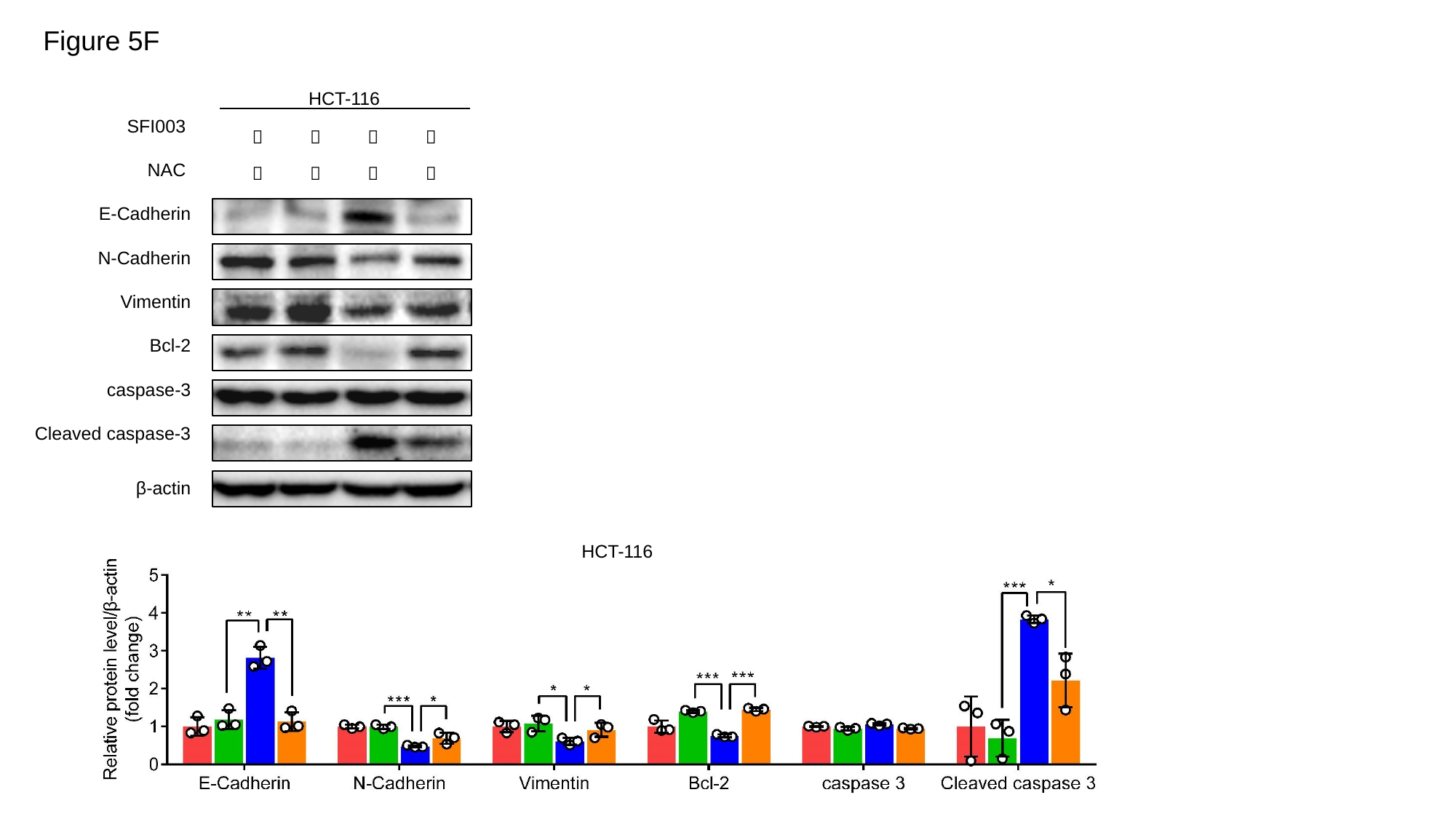

Figure 5F
HCT-116
| SFI003 |
| --- |
| NAC |
| E-Cadherin |
| N-Cadherin |
| Vimentin |
| Bcl-2 |
| caspase-3 |
| Cleaved caspase-3 |
| β-actin |
| － | － | ＋ | ＋ |
| --- | --- | --- | --- |
| － | ＋ | － | ＋ |
HCT-116

## Slide 38
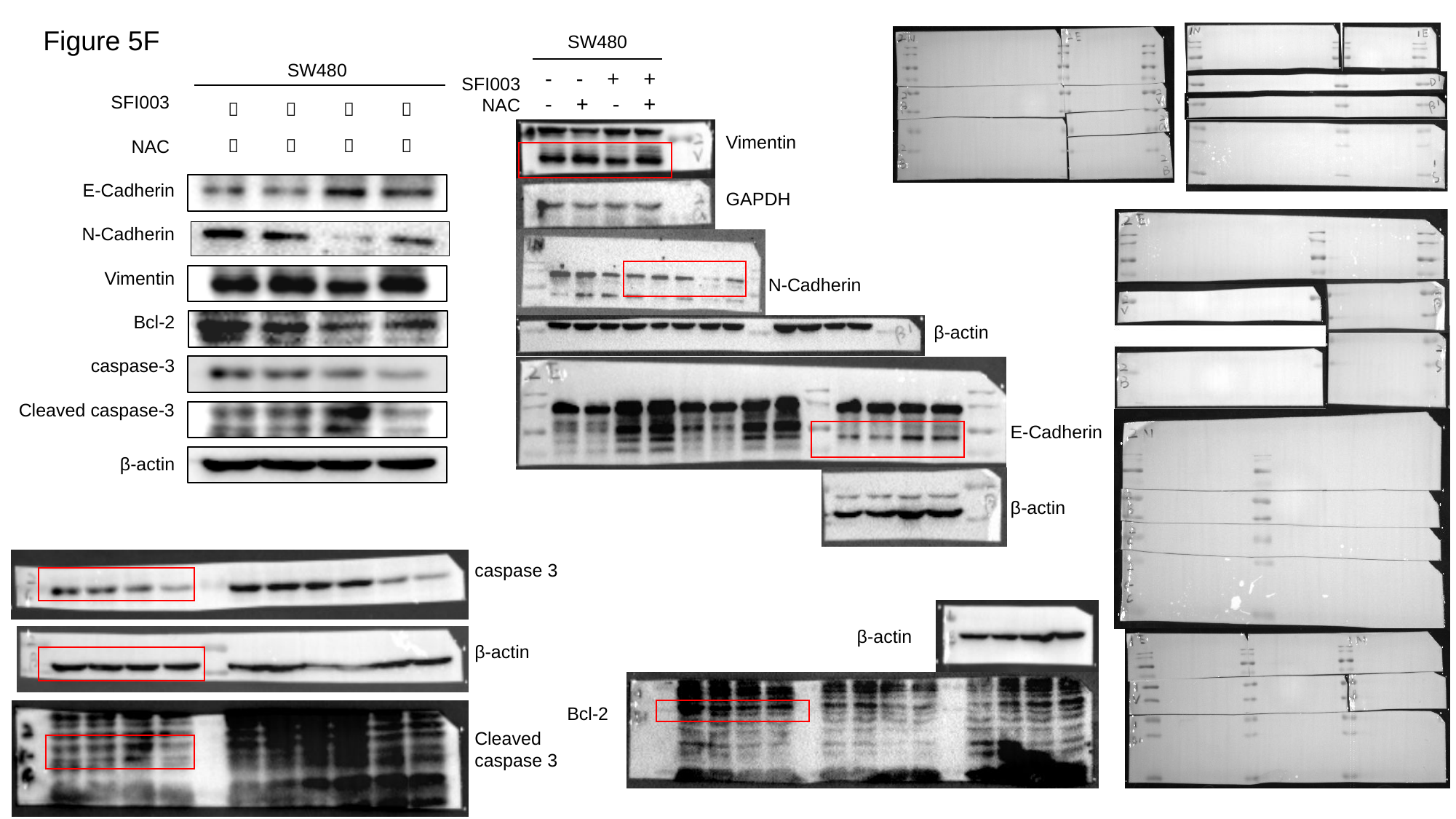

Figure 5F
SW480
SW480
- - + +
- + - +
SFI003
NAC
| SFI003 |
| --- |
| NAC |
| E-Cadherin |
| N-Cadherin |
| Vimentin |
| Bcl-2 |
| caspase-3 |
| Cleaved caspase-3 |
| β-actin |
| － | － | ＋ | ＋ |
| --- | --- | --- | --- |
| － | ＋ | － | ＋ |
Vimentin
GAPDH
N-Cadherin
β-actin
E-Cadherin
β-actin
E-Cadherin
caspase 3
β-actin
β-actin
Bcl-2
Cleaved
caspase 3

## Slide 39
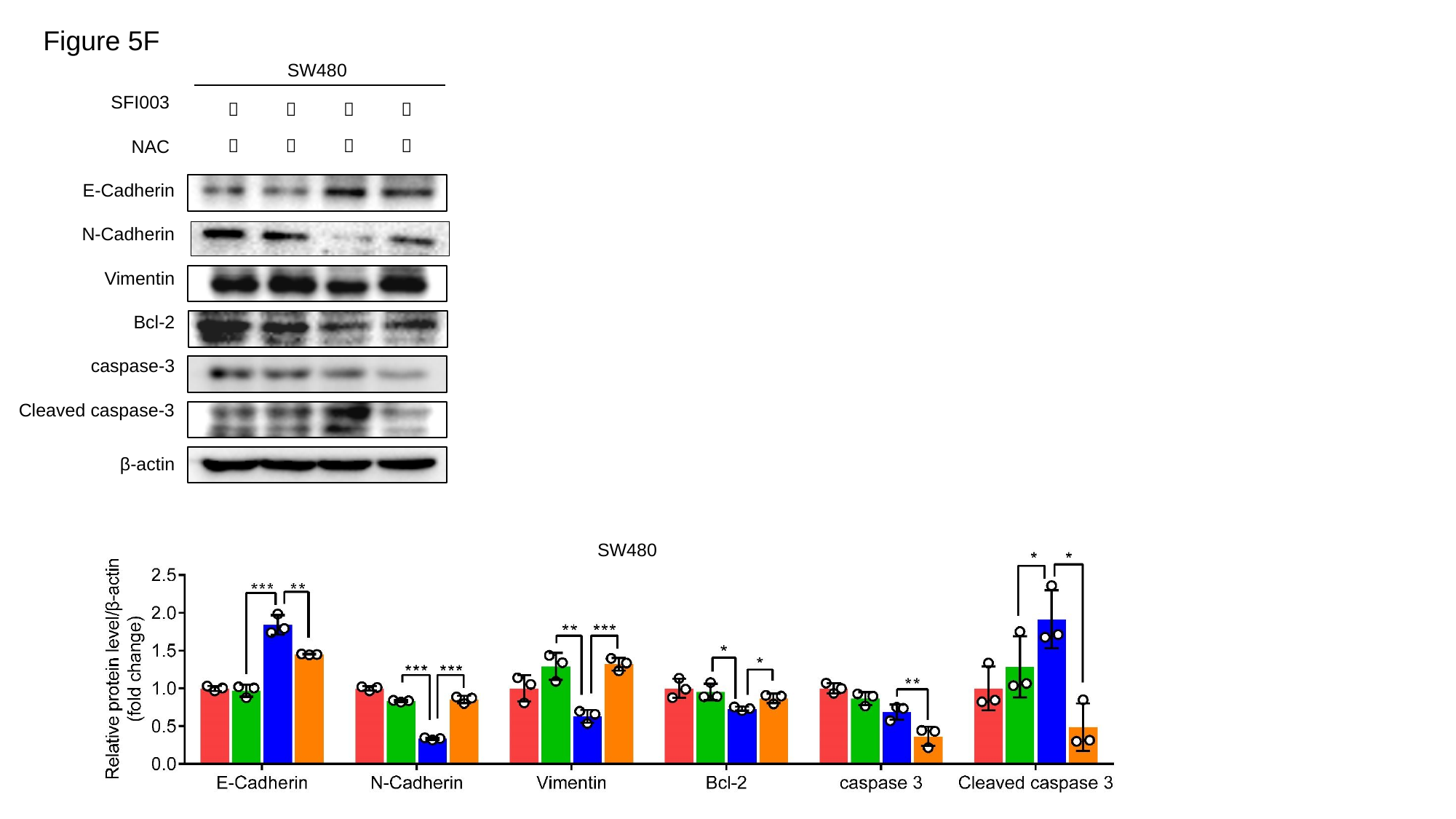

Figure 5F
SW480
| SFI003 |
| --- |
| NAC |
| E-Cadherin |
| N-Cadherin |
| Vimentin |
| Bcl-2 |
| caspase-3 |
| Cleaved caspase-3 |
| β-actin |
| － | － | ＋ | ＋ |
| --- | --- | --- | --- |
| － | ＋ | － | ＋ |
SW480

## Slide 40
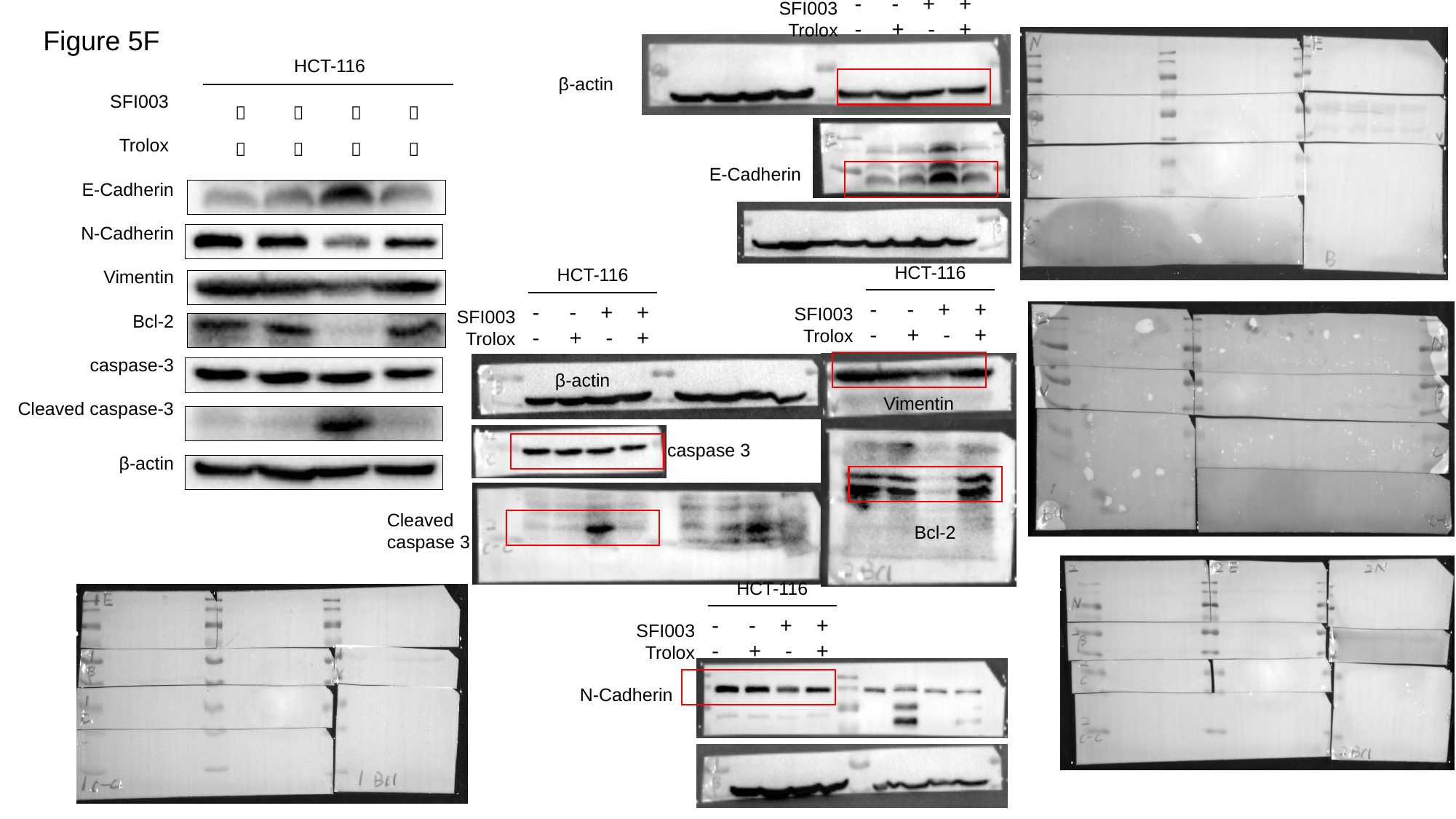

HCT-116
- - + +
- + - +
SFI003
Trolox
Figure 5F
HCT-116
β-actin
| SFI003 |
| --- |
| Trolox |
| E-Cadherin |
| N-Cadherin |
| Vimentin |
| Bcl-2 |
| caspase-3 |
| Cleaved caspase-3 |
| β-actin |
| － | － | ＋ | ＋ |
| --- | --- | --- | --- |
| － | ＋ | － | ＋ |
E-Cadherin
HCT-116
HCT-116
- - + +
- + - +
- - + +
- + - +
SFI003
Trolox
SFI003
Trolox
β-actin
Vimentin
caspase 3
Cleaved
caspase 3
Bcl-2
HCT-116
- - + +
- + - +
SFI003
Trolox
N-Cadherin

## Slide 41
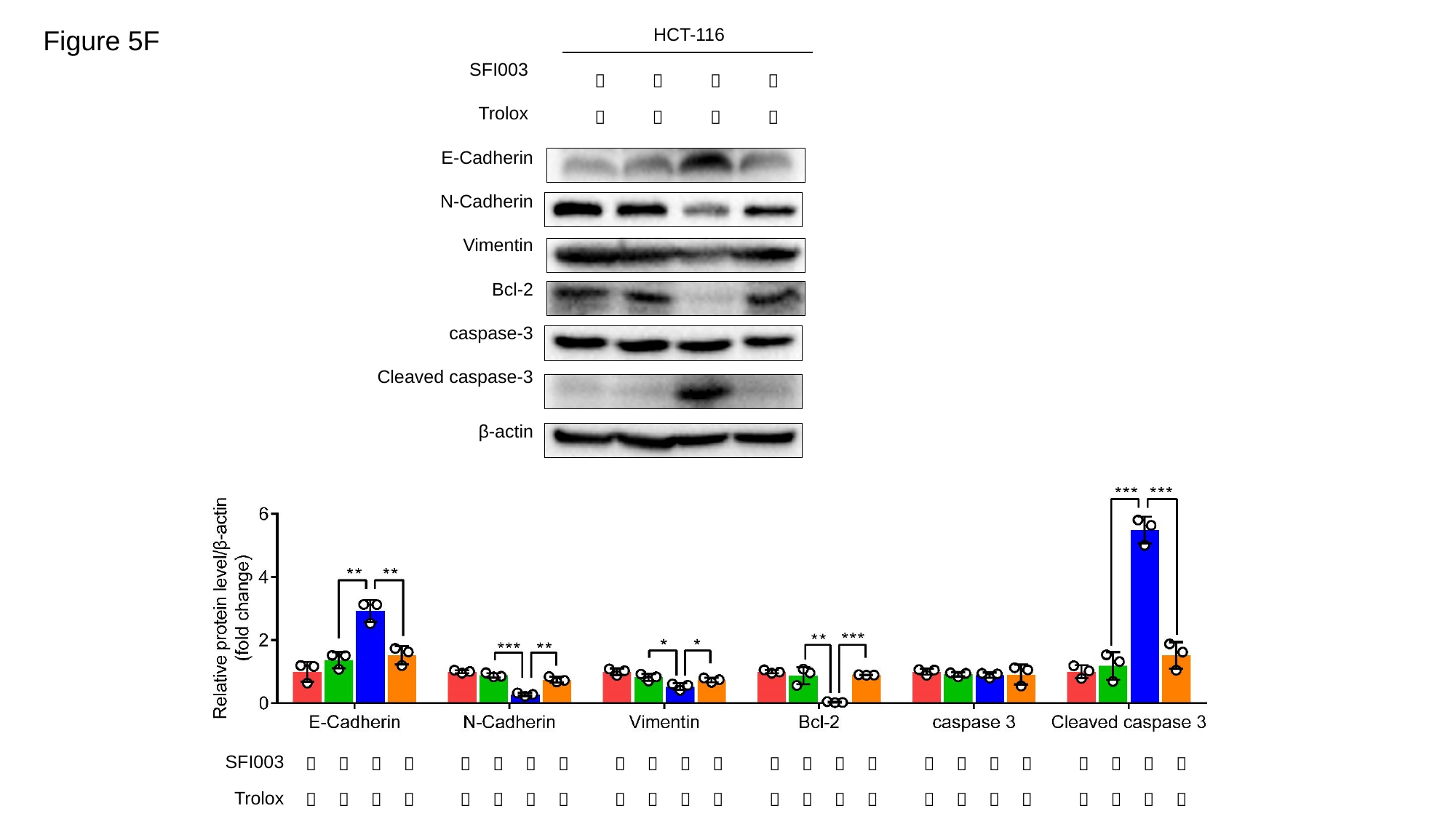

HCT-116
Figure 5F
| SFI003 |
| --- |
| Trolox |
| E-Cadherin |
| N-Cadherin |
| Vimentin |
| Bcl-2 |
| caspase-3 |
| Cleaved caspase-3 |
| β-actin |
| － | － | ＋ | ＋ |
| --- | --- | --- | --- |
| － | ＋ | － | ＋ |
| － | － | ＋ | ＋ |
| --- | --- | --- | --- |
| － | ＋ | － | ＋ |
| － | － | ＋ | ＋ |
| --- | --- | --- | --- |
| － | ＋ | － | ＋ |
| － | － | ＋ | ＋ |
| --- | --- | --- | --- |
| － | ＋ | － | ＋ |
| － | － | ＋ | ＋ |
| --- | --- | --- | --- |
| － | ＋ | － | ＋ |
| － | － | ＋ | ＋ |
| --- | --- | --- | --- |
| － | ＋ | － | ＋ |
| － | － | ＋ | ＋ |
| --- | --- | --- | --- |
| － | ＋ | － | ＋ |
| SFI003 |
| --- |
| Trolox |

## Slide 42
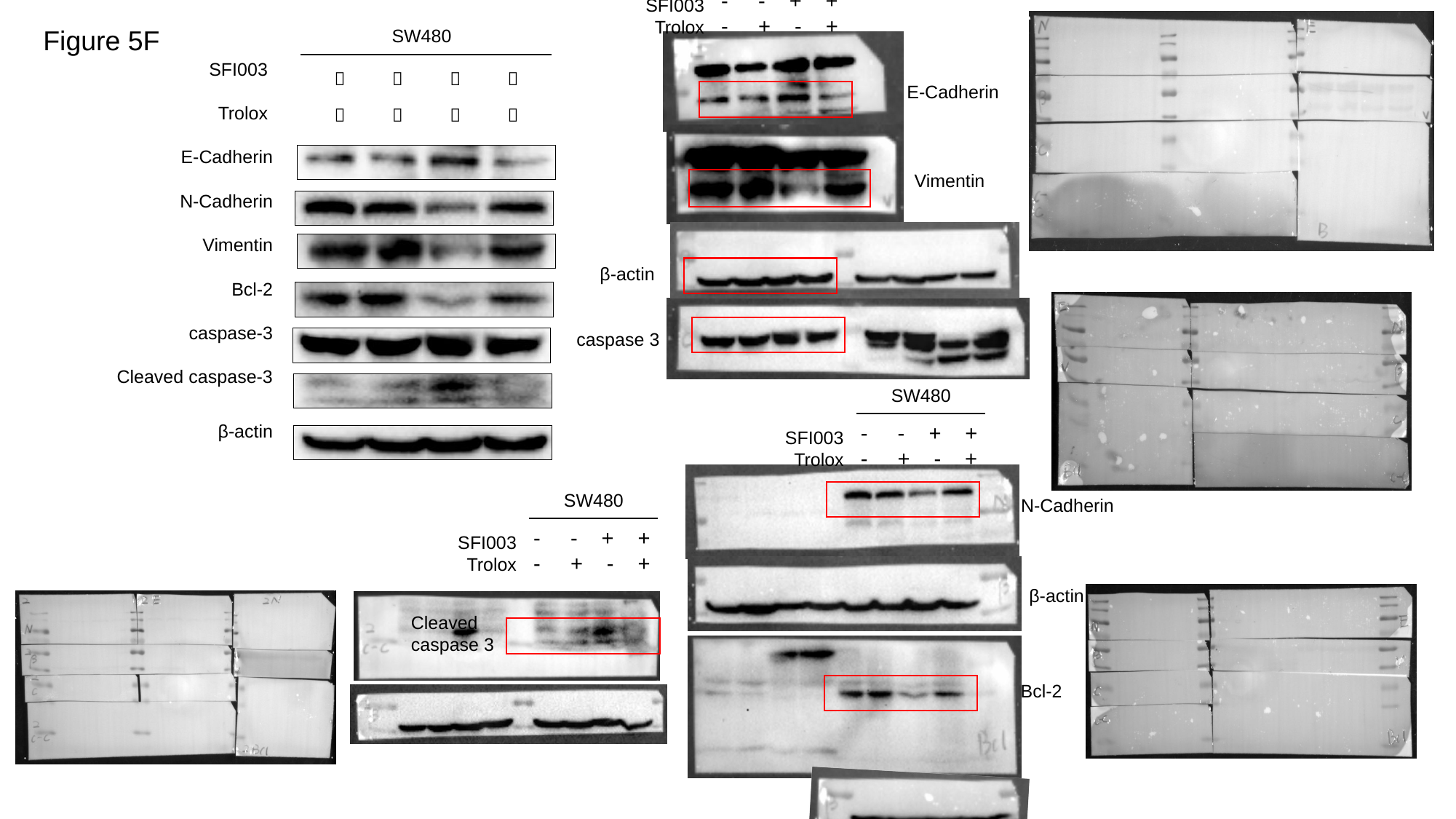

SW480
- - + +
- + - +
SFI003
Trolox
Figure 5F
SW480
| SFI003 |
| --- |
| Trolox |
| E-Cadherin |
| N-Cadherin |
| Vimentin |
| Bcl-2 |
| caspase-3 |
| Cleaved caspase-3 |
| β-actin |
| － | － | ＋ | ＋ |
| --- | --- | --- | --- |
| － | ＋ | － | ＋ |
E-Cadherin
Vimentin
β-actin
caspase 3
SW480
- - + +
- + - +
SFI003
Trolox
SW480
N-Cadherin
- - + +
- + - +
SFI003
Trolox
β-actin
Cleaved
caspase 3
Bcl-2

## Slide 43
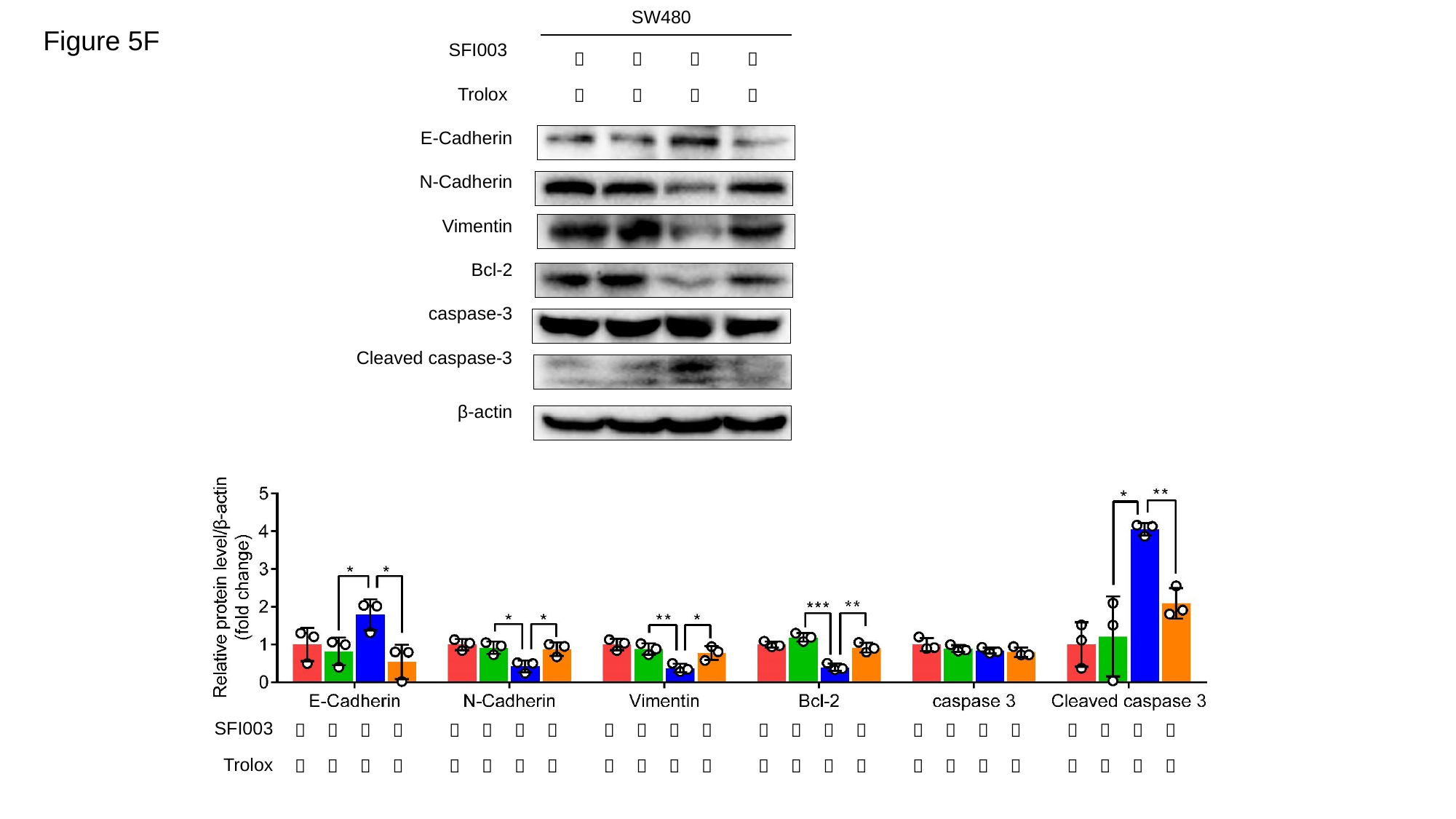

SW480
Figure 5F
| SFI003 |
| --- |
| Trolox |
| E-Cadherin |
| N-Cadherin |
| Vimentin |
| Bcl-2 |
| caspase-3 |
| Cleaved caspase-3 |
| β-actin |
| － | － | ＋ | ＋ |
| --- | --- | --- | --- |
| － | ＋ | － | ＋ |
| － | － | ＋ | ＋ |
| --- | --- | --- | --- |
| － | ＋ | － | ＋ |
| － | － | ＋ | ＋ |
| --- | --- | --- | --- |
| － | ＋ | － | ＋ |
| － | － | ＋ | ＋ |
| --- | --- | --- | --- |
| － | ＋ | － | ＋ |
| － | － | ＋ | ＋ |
| --- | --- | --- | --- |
| － | ＋ | － | ＋ |
| － | － | ＋ | ＋ |
| --- | --- | --- | --- |
| － | ＋ | － | ＋ |
| － | － | ＋ | ＋ |
| --- | --- | --- | --- |
| － | ＋ | － | ＋ |
| SFI003 |
| --- |
| Trolox |

## Slide 44
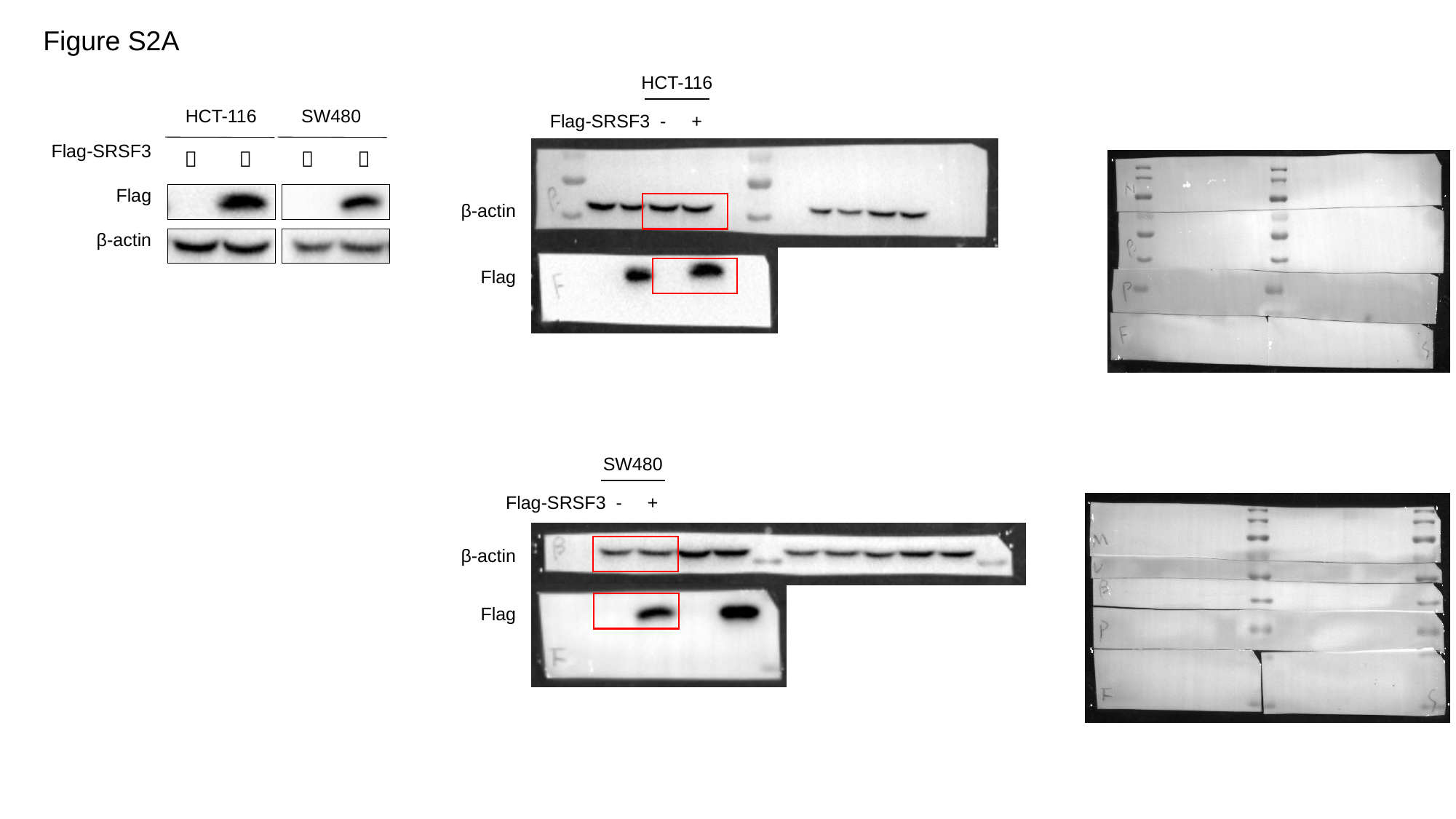

Figure S2A
HCT-116
HCT-116
SW480
Flag-SRSF3 - +
| Flag-SRSF3 |
| --- |
| Flag |
| β-actin |
| － | ＋ |
| --- | --- |
| － | ＋ |
| --- | --- |
β-actin
Flag
SW480
Flag-SRSF3 - +
β-actin
Flag

## Slide 45
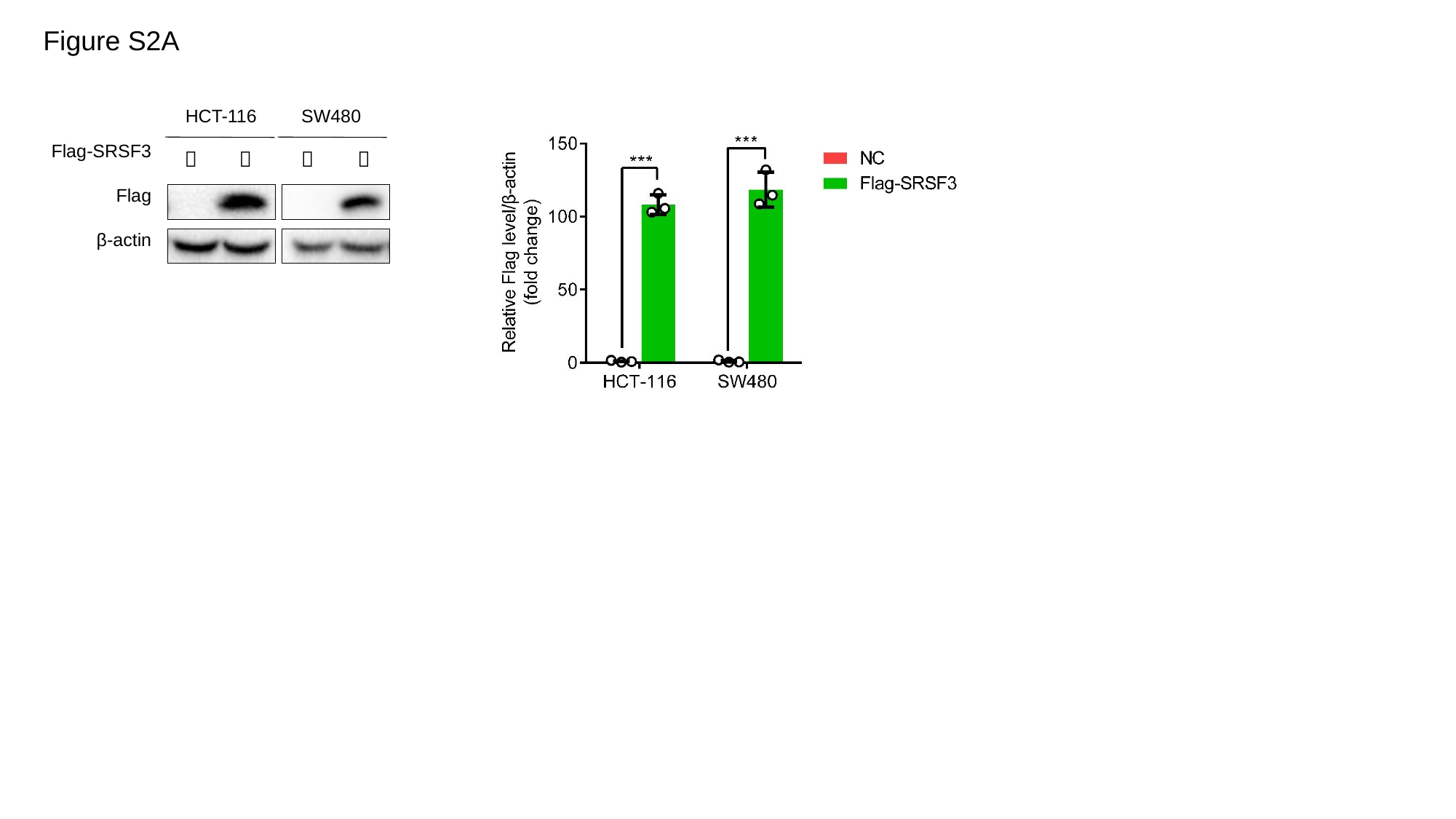

Figure S2A
HCT-116
SW480
| Flag-SRSF3 |
| --- |
| Flag |
| β-actin |
| － | ＋ |
| --- | --- |
| － | ＋ |
| --- | --- |

## Slide 46
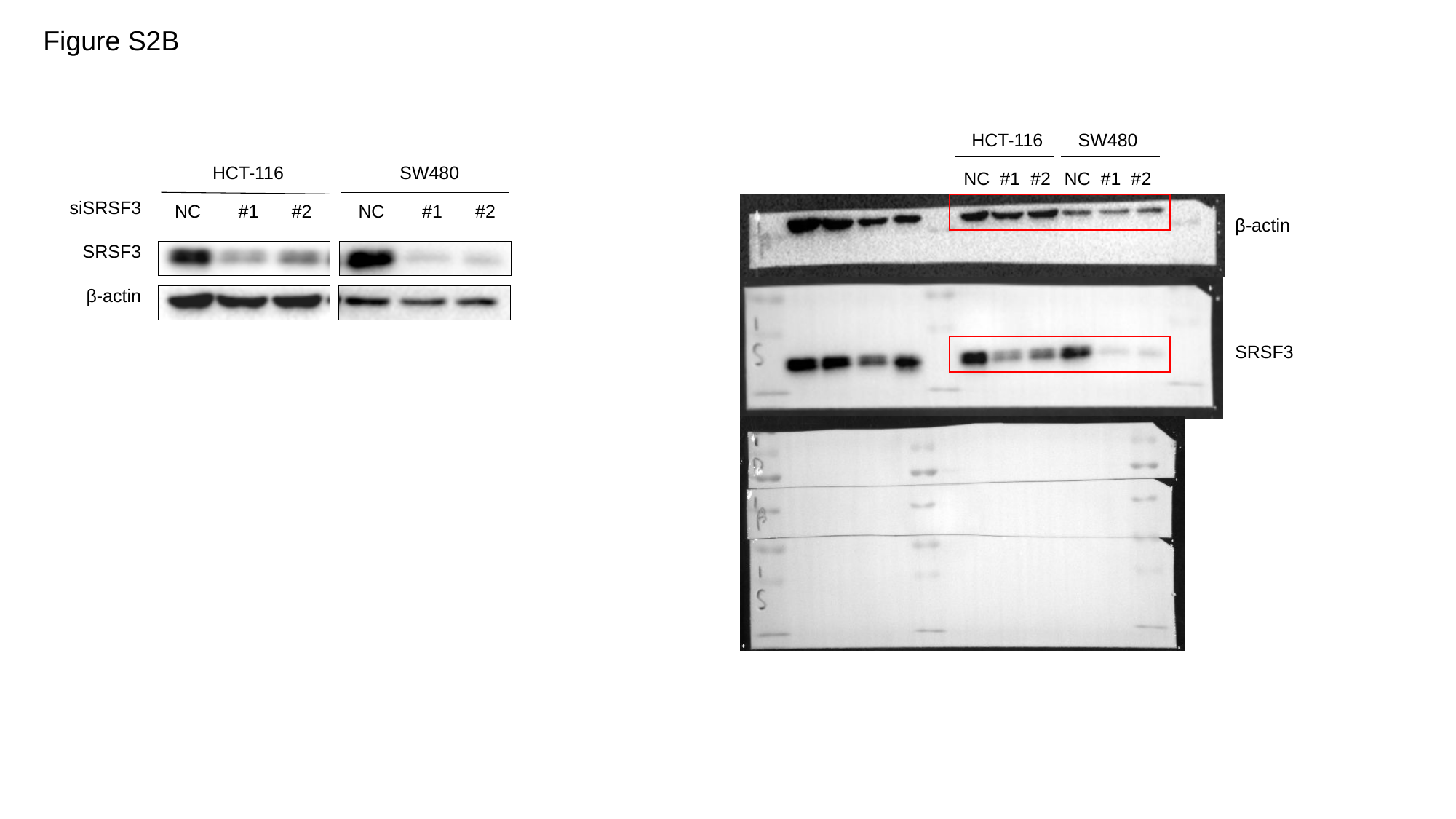

Figure S2B
HCT-116
SW480
HCT-116
SW480
NC #1 #2
NC #1 #2
| siSRSF3 |
| --- |
| SRSF3 |
| β-actin |
| NC | #1 | #2 |
| --- | --- | --- |
| NC | #1 | #2 |
| --- | --- | --- |
β-actin
SRSF3

## Slide 47
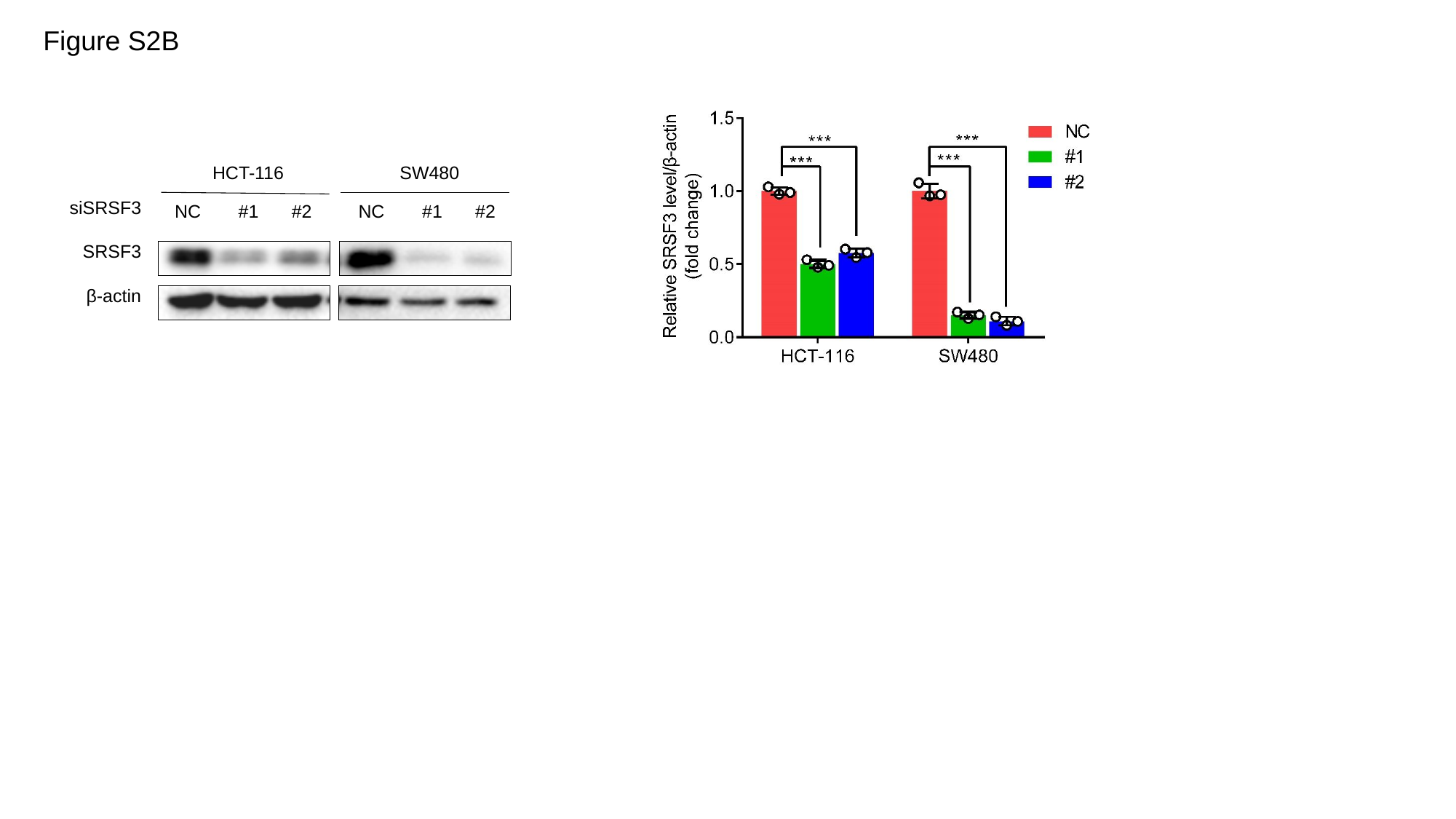

Figure S2B
HCT-116
SW480
| siSRSF3 |
| --- |
| SRSF3 |
| β-actin |
| NC | #1 | #2 |
| --- | --- | --- |
| NC | #1 | #2 |
| --- | --- | --- |

## Slide 48
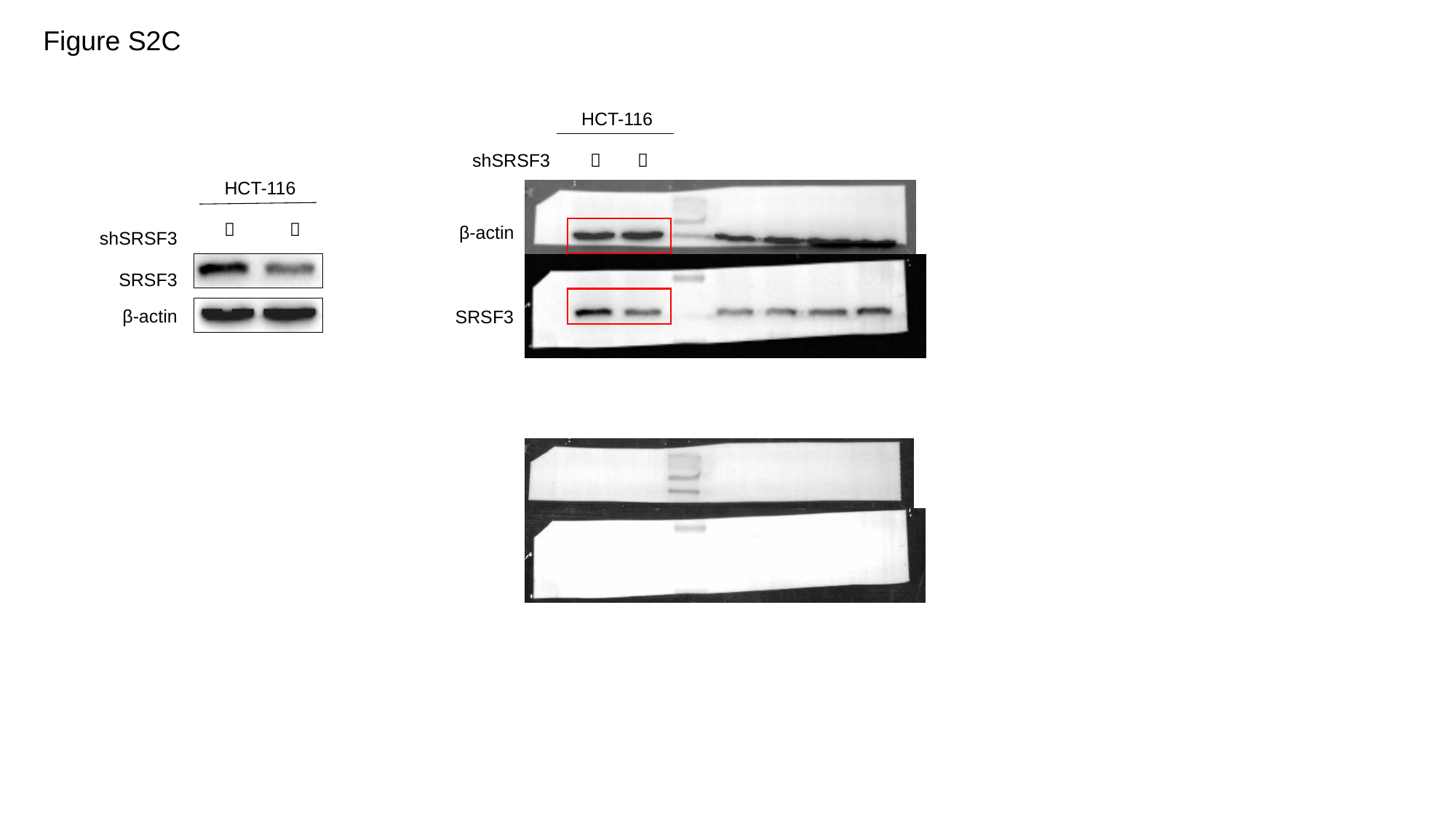

Figure S2C
HCT-116
| － | ＋ |
| --- | --- |
shSRSF3
HCT-116
| － | ＋ |
| --- | --- |
β-actin
| shSRSF3 |
| --- |
| SRSF3 |
| β-actin |
SRSF3

## Slide 49
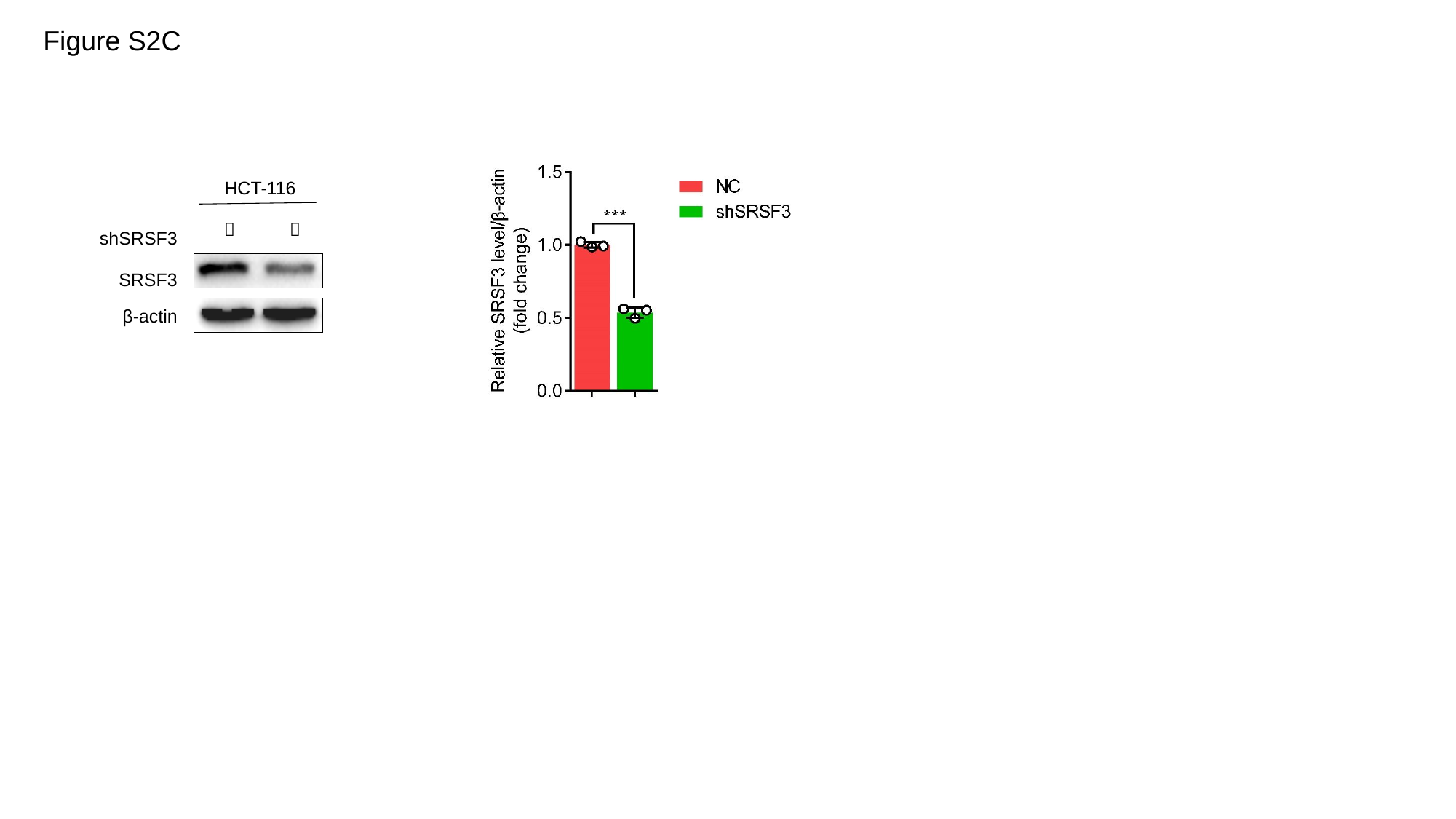

Figure S2C
HCT-116
| － | ＋ |
| --- | --- |
| shSRSF3 |
| --- |
| SRSF3 |
| β-actin |

## Slide 50
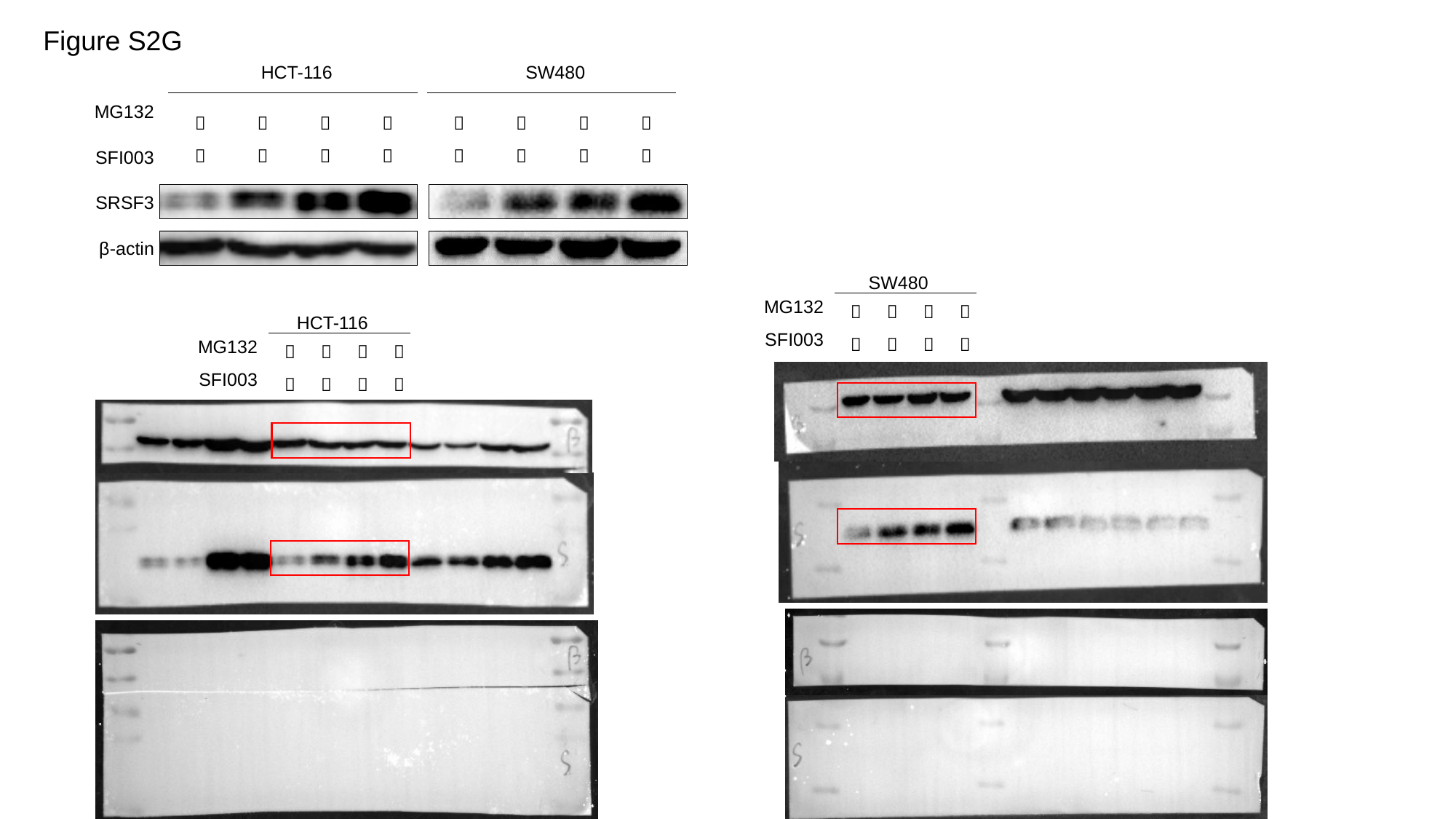

Figure S2G
HCT-116
SW480
| MG132 |
| --- |
| SFI003 |
| SRSF3 |
| β-actin |
| － | ＋ | － | ＋ |
| --- | --- | --- | --- |
| ＋ | ＋ | － | － |
| － | ＋ | － | ＋ |
| --- | --- | --- | --- |
| ＋ | ＋ | － | － |
SW480
| MG132 |
| --- |
| SFI003 |
| － | ＋ | － | ＋ |
| --- | --- | --- | --- |
| ＋ | ＋ | － | － |
HCT-116
| MG132 |
| --- |
| SFI003 |
| － | ＋ | － | ＋ |
| --- | --- | --- | --- |
| ＋ | ＋ | － | － |

## Slide 51
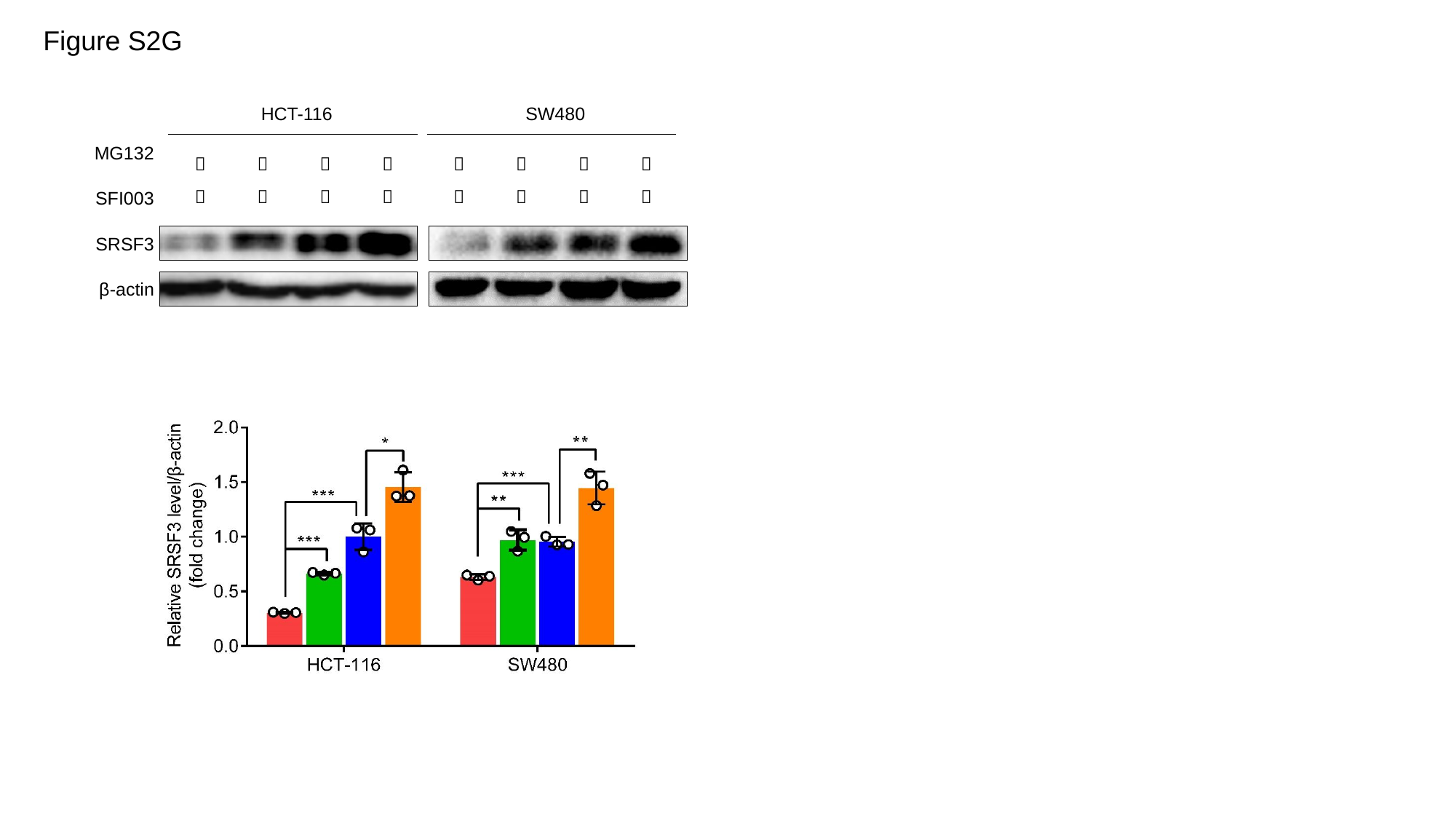

Figure S2G
HCT-116
SW480
| MG132 |
| --- |
| SFI003 |
| SRSF3 |
| β-actin |
| － | ＋ | － | ＋ |
| --- | --- | --- | --- |
| ＋ | ＋ | － | － |
| － | ＋ | － | ＋ |
| --- | --- | --- | --- |
| ＋ | ＋ | － | － |

## Slide 52
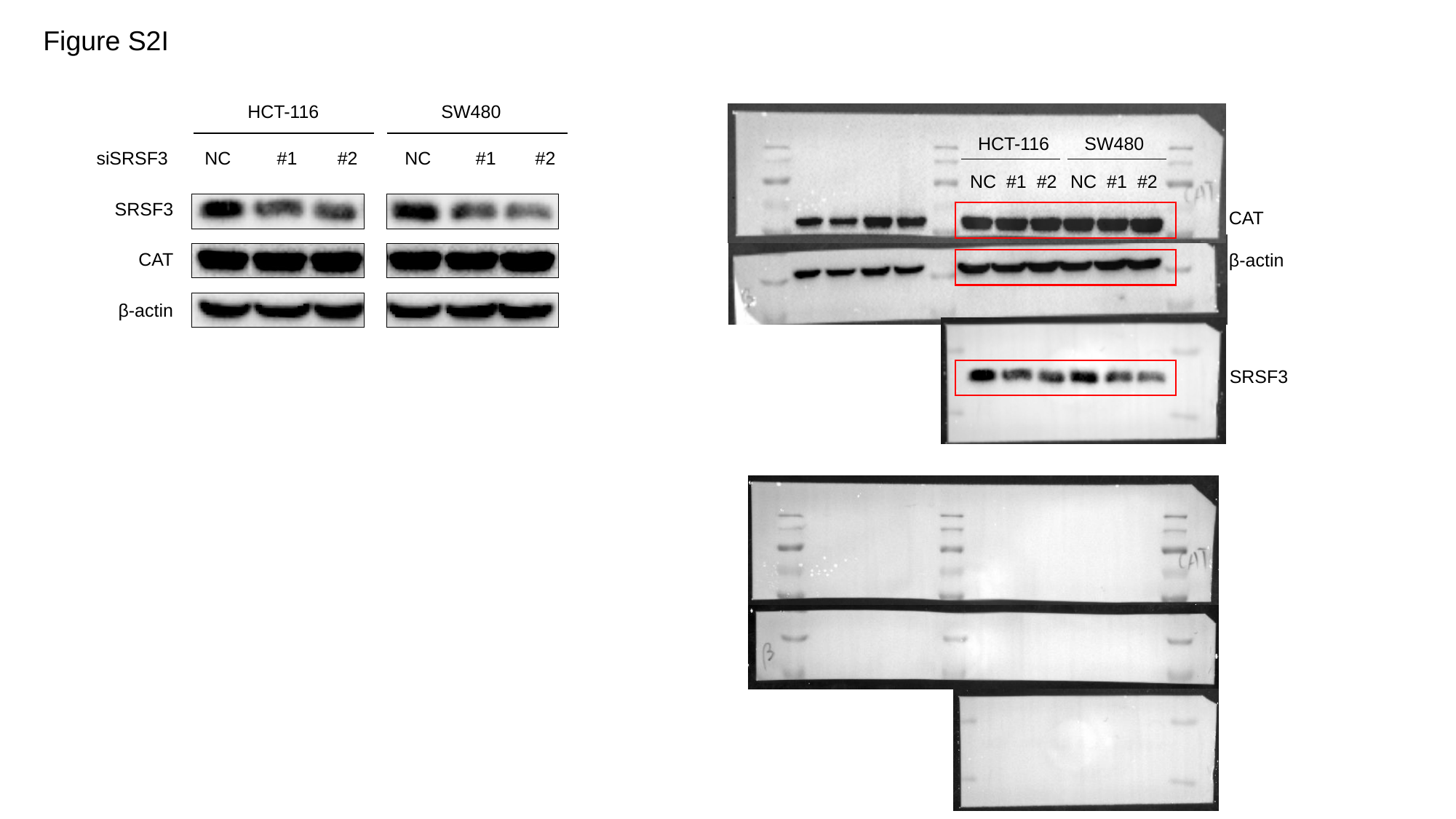

Figure S2I
HCT-116
SW480
HCT-116
SW480
| NC | #1 | #2 |
| --- | --- | --- |
| NC | #1 | #2 |
| --- | --- | --- |
| siSRSF3 |
| --- |
| SRSF3 |
| CAT |
| β-actin |
NC #1 #2
NC #1 #2
CAT
β-actin
SRSF3

## Slide 53
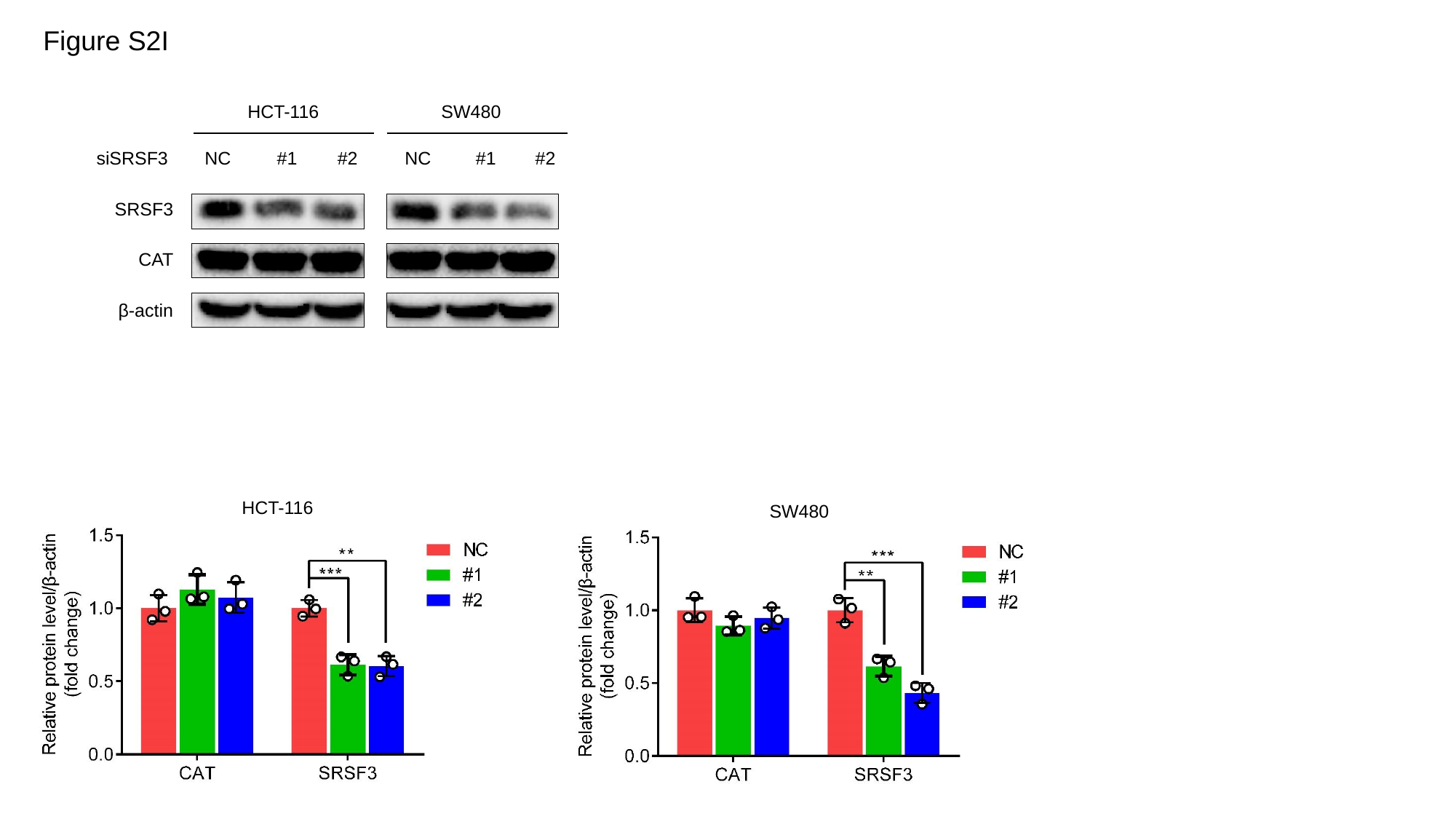

Figure S2I
HCT-116
SW480
| NC | #1 | #2 |
| --- | --- | --- |
| NC | #1 | #2 |
| --- | --- | --- |
| siSRSF3 |
| --- |
| SRSF3 |
| CAT |
| β-actin |
HCT-116
SW480
